# Oxygenated Cembrene Diterpenes from *Sarcophyton convolutum:* Cytotoxic Sarcoconvolutum A–E

**DOI:** 10.3390/md19090519

**Published:** 2021-09-13

**Authors:** Tarik A. Mohamed, Abdelsamed I. Elshamy, Asmaa M. Abdel-Tawab, Mona M. AbdelMohsen, Shinji Ohta, Paul W. Pare, Mohamed-Elamir F. Hegazy

**Affiliations:** 1 Chemistry of Medicinal Plants Department, National Research Centre, 33 El-Bohouth St., Dokki, Giza 12622, Egypt; ta.mourad@nrc.sci.eg (T.A.M.); monaamohsen@yahoo.com (M.M.A.); 2 Department of Natural Compounds Chemistry, National Research Centre, 33 El-Bohouth St., Dokki, Giza 12622, Egypt; ai.el-shamy@nrc.sci.eg; 3 Marine Biotechnology and Natural Products Laboratory, National Institute of Oceanography and Fisheries, Cairo 11516, Egypt; am.eltawab@niof.sci.eg; 4 Graduate School of Integrated Sciences for Life, Hiroshima University, 1-7-1 Kagamiyama, Higashi-Hiroshima 739-8521, Japan; ohta@hiroshima-u.ac.jp; 5 Department of Chemistry & Biochemistry, Texas Tech University, Lubbock, TX 79409, USA; 6 Department of Pharmaceutical Biology, Institute of Pharmaceutical and Biomedical Sciences, Johannes Gutenberg University, Staudinger Weg 5, 55128 Mainz, Germany

**Keywords:** *Sarcophyton convolutum*, sarcoconvolutum A–E, cembrenoids, cytotoxicity

## Abstract

The soft coral genus *Sarcophyton* contains the enzymatic machinery to synthesize a multitude of cembrene-type diterpenes. Herein, highly oxygenated cembrenoids, sarcoconvolutum A–E (**1**–**5**) were purified and characterized from an ethyl acetate extract of the red sea soft coral, *Sarcophyton convolutum*. Compounds were assemblies according to spectroscopic methods including FTIR, 1D- and 2D-NMR as well as HRMS. Metabolite cytotoxicity was tested against lung adenocarcinoma, cervical cancer, and oral-cavity carcinoma (A549, HeLa and HSC-2, respectively). The most cytotoxic compound, (**4**) was observed to be active against cell lines A549 and HSC-2 with IC_50_ values of 49.70 and 53.17 μM, respectively.

## 1. Introduction

Natural products and structural analogues are key for drug discovery, especially for pharmacotherapies for cancers and infectious diseases [1,2,3,4,5]. Such biologically active metabolites are characterized by scaffold diversity and structural complexity. For example, within *Sarcophyton* soft coral, isolated metabolites include cembrenoids [2,6,7,8,9], diterpene dimers [10,11], sesquiterpenes [12,13], ceramides [14], steroids [15,16], and prostaglandins [17,18]. Cembrenoids in particular exhibit a significant number of sp^3^ carbon centers and oxygen atoms with no nitrogen or halogen atoms. Higher numbers of H-bond acceptors and donors confer hydrophilicity and ring structures provide significant molecular rigidity. Cembrenoids also exhibit promising biological activities including anticancer [6,7,8], anti-inflammatory [8], antifouling [19,20], ichthyotoxic [21], antifeedant [12], antiviral [22], and neuroprotective [23] properties.

**Figure 1:**
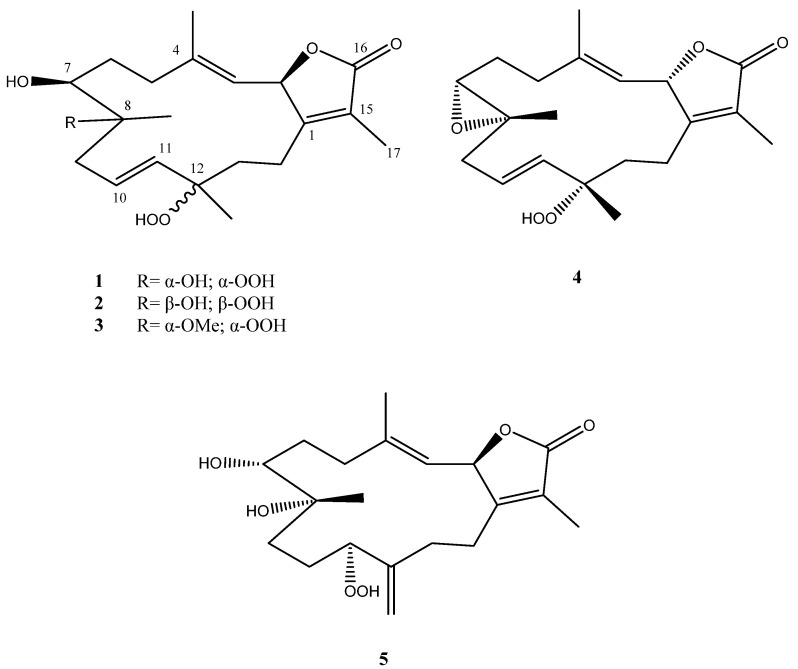
Sarcoconvolutum A–E (**1**–**5**) isolated from *S. convolutum*.

In a pursuit of novel metabolites with biological activity, polyoxygenated cembrene-type diterpenoids (Figure 1) have been isolated from the Red Sea soft coral *S. convolutum*. Metabolite antiproliferative activity was assayed against a non-small-cell lung adenocarcinoma, a cervical cancer, and an oral-cavity squamous-cell carcinoma, A549, HeLa, and HSC-2, respectively.

## 2. Results and Discussion

**Figure 2:**
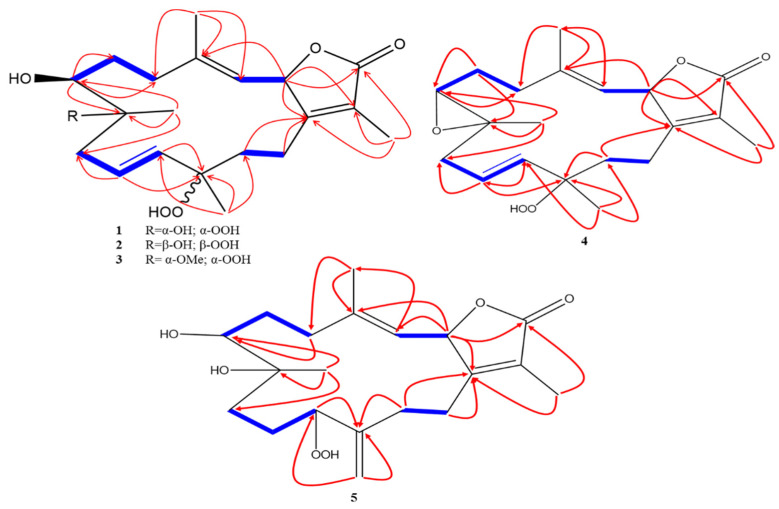
Key HMBC and ^1^H ^1^H COSY of sarcoconvolutum A–E (**1**–**5**).

Sarcoconvolutum A (**1**) was isolated as yellow oil with a positive optical rotation (+10.6) in a MeOH solvent. The HRCIMS molecular ion peak at *m*/*z* 367.2122 [M + H]^+^ was assigned predicting a molecular formula of C_20_H_30_O_6_ (calcd. 367.2121, [M + H]^+^) with six degrees of unsaturation suggesting a bicyclic skeleton. FTIR spectrum exhibited bands at 3465 cm^−1^ and 1765 cm^−1^ corresponding to hydroxyls and carbonyl groups. From ^1^H NMR spectrum (Table 1), two oxygenated protons at *δ*_H_ 3.26 br d (*J* = 10.8 Hz) and 5.53 d (*J* = 10.4 Hz), three olefinic protons at *δ*_H_ 4.84 br d (*J* = 10.4 Hz), 5.48 br d (*J* = 16.3 Hz), and 5.50 m, in addition to four methyls were at *δ*_H_ 1.28 s, 1.37 s, 1.83 s, and 1.87 s were characterized. Twenty carbon resonances were predicted from ^13^C NMR (Table 1) and categorized into 6 quaternary (including carbonyl at *δ*_C_ 175.3; three olefinics at *δ*_C_ 123.1, 143.9 and 162.3; alongside of two oxygenated carbons at *δ*_C_ 74.5 and 84.1), five methines (comprising two oxygenated carbons at *δ*_C_ 71.6 and 79.4; as well as three olefinic at *δ*_C_ 121.1, 127.8, and 134.8), five methylenes, four methyls (*δ*_C_ 8.8, 16.5, 20.3, and 24.2) depending upon DEPT and HSQC experiments. The above analyses of 1D NMR of **1** revealed the cembrene-based diterpenoid [6,7,24]. The 1D NMR of 1 was similar to 12-hydroperoxylsarcoph-10-ene isolated previously from *S. glaucum* [24]. Observed signal differences for the newly isolated compound included a down field shift of H-7 and C-7 by 0.73 and 12.1 ppm, respectively; a down field shift of 15.3 ppm for C-8; and a down field shift of 6 ppm for Me-19 consistent with the substitution of a hydroxyl group at C-7 instead of an epoxide ring coupling C-7 and C-8. The proposed structure was confirmed by ^1^H ^1^H COSY and HMBC spectral analysis. H-2 [*δ*_H_ 5.53 d (*J* = 10.4 Hz)]/C-4 (*δ*_C_ 143.9, *J*^3^), and ^1^H ^1^H COSY of H-2/H-3 [*δ*_H_ 4.84 br d (*J* = 10.4 Hz)] indicated the location of Δ^3(4)^. The HMBC correlation of H_3_-18 (*δ*_H_ 1.87 s) and C_H2_-5 (*δ*_C_ 35.2, *J*^3^) followed by ^1^H ^1^H COSY correlations of H_2_-5 [*δ*_H_ 2.18 br d (*J* = 12.7 Hz)]/ H-6 (*δ*_H_ 1.77 br d (*J* = 13.4 Hz)], H-6/H-7 (*δ*_H_ 3.26 br d (*J* = 10.8 Hz) confirmed a hydroxyl group at C-7. HMBC correlation of H_3_-19 (*δ*_H_ 1.28 s)/C-7 (*δ*_C_ 71.6, *J*^3^), H_3_-19/C-8 (*δ*_C_ 74.5, *J*^3^), and H-7/C-8 (*J*^2^) established hydroxylation of the C-8 quaternary carbon. Subsequently, HMBC correlation of H_3_-19/C_H2_-9 (*δ*_C_ 42.9, *J*^3^), H_3_-20 (*δ*_H_ 1.37 s)/C_H2_-11 (*δ*_C_ 134.8, *J*^3^), in addition to ^1^H ^1^H COSY of H_2_-9 [*δ*_H_ 2.34 m]/H-10 (*δ*_H_ 5.50 m), H-10/H-11 [(*δ*_H_ 5.48 br d (*J* = 16.3 Hz)] established Δ^10(11)^. The down field shift of C-12 by ca. 7 ppm [24] as well as the HMBC correlation of H-11/C-12 (*δ*_C_ 84.1, *J*^3^), H_3_-20/C-12 (*J*^2^), and H_3_-20/C-13 (*δ*_C_ 34.4 *J*^3^) indicated the presence of a hydroperoxide group at C-12. The C-1/C-2 included lactone ring was established via HMBC of H-2/C-16 (keto group, *δ*_C_ 175.3, *J*^3^), H-2/C-15 (*δ*_C_ 123.1, *J*^3^), H_3_-17 (*δ*_H_ 1.83 s)/C-16 (*J*^3^), H_3_-17/C-1 (*δ*_C_ 162.3, *J*^3^), and H_3_-17/C-15 (Figure 2).

**Figure 3:**
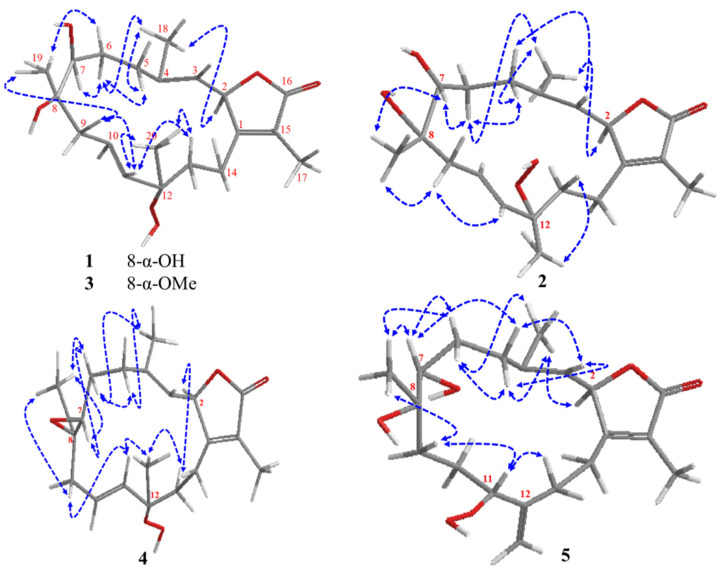
Significant NOESY of sarcoconvolutum A–E (**1**–**5**).

The relative configuration of 1 is based on coupling constants and NOESY data (Figure 3). From the coupling constant of H-2 (10.4 Hz) along with the vicinal coupling of H-3 (10.4 Hz), the *cis* orientation and *α* configuration of H-2 was established [7,24,25]. Starting from this point, the NOESY correlations of H-2*α* [*δ*_H_ 5.53 d (*J* = 10.4 Hz)]/ H_3_-18 [*δ*_H_ 1.87 s], H_3_-18/ H-5*α* [*δ*_H_ 2.18 br d (*J* = 12.7 Hz)], H_3_-18/ H-6*α* [*δ*_H_ 1.77 br d (*J* = 13.4)], H-5*α*/ H-6*α*, and H-6*α*/ H-7 [*δ*_H_ 3.26 br d (*J* = 10.8 Hz)] established an *α* orientation for H-7. NOESY correlations of H-6β [*δ*_H_ 1.41 ddd (*J* = 13.7, 12.7, 10.9 Hz)]/ H_3_-19 (*δ*_H_ 1.28 s), H-9β (*δ*_H_ 2.34 m)/ H_3_-19, H-9β/ H-11 [*δ*_H_ 5.48 br d (*J* = 16.3 Hz)], H-11/ H-13β [*δ*_H_ 1.72 br t (*J* = 13.2 Hz)], H-13β/ H_3_-20 [*δ*_H_ 1.37 s], H-11/ H_3_-20 indicated β configurations for both Me-19 and Me-20. Based on the described spectral analysis, **1** was identified as 7β,8α-dihydroxy-12α-hydroperoxide-16-keto-cembra-1*E*,3*E*,10*E*-triene (sarcoconvolutum A).

Sarcoconvolutum B (**2**) was isolated as yellow oil with a positive optical rotation (+43.1) in MeOH. From the HRCIMS molecular ion peak at *m/z* 367.2122 [M + H]^+^, the molecular formula of C_20_H_30_O_6_ (calcd. 367.2121, [M + H]^+^) a bicylic skeleton with six degrees of unsaturation was predicted. The FTIR broad bands corresponding to hydroxyls and carbonyl groups were detected at 3462 cm^−1^ and 1768 cm^−1^. The ^1^H NMR spectrum (Table 1) displayed two oxygenated protons at *δ*_H_ 3.32 d (*J* = 10.9 Hz) and 5.45 d (*J* = 10.2 Hz), three olefinic protons at *δ*_H_ 4.88 d (*J* = 10.1 Hz), 5.50 d (*J* = 16.1 Hz), and 5.57 dt (*J* = 16.1, 7.2 Hz), along with four methyls were at *δ*_H_ 1.28 s, 1.42 s, 1.82 s, 1.85 s. From the observed twenty signals in the ^13^C NMR spectrum (Table 1), 6 quartenary (including carbonyl at *δ*_C_ 174.8; three olefinics at *δ*_C_ 123.5, 143.5 and 161.9; alongside of two oxygenated carbons at *δ*_C_ 74.5 and 84.0), 5 methenes (comprising two oxygenated carbons at *δ*_C_ 78.7 and 71.1, as well as three olefinic signals at *δ*_C_ 121.6, 126.9, 134.6), 5 methylenes, 4 methyls (*δ*_C_ 8.9, 16.1, 23.6, and 24.1) were identified based on DEPT and HSQC signals. A down field shift of *δ*_C_ 84.0 by ca. 7 ppm suggested the presence of a hydroperoxyl unit at C-12 [24] which was consistent with mass spectral data. A ^13^C NMR comparison of the two isolated compounds indicated a down field shift of Me-20 for **2**. The 2D NMR comparisons showed otherwise similar ^1^H ^1^H COSY and HMBC signals (Figure 2). A ^13^C NMR down field shift of 3.3 ppm for Me-20 indicated the opposite stereochemistry at C-12.

Relative stereochemistry was determined based on coupling constants and NOESY data (Figure 3). The coupling constant of H-2 (10.2 Hz) in addition to the vicinal coupling of H-3 (10.1 Hz) suggested a *cis* orientation and *α* configuration of H-2 [7,24,25]. The NOESY correlations of H-2*α* [*δ*_H_ 5.45 d (*J* = 10.2 Hz)]/ H_3_-18 [*δ*_H_ 1.82 s], H-18/ H-5 [*δ*_H_ 2.37 td (*J* = 13.2t, 2.8 Hz)], H-18/ H-6 [*δ*_H_ 1.49 td (*J* = 13.7t, 3.6 Hz)], H-5*α*/ H-6, H-6/ H-7 [*δ*_H_ 3.32 br d (*J* = 10.9 Hz)], H-7/ H-19 [*δ*_H_ 1.28 s] and H-13β [*δ*_H_ 1.95 td (*J* = 13.1, 4.1 Hz)]/ H_3_-19 indicated a H-7*α* Me-19 and Me-20orientation. Based upon these spectral data, **2** was identified as 7β,8α-dihydroxy-12β-hydroperoxide-16-keto-cembra-1*E*,3*E*,10*E*-triene (sarcoconvolutum B).

Sarcoconvolutum C (**3**) was isolated as yellow oil with a positive optical rotation (+33.1, *c* 0.003, CH_3_OH). The molecular formula was deduced as C_21_H_32_O_6_ (calcd. 364.2250, [M-OH + H]^+^) based on a HREIMS molecular ion peak at *m*/*z* 364.2258 [M-OH + H]^+^ indicating a bicylic skeleton with six degrees of unsaturation. FTIR broad bands corresponded to hydroxyls and carbonyl groups were detected at 3457 cm^−1^ and 1761 cm^−1^. From ^1^H NMR spectrum (Table 1), two oxygenated protons at *δ*_H_ 3.27 br d (*J* = 10.9 Hz) and *δ*_H_ 5.49 d (*J*= 9.9 Hz), three olefinic protons at *δ*_H_ 4.86 br d (*J* = 9.9 Hz), *δ*_H_ 5.44 br d (*J* = 16.1 Hz) and *δ*_H_ 5.56 dt (*J* = 15.6, 8.1t Hz), four methyls as well as one methyl of methoxy group at *δ*_H_ 3.23 s were assigned. Based on the ^13^C NMR (Table 1), twenty-one carbon resonances were observed and were categorized by DEPT and HSQC analysis as 6 quaternary carbons (including carbonyl at *δ*_C_ 174.8; three olefinics at *δ*_C_ 123.3, 144.2 and 161.4; alongside of two oxygenated carbons at *δ*_C_ 78.3 and 84.4), 5 methines (comprising two oxygenated carbons at *δ*_C_ 78.3 and 71.9; as well as three olefinic at *δ*_C_ 121.2, 128.2, 134.0), 5 methylenes, 5 methyls (*δ*_C_ 9.0, 16.2, 18.2, 21.2, and 49.3). As with **1** and **2**, down field shift at *δ*_C_ 84.4 ppm of ca. 7 ppm along with a mass shift of [M-H_2_O], suggested the addition of a hydroperoxy group [24]. The compound is similar to **1** and **2** except for a down field shift of *δ*_H_ 78.3 by 3.8 ppm associated with C-8 and a new methoxyl signal at *δ*_H_ 3.23 s and *δ*_C_ 49.3. HMBC correlations of a methyl proton at *δ*_H_ 3.23 and the C-12 at *δ*_H_ 78.3 confirm the localization of the methoxylation to C-12 (Figure 2). Thus, **3** was identified as 7-hydroxy-8-methoxy-12-hydroperoxide-16-keto-cembra-1*E*,3*E*,10*E*-triene.

The relative configuration of **3** was deduced via the coupling constants and NOESY analysis (Figure 3). The coupling constant of H-2 (9.9 Hz) and the vicinal coupling of H-3 (9.9 Hz) identified an *α* and *cis* orientation for H-2 [7,24,25]. The NOESY experiments exhibited the same configuration of **1** that elucidated the *α* orientation of H-7 and β and both methyls, Me-19 and Me-20. Based on these spectral observations, **3** was identified as 7β-hydroxy-8α-methoxy-12α-hydroperoxide-16-keto-cembra-1*E*,3*E*,10*E*-triene (sarcoconvolutum C).

Sarcoconvolutum D (**4**) was obtained as dark yellow oil with a positive optical rotation (+37.2, *c* 0.003, CH_3_OH). The HREIMS molecular ion peak at *m*/*z* 348.1939 [M]^+^ that predicted a molecular formula of C_20_H_28_O_5_ (calcd. 348.1937, [M]^+^) with a bicylic skeleton and seven degrees of unsaturation. The corresponding FTIR broad bands to hydroxyls and carbonyl groups at 3456 cm^−1^ and 1763 cm^−1^ were assigned. The ^1^H NMR (Table 1) revealed two oxygenated protons at *δ*_H_ 2.65 dd (*J* = 6.8, 3.6 Hz), and 5.45 d (*J* = 9.4 Hz), three olefineic protons at *δ*_H_ 5.00 d (*J* = 9.4 Hz), 5.42 m, and 5.47 m, in addition to four methyls at *δ*_H_ 1.31 s, 1.41 s, 1.86 s, and 1.87 s. From ^13^C NMR, twenty carbon resonances were characterized and categorized to six quartenary (consisting of carbonyl at *δ*_C_ 174.8; three olefinics at *δ*_C_ 123.6, 144.4, and 161.7; and two oxygenated carbons at *δ*_C_ 60.0 and 84.6, five methenes (comprising two oxygenated carbons at *δ*_C_ 57.0, and 78.8; and two olefinic carbon (at *δ*_C_ 125.8 and 135.5), five methylenes, four methyls based on DEPT-135 and HSQC analysis. The compound is very similar to **1** except for an up-field shift of *δ*_H_ 2.65 dd (*J* = 6.8, 3.6 Hz) by 0.67 ppm and an up-field shift of *δ*_C_ 57.0 by 14.6 ppm both associated with C-7 and an up-field shift of *δ*_C_ 60 by 14.5 ppm associated with C-8. These chemical shift changes were deduced to be a result of a C-7/C-8 epoxide ring that was consistent with a molecular ion peak at *m*/*z* 348.1939 (C_20_H_28_O_5_). The localization of this group was derived via the HMBC correlations of Me-19 (*δ*_H_ 1.31 s)/C-7 (*J*^3^), Me-19/C-8 (*J*^2^), H-6 (*δ*_H_ 1.60 m)/C-7 (*J*^2^), and H-6/C-8 (*J*^3^) (Figure 2).

The relative stereochemistry was identified via coupling constants [7,24,25] and NOESY analysis (Figure 3). Compound **4** exhibited almost same NOESY correlations as **1** except the correlations of Me-19/H-7, and H-6β/H-7 that elucidated the β orientation of H-7. These data confirmed **4** as 12α-hydroperoxide-16-keto-cembra-1*E*,3*E*,10*E*-triene-7α, 8α-epoxide (sarcoconvolutum D).

Sarcoconvolutum E (**5**) was obtained as yellow oil exhibiting a positive optical rotation (+61.7, *c* 0.003, CH_3_OH). The mass spectroscopy exhibited a HREIMS molecular ion peak at *m*/*z* 367.2130 [M + H]^+^ that was identified as C_20_H_30_O_6_ (calcd. 367.2042, [M + H]^+^) with a bicylic skeleton and six degrees of unsaturation. The FTIR broad bands corresponding to hydroxyls and carbonyl groups were detected at 3464 cm^−1^ and 1759 cm^−1^. ^1^H NMR (Table 1) displayed three oxygenated protons at *δ*_H_ 3.45 br d (*J* = 10.5 Hz), 4.43 dd (*J* = 9.0, 5.0 Hz), and 5.47 d (*J* = 10.1 Hz), one olefineic proton at *δ*_H_ 4.92 br d (*J* = 10.1 Hz), one exomethylene proton at *δ*_H_ 5.07 s, and 5.13 s, and three methyles at *δ*_H_ 1.26 s, 1.86 s (6H). The ^13^C NMR showed twenty carbon resonances. Six quaternary (including carbonyl at *δ*_C_ 174.9; four olefinics at *δ*_C_ 124.3, 145.9, 146.0 and 161.8; and one oxygenated carbon at *δ*_C_ 73.9), four methenes (comprising two oxygenated carbons at *δ*_C_ 70.0 and 89.3; and one olefinic carbon (at *δ*_C_ 119.2), 7 methylenes (comprising exomethylene carbon at *δ*_C_ 113.4), three methyls (*δ*_C_ 8.9, 16.4, and 24.8) that were characterized via DEPT-135 and HSQC experiments. NMR spectral data of **5** were similar to **1** and **2** except for the following signals: (i) a down-field shift of *δ*_C_ 75.0, by ca. 3.5 ppm associated with C-7; (ii) an downfield shift of *δ*_H_ 3.45 brd (*J* = 10.5 Hz) by 0.2 ppm associated with H-7; and (iii) an up-field shift of *δ*_H_ 4.43 dd (*J* = 9.0, 5.0 Hz) by 1 ppm and a shift of ca. 45 ppm of *δ*_C_ 134.0 to 89.0 associated with C-11 indicating the oxygenation of this carbon; (iv) the appearance of *δ*_H_ 1.32 (m, 1.57) associated with H-10 and C-10 (at *δ*_C_ 25.0) indicating a disappearance of Δ^10(11)^; and (v) the presence of *δ*_C_ 146.0 associated with C-12 and signals *δ*_H_ 5.07 s, 5.13 s and *δ*_C_ 113.4 associated with an exomethylene carbon at C-20 indicating the presence of Δ^12(20)^. These new functional group assignments based on spectral data were consistent with 2D HMBC spectral signals including H_3_-19 [*δ*_H_ 1.26 s]/C-7 [*δ*_C_ 75.0, *J*^3^], H_3_-19/C-7 [*δ*_C_ 73.9, *J*^2^] confirmed the hydroxylation of both C-7 and C-8. The HMBC correlations of H_2_-20 [at *δ*_H_ 5.07 s, 5.13 s]/C-12 [at *δ*_C_ 146.0, *J*^2^], H_2_-20/C-11 [at *δ*_C_ 89.0, *J*^3^], and H_2_-20/C-13 [at *δ*_C_ 27.8, *J*^3^], H-11 [at *δ*_H_ 4.43 dd (*J* = 9.0, 5.0 Hz)]/C-12 [*J*^2^], H-11/C-13 [*J*^3^] confirmed the Δ^12(20)^ and the oxygenation of C-11 (Figure 2). Consistent with the mass spectral data and the observed up-field shift of C-11, the oxygenation of C-11 was identified as a hydroperoxide group.

The relative stereochemistry assignment was based the coupling constant of H-2 (10.1 Hz) and the vicinal coupling of H-3 (10.1 Hz) the indicated a *α* and *cis* orientation of H-2 [7,24,25]. NOESY correlations (Figure 3) of H-2α [*δ*_H_ 5.47 d (*J* = 10.1 Hz)]/ H_3_-18 [*δ*_H_ 1.86 s], H-3α [*δ*_H_ 4.92 br d (*J* = 10.1 Hz)]/ H-5α [*δ*_H_ 2.35 dt (*J* = 13.0, 7.6t Hz)], and H-5α/ H-6 [*δ*_H_ 1.75 m], H_3_-18/ H-6, H-5/ H-7 [*δ*_H_ 3.45 br d (*J*= 10.5 Hz)], H-6/ H-7, H-7/ H_3_-19 [*δ*_H_ 1.26 s], H_3_-19β/ H-9β [*δ*_H_ 1.51 m], H-9β/ H-11 [*δ*_H_ 4.43 dd (*J* = 9.0, 5.0 Hz)], H-13β [*δ*_H_ 2.25 m]/ H-identified a β orientation for H-7, H-11 and Me-19. Therefore, **5** was assigned as 7α,8α-dihydroxy-11α-hydroperoxide-16-keto-cembra-1E,3E,12(20)-triene (sarcoconvolutum E). 1D, 2D-NMR, HRMS and FTIR for all isolated secondary metabolites (1–5) were deposited in the supplementary files (see Figures S1–S50).

**Figure 4:**
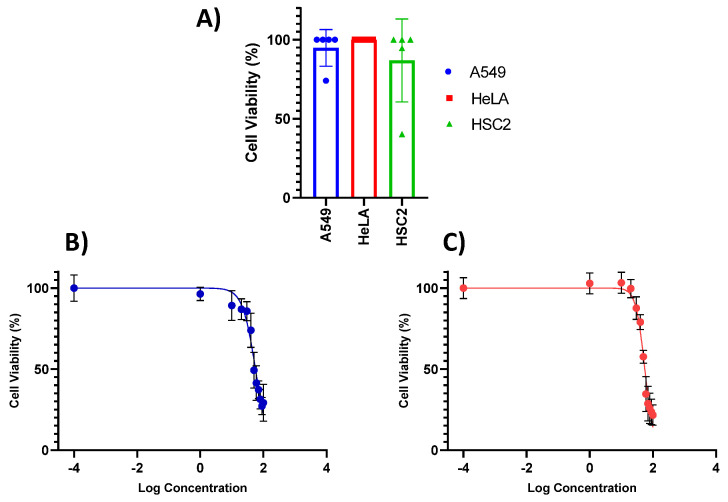
Cytotoxic activity: (**A**) Screening cytotoxic activity against A549, HeLa and HSC2 cell lines; (**B**) Dose-dependent toxicity with concentrations in a range from 0.001 to 100 μM for compounds **4** against A549; (**C**) Dose-dependent toxicity with concentrations in a range from 0.001 to 100 μM for compound 4 against HSC2.

The initial screening of **1**–**5** (Figure 4) for cytotoxic activity was performed using three cancer cell lines (A549, HeLa and HSC-2) with concentrations of 100, 10 and 1 μM (Figure 4A). Compound **4** showed cytotoxic activity against cell lines A549 (Figure 4B) and HSC-2 (Figure 4C). The dose-dependent toxicity with concentrations range (0.001–100 μg/mL) was examined against A549 and HSC-2 cell lines (Figure 4). Compound **4** showed IC_50_ values of 49.70 and 53.17 μM against A549 and HSC-2 cells, respectively (Table 2, Figure 4A,B).

## 3. Materials and Methods

### 3.1. General Experimental Procedures

A JASCO P-2300 polarimeter (Tokyo, Japan) and shimazu FTIR-8400S instrument (Columbia, MD 21046, USA) was operated for optical rotation and IR spectra, respectively. Then, 1D and 2D NMR spectra were recorded on a Bruker 600 or 500 Hz NMR spectrometer (MA, USA). HR-MS spectra were obtained on a JEOL JMS-700 instrument (Tokyo, Japan). For Chromatography: column chromatography (CC) [silica gel 60 (Merck, 230–400 mesh, Merck, Darmstadt, Germany)]; TLC analysis: [precoated silica gel plates (Merck, Kieselgel 60 F_254_, 0.25 mm, Merck, Darmstadt, Germany]. High-performance liquid chromatography (HPLC) equipped with a Jasco PU-980 pump, a Jasco UV-970 intelligent UV/VIS detector at 210 nm and a semi preparative reversed-phase column (Cosmosil C_18_ column 250 × 10 mm, 5 μm).

### 3.2. Coral Material

The animal soft coral *Sarcophyton convolutum* was collected from the Egyptian Red Sea (the coast of Hurghada) in March 2017 and was recognized by M Al-Hammady with a voucher specimen (08RS1071). It was deposited in the National Institute of Oceanography and Fisheries, marine biological station, Hurghada, Egypt.

### 3.3. Extraction and Separation

The frozen soft coral (2.5 kg, total wet weight) was chopped into small pieces and extracted with ethyl acetate at room temperature (3 L × 5 times). The collective extracts were concentrated *in vacuo* to a brown gum. The dried material (95 g) was subjected to a silica gel column (6 × 120 cm) eluting with *n*-hexane (2000 mL), followed by a gradient of *n*-hexane-EtOAc up to 100% EtOAc and EtOAc –MeOH up to 50% MeOH (3000 mL each of the solvent mixture). Fractions were collected (SC1-SC6) and monitored via TLC. Fraction (SC3; 1.4 g) was eluted step gradient with *n*-hexane-EtOAc over silica gel column afforded three main sub fractions (SC3A-C). Sub-fraction SC3A was re-purified by reversed-phase HPLC using MeOH/H_2_O (6.5:3.5), 3.5 mL/min, to afford **1** (6.1 mg, *t*_R_ = 27 min), and **4** (11.6 mg, *t*_R_ = 23 min). The sub-fraction SC3B was subjected to reversed-phase HPLC using MeOH/H_2_O (3:2), 3.5 mL/min, afforded **2** (10 mg, *t*_R_ = 28 min). Fraction (SC4; 1.2 g) was further fractionated over silica gel column chromatography eluted by *n*-hexane/EtOAc step gradient afforded two main sub-fractions (SC4A and B). Sub-fraction SC4A was eluted with MeOH/H_2_O (1:1) over reversed-phase HPLC, 3 mL/min, to afford **3** (7.3 mg, *t*_R_ = 31 min), and **5** (9.6 mg, *t*_R_ = 32 min).

### 3.4. Spectral Data of Sarcoconvolutum A–E (1–5)

7,8-Dihydroxy-12-hydroxy-16-keto-cembra-1E,3E,10E-triene (Sarcoconvolutum A, **1**): yellow oil; [α]${}_{D}^{25}$ +43.0 (c 0.03, CH_3_OH); FT-IR (KBr) ν_max_: 3465, 2961, 1765, 1435, and 987 cm^−1^; ^1^H and ^13^C NMR data (CDCl_3_, 500 Hz), see Table 1; HRCIMS *m*/*z* 367.2122 (100, [M+H]^+^); (calcd. 367.2121, for C_20_H_31_O_6_).

7,8-Dihydroxy-12-hydroxy-16-keto-cembra-1E,3E,10E-triene (Sarcoconvolutum B, **2**): yellow oil; [α]${}_{D}^{25}$ +10.5 (c 0.03, CH_3_OH); FT-IR (KBr) ν_max_: 3461, 2957, 1759, 1439, and 991 cm^−1^; ^1^H and ^13^C NMR data (CDCl_3_, 500 Hz), see Table 1; HRCIMS *m*/*z* 367.2122 (100, [M+H]^+^); (calcd. 367.2122, for C_20_H_31_O_6_).

7β-Hydroxy-8α-methoxy-12α-hydroperoxide-16-keto-cembra-1E,3E,10E-triene (sarcoconvolutum C, **3**): yellow oil; [α]${}_{D}^{25}$ +37.2 (c 0.03, CH_3_OH); FT-IR (KBr) ν_max_: 3456, 2951, 1763, 1446, and 993 cm^−1^; ^1^H and ^13^C NMR data (CDCl_3_, 500 Hz), see Table 1; HRCIMS *m*/*z* 364.2258, [M-OH+H]^+^]; (calcd. 364.2250, [M-OH+H]^+^, for C_21_H_32_O_6_).

12α-Hydroperoxide-16-keto-cembra-1E,3E,10E-triene-7α,8α-epoxide (sarcoconvolutum D, **4**): yellow oil; [α]${}_{D}^{25}$ +37.2 (c 0.03, CH_3_OH); FT-IR (KBr) ν_max_: 3456, 2958, 1763, 1442, and 996 cm^−1^; ^1^H and ^13^C NMR data (CDCl_3_, 500 Hz), see Table 1; HREIMS *m*/*z* 348.1939 [M]^+^; (calcd. 348.1937, for C_20_H_28_O_5_).

7α,8α-Dihydroxy-11α-hydroperoxide-16-keto-cembra-1E,3E,12(20)-triene (sarcoconvolutum E, **5**): yellow oil; [α]${}_{D}^{25}$ +10.5 +61.7 (c 0.03, CH_3_OH); FT-IR (KBr) ν_max_: 3464, 2955, 1759, 1433, and 997 cm^−1^; ^1^H and ^13^C NMR data (CDCl_3_, 500 Hz), see Table 1; HREIMS *m*/*z* 367.2130 (100, [M+H]^+^); (calcd. 367.2042, for C_20_H_31_O_6_).

### 3.5. Cell Culture and Treatment Conditions

Human cancer cell lines of non-small cell lung adenocarcinoma (A549), squamous cell carcinoma of the oral cavity (HSC-2), and human cervical cancer cell (HeLa). All cell lines were purchased from American Type Culture Collection (ATCC^®^) and were maintained as monolayer culture in Dulbecco’s modified Eagle’s medium (DMEM) supplemented with 10% FBS, 4 mM l-glutamine, 100 U/mL penicillin, and 100 µg/mL streptomycin sulfate. Monolayers were passaged at 70–90% confluence using a trypsin-EDTA solution. All cell incubations were maintained in a humidified CO_2_ incubator with 5% CO_2_ at 37 °C. All materials and reagents for the cell cultures were purchased from Lonza (Verviers, Belgium).

### 3.6. Cytotoxicity Assay

A modified MTT (3-[4,5,[4,5-dimethylthiazole-2-yl]-2,5-diphenyltetrazolium bromide) assay was used for cytotoxicity investigation were performed using based on a previously published method [26]. A549, HeLa and HSC2 cells (5000–10,000 cells/well) were seeded onto 96-well plates and incubated for 24 h with 5% CO_2_ at 37 °C. The prepared tested compounds (**1**–**5**) were screened and the dose-dependent toxicity with concentrations range (0.001–100 μg/mL) were investigated according to our protocol [26]. Doxorubicin (2 mg/mL) was used as positive control.

### 3.7. Anti-Proliferation Quantitative Analysis

GraphPad Prism^®^ v6.0 software (GraphPad Software Inc., San Diego, CA, USA) were used to calculate the concentration-response curve fit to the non-linear regression for IC_50_ values.

## 4. Conclusions

Five compounds, sarcoconvolutum A–E were isolated from an organic extract of *Sarcophyton convolutum* tissue. Chemical structures were elucidated based upon spectroscopic analyses. The cytotoxic activity of the isolated compounds was screened against three cancer cell lines (A549, HeLa and HSC2) and **4** was found to be the most active compound against cell lines A549 and HSC-2 with IC_50_ values of 49.70 and 53.17 μM, respectively.

## Figures and Tables

**Table 1:** ^1^H and ^13^C NMR (CDCl_3_, 500 Hz) of sarcoconvolutum A–E (**1**–**5**).

No	Sarcoconvolutum A (1)		Sarcoconvolutum B (2)		Sarcoconvolutum C (3)		Sarcoconvolutum D (4)		Sarcoconvolutum E (5)	
	^1^H NMR (*J* Hz)	^13^C	^1^H NMR (*J* Hz)	^13^C	^1^H NMR (*J* Hz)	^13^C	^1^H NMR (*J* Hz)	^13^C	^1^H NMR (*J* Hz)	^13^C
1	----	162.3	----	161.9	----	161.4	----	161.7	----	161.8
2	5.53 d (10.4)	79.4	5.45 d (10.2)	78.7	5.49 d (9.9)	79.0	5.45 d (9.4) *	78.8	5.47 d (10.1)	80.2
3	4.84 br d (10.4)	121.1	4.88 br d (10.1)	121.6	4.86 br d (9.9)	121.2	5.00 d (9.4)	121.1	4.92 br d (10.1)	119.2
4	----	143.9	----	143.5	----	144.2	----	144.4	----	145.9
5α	2.18 br d (12.7)	35.2	2.23 dt (13.2, 3.4t)	35.3	2.18 dt (13.4, 3.4t)	35.4	2.26 m *	36.6	2.35 dt (13.0, 7.6t)	37.9
5β	2.40 t (11.3)		2.37 td (13.2t, 2.8)		2.37 td (13.4, 7.4)		2.34 td (13.1t, 5.7)		2.10 td (13.0t, 5.1)	
6α	1.77 br d (13.4)	26.7	1.49 td (13.7t, 3.6)	26.3	1.87 td (13.4t, 3.6)	26.7	1.60 m	24.1	1.75 m *	30.5
6β	1.51 ddd (13.7, 12.7, 10.9)		1.83 m *		1.44 td (15.3t, 4.3)		1.82 m *		1.25 m *	
7	3.26 br d (10.8)	71.6	3.32 br d (10.9)	71.1	3.27 br d (10.9)	71.9	2.65 dd (6.8, 3.6)	57.0	3.45 br d (10.5)	75.0
8	----	74.5	----	74.5	----	78.3	----	60.0	----	73.9
9α	2.34 m *	42.9	2.44 dd (13.7, 7.8)	43.4	2.34 m *	37.2	2.41 dd (13.1, 5.7)	39.0	1.57 m	35.1
9β	2.34 m *		2.28 dd (13.4, 6.6)		2.34 m *		2.46 dd (13.3, 7.1)		1.51 m	
10α	5.50 m	127.8	5.57 dt (16.1, 7.2)	126.9	5.56 dt (15.6, 8.1t)	128.2	5.42 m *	125.8	1.57 m	25.2
10β									1.32 m	
11	5.48 br d (16.3)	134.8	5.50 br d (16.1)	134.6	5.44 br d (16.1)	134.0	5.47 m *	135.5	4.43 dd (9.0, 5.0)	89.3
12	----	84.1	----	84.0	----	84.4	----	84.6	----	146.0
13α	1.72 br t (13.2)	34.4	1.49 td (13.7, 3.6)	36.4	1.80 td (13.4t, 3.5)	35.1	1.58 m *	36.0	2.02 br t (15.1)	27.8
13β	1.68 td (13.2t, 5.6)		1.95 td (13.1, 4.1)		1.67 td (13.4t, 4.7)		1.89 m *		2.25 m	
14α	2.01 br t (13.2)	22.7	2.06 br t (13.7)	21.6	2.05 br t (13.4)	22.7	2.07 br t	22.0	2.13 m	24.5
14β	2.43 br t (13.2)		2.62 td (13.7, 4.0)		2.40 td (13.7, 4.3)		2.27 td * (13.2t, 5.5)		2.65 td (12.9, 4.9)	
15	----	123.1	----	123.5	----	123.3	----	123.6	----	124.3
16	----	175.3	----	174.8	----	174.8	----	174.8	----	174.9
17	1.83 s	8.8	1.85 s	8.9	1.85 s	9.0	1.86 s	9.1	1.86 s	8.9
18	1.87 s	16.5	1.82 s	16.1	1.83 s	16.2	1.87 s	15.8	1.86 s	16.4
19	1.28 s	24.2	1.28 s	24.1	1.19 s	18.2	1.31 s	22.2	1.26 s	24.8
20	1.37 s	20.3	1.42 s	23.6	1.41 s	21.2	1.41 s	18.8	5.07 s, 5.13 s	113.4
OMe					3.23 s	49.3				

* overlapped.

**Table 2:** Cytotoxicity of 1-5 against cancer cell lines.

Cell Lines	(IC_50_, µM)					
	1	2	3	4	5	Doxorubicin
**A549**	˃100	˃100	˃100	49.70 ± 0.05	˃100	0.42 ± 0.3
**HeLa**	˃100	˃100	˃100	91.98 ± 0.15	˃100	1.35 ± 0.16
**HSC2**	˃100	˃100	˃100	53.17 ± 0.03	91.39 ± 0.17	0.50 ± 2.6

## Data Availability

The data presented in this study are available in Supplementary Material.

## Supplementary Materials

The following are available online https://www.mdpi.com/article/10.3390/md19090519/s1, Figures S1–S50: spectra for compounds **1**–**5**.
