# Supplementary material for: Oxygenated Cembrene Diterpenes from Sarcophyton convolutum: Cytotoxic Sarcoconvolutum A–E
Source: Mar Drugs. 2021 Sep 13;19(9):519. doi: 10.3390/md19090519 (PMC8467724; doi:10.3390/md19090519)
Supplement: Supplementary file 1 [file marinedrugs-19-00519-s001.zip › marinedrugs-1370098-supplementary.pdf]

Supplementary material

Oxygenated cembrene diterpenes from *Sarcophyton convolutum:* cytotoxic sarcoconvolutum A-E

Tarik A. Mohamed[1], Abdelsamed I. Elshamy[2], Asmaa M. Abdel-Tawab[3], Mona M. AbdelMohsen[1], Shinji Ohta[4], Paul W. Pare[5,*], Mohamed-Elamir F. Hegazy[1,6,*]

[1] Chemistry of Medicinal Plants Department, National Research Centre, 33 El-Bohouth St., Dokki, Giza 12622, Egypt; ta.mourad@nrc.sci.eg (T.A.M.); monaamohsen@yahoo.com (M.M.A.)
[2] Department of Natural Compounds Chemistry, National Research Centre, 33 El-Bohouth St., Dokki, Giza 12622, Egypt; ai.el-shamy@nrc.sci.eg (A.I.E.).
[3] Marine Biotechnology and Natural Products Laboratory, National Institute of Oceanography and Fisheries, Cairo, Egypt; ch_smsm84@yahoo.com, am.eltawab@niof.sci.eg (A.M.A.)
[4] Graduate School of Integrated Sciences for Life, Hiroshima University, 1-7-1 Kagamiyama, Higashi-Hiroshima 739-8521, Japan; ohta@hiroshima-u.ac.jp (S.O.).
[5] Department of Chemistry & Biochemistry Texas Tech University, Lubbock, TX 79409 USA; paul.pare@ttu.edu (P.W.P.).
[6] Department of Pharmaceutical Biology, Institute of Pharmaceutical and Biomedical Sciences, Johannes Gutenberg University, Staudinger Weg 5, 55128 Mainz, Germany.
* Correspondence: paul.pare@ttu.edu (P.W.P.); mohegazy@uni-mainz.de (M.E.F.H.); Tel.: +1-806-834-0461(P.W.P); Tel.: +2 033 371 635 (M.E.F.H

Supporting data	Page
S1: FTIR of **1** ……….……………………………………………...	4
S2: LRCIMS of **1** ………………………………………………....	5
S3: HRCIMS of **1** ………………………………………………....	6
S4: ^{1}H NMR of **1** ……..…………………………………………..	7
S5:^{13}C NMR of **1** ………………………………………………….	8
S6: DEPT 135 of **1** ……………………………………………....	9
S7: HSQC of **1** …………………………………………………..	10
S8: HMBC of **1**…………………………………………………	11
S9: ^{1}H ^{1}H COSY of **1**………….…………………………………	12
S10: NOESY of **1** ………………………………………………....	13
S11: FTIR of **2** ……….…………………………………………...	14
S12: LRCIMS of **2** ………………………………………………....	15
S13: HRCIMS of **2** ………………...……………………………...	16

S14: ^{1}H NMR of **2** ……………………………………………………..	17
S15:^{13}C NMR of **2** …………………………………………………	18
S16: DEPT 135 of **2** ………………………………………………...	19
S17: HSQC of **2** …………………………………………………..	20
S18: HMBC of **2**………………………………………………….	21
S19: ^{1}H ^{1}H COSY of **2**…………………………………………	22
S20: NOESY of **2** ………………………………………………...	23
S21: FTIR of **3** ………..……………………………………………...	24
S22: LRCIMS of **3** …………………………………………………...	25
S23: HRCIMS of **3** ..………………………………………………...	26
S24: ^{1}H NMR of **3** ……………………………………………………..	27
S25:^{13}C NMR of **3** ………..………………………………………….	28
S26: DEPT 135 of **3** ………………………………………………...	29
S27: HSQC of **3** …………………………………………………..	30
S28: HMBC of **3**..…………………………………………………….	31
S29: ^{1}H ^{1}H COSY of **3**…………………………………………	32
S30: NOESY of **3** ………………………………………………...	33
S31: FTIR of **4** ………..……………………………………………...	34
S32: LRCIMS of **4** …………………………………………………...	35
S33: HRCIMS of **4** ………………...………………………………...	36
S34: ^{1}H NMR of **4** ……………………………………………………..	37
S35:^{13}C NMR of **4** …………………………………………………	38
S36: DEPT 135 of **4** ………………………………………………...	39
S37: HSQC of **4** …………………………………………………..	40
S38: HMBC of **4**………………………………………………….	41
S39: ^{1}H ^{1}H COSY of **4**…………………………………………	42
S40: NOESY of **4** ………………………………………………...	43
S41: FTIR of **5** ………..……………………………………………...	44
S42: LRCIMS of **5** …………………………………………………...	45
S43: HRCIMS of **5** ………………...………………………………...	46
S44: ^{1}H NMR of **5** ……………………………………………………..	47
S45:^{13}C NMR of **5** …………………………………………………	48

S46: DEPT 135 of **5** ……………………………………………………...	49
S47: HSQC of **5** …………………………………………………………..	50
S48: HMBC of **5**………………………………………………………….	51
S49: ^{1}H ^{1}H COSY of **5**………………….……………………………………	52
S50: NOESY of **5** ……………………………………………………....	53

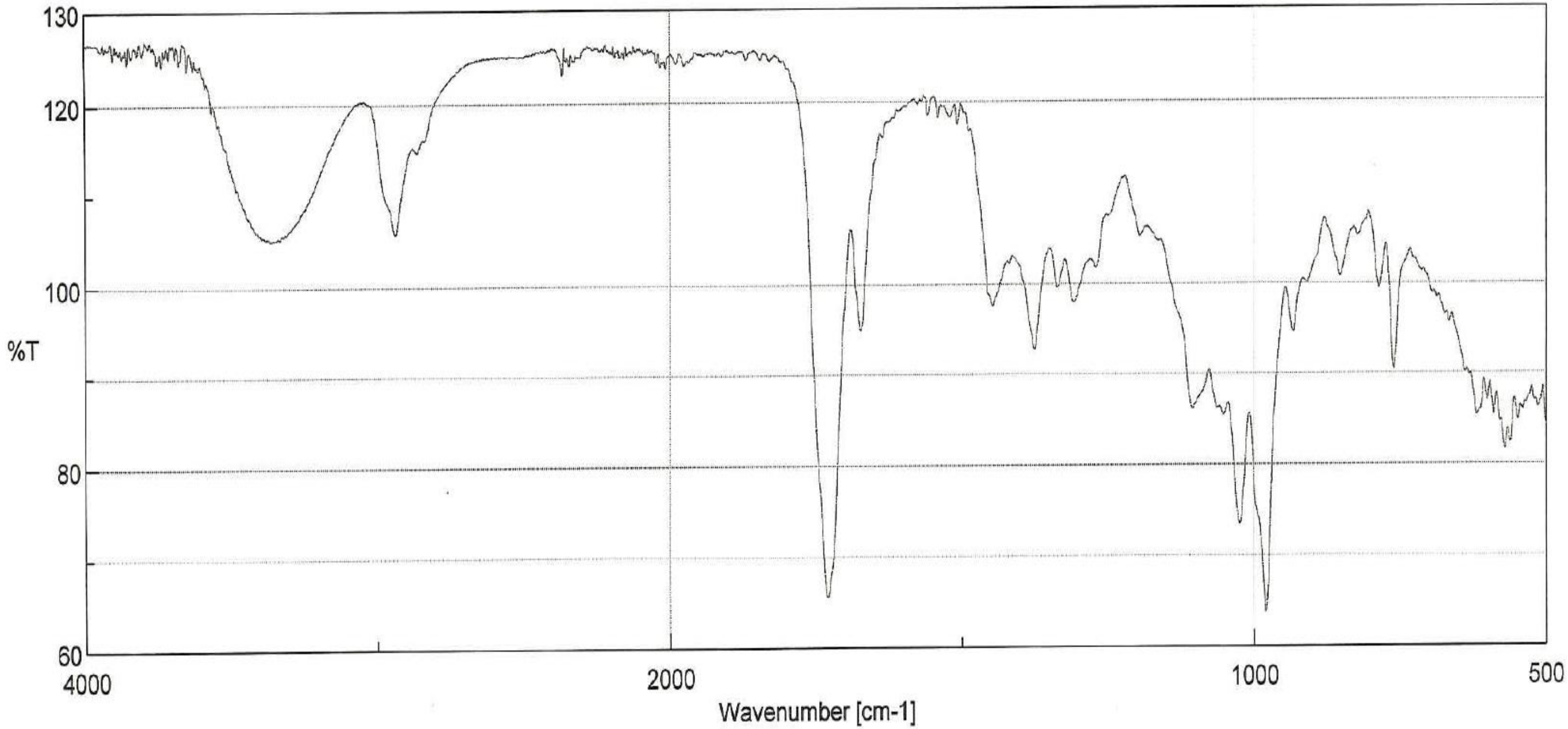

S1: FTIR of **1**

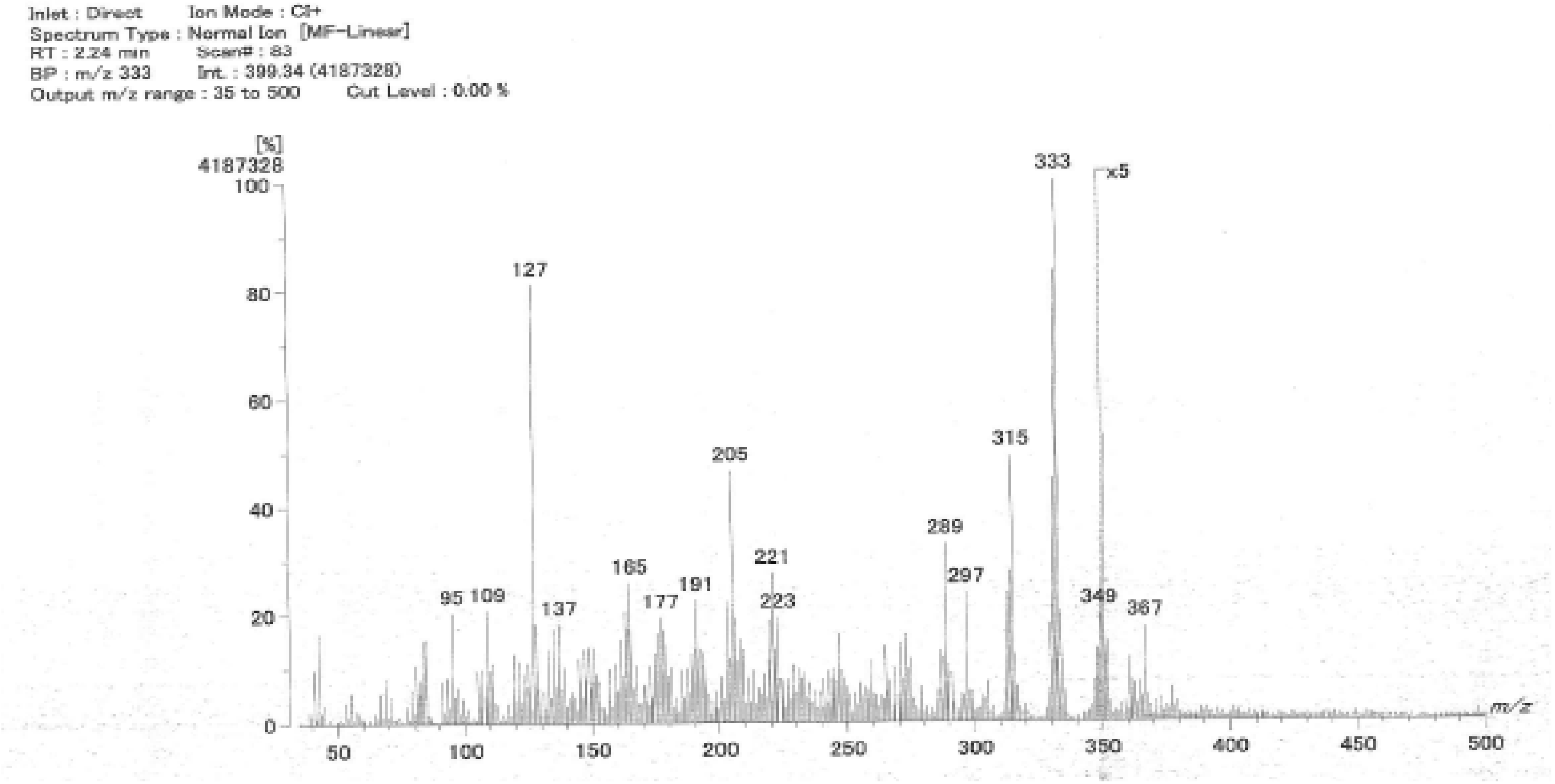

S2: LRCIMS of **1**

Note : MStation
Inlet : Direct   Ion Mode : CI+
RT : 1.92 min   Scan# : 49
Elements : C 150/0, H 250/0, O 50/0
Mass Tolerance   : 5mmu
Unsaturation (U.S.) : 0.0 – 15.0

	Observed m/z	Int%	Err[ppm / mmu]	U.S.	Composition
1	367.2122	4.78	+0.4 / +0.1	5.5	C20 H31 O6

S3: HRCIMS of **1**

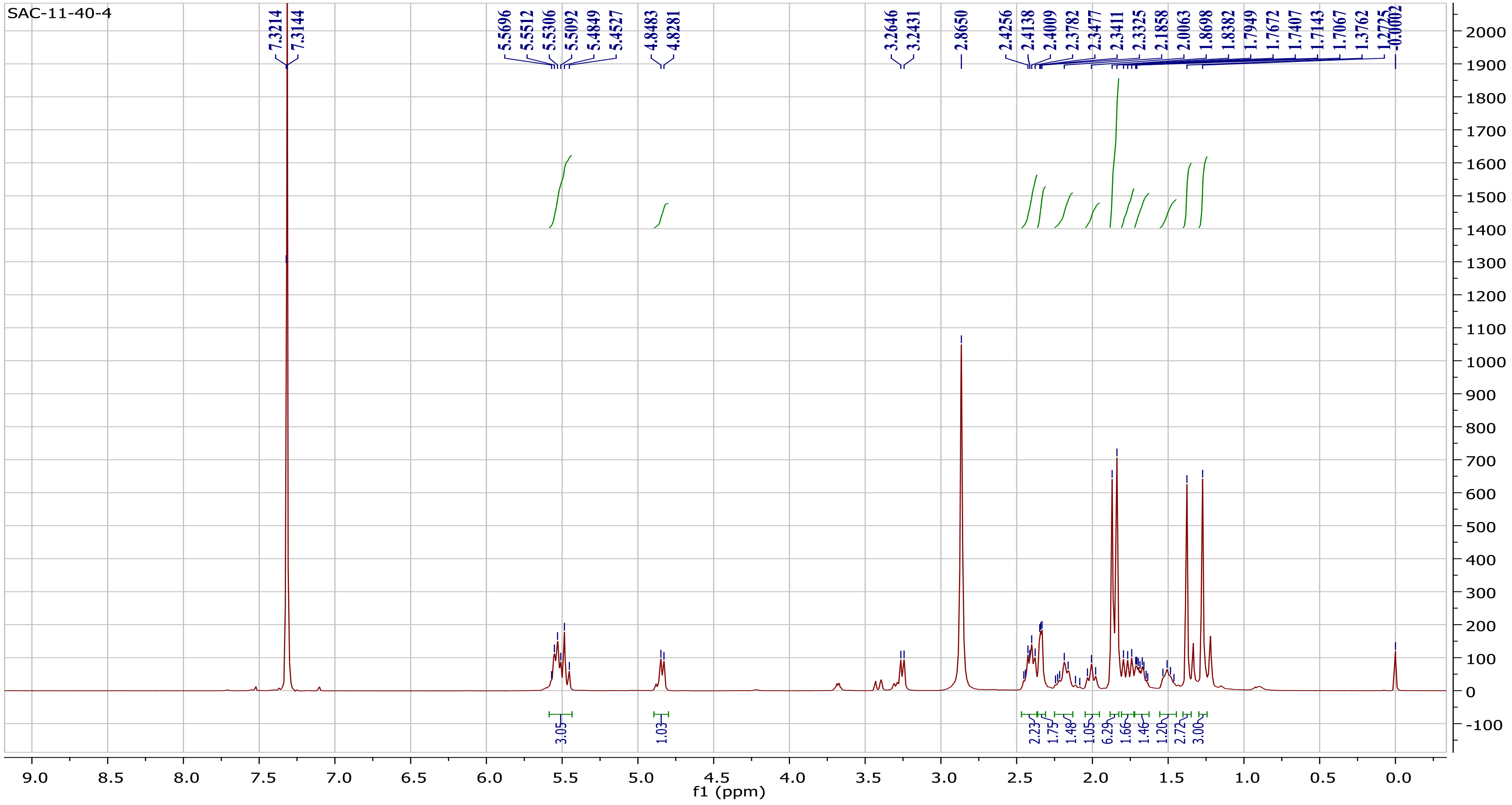

S4: ^{1}H NMR of **1** (500 Hz, $CDCl_3$)

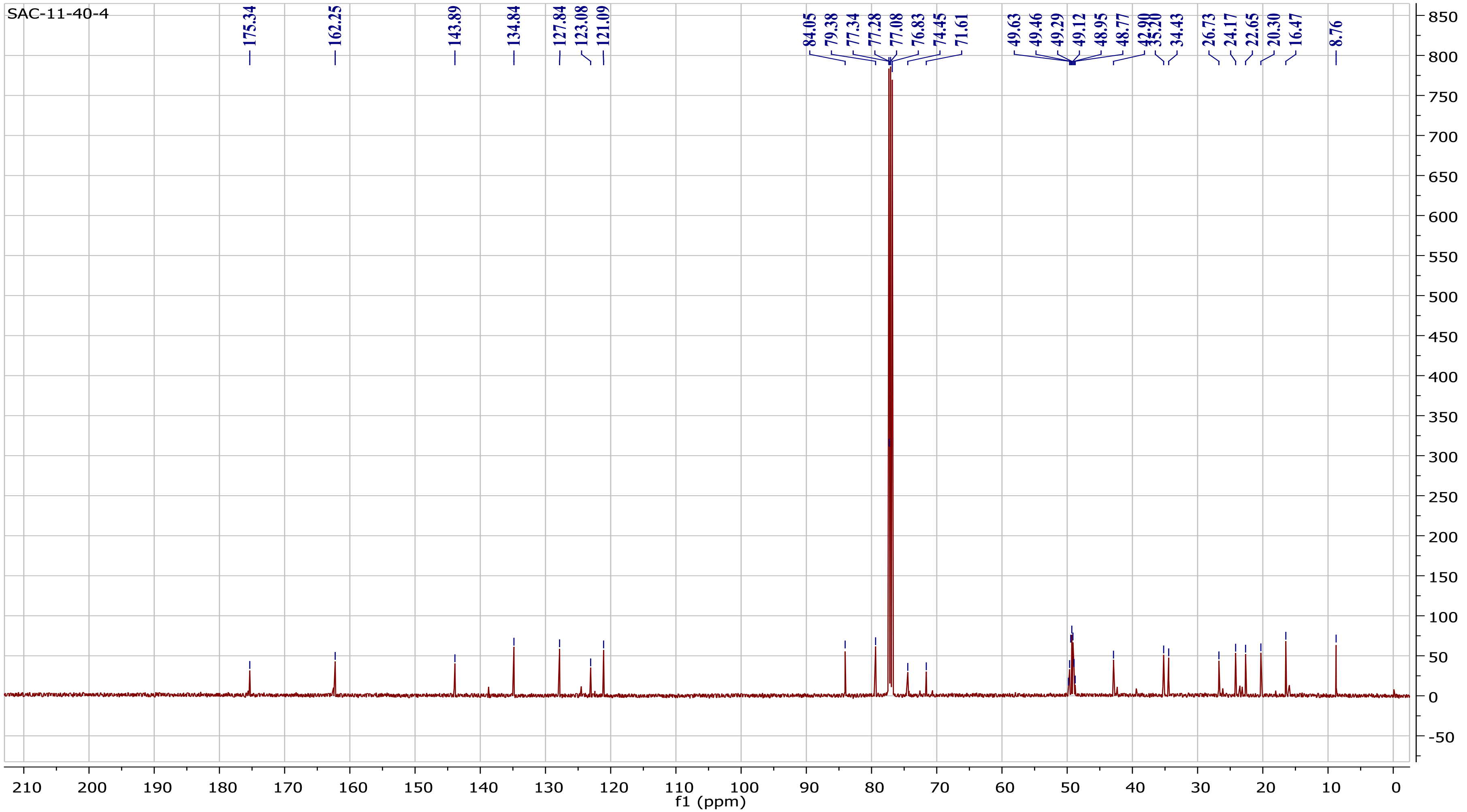

S5: ^{13}C NMR of **1** (125 Hz, $CDCl_3$)

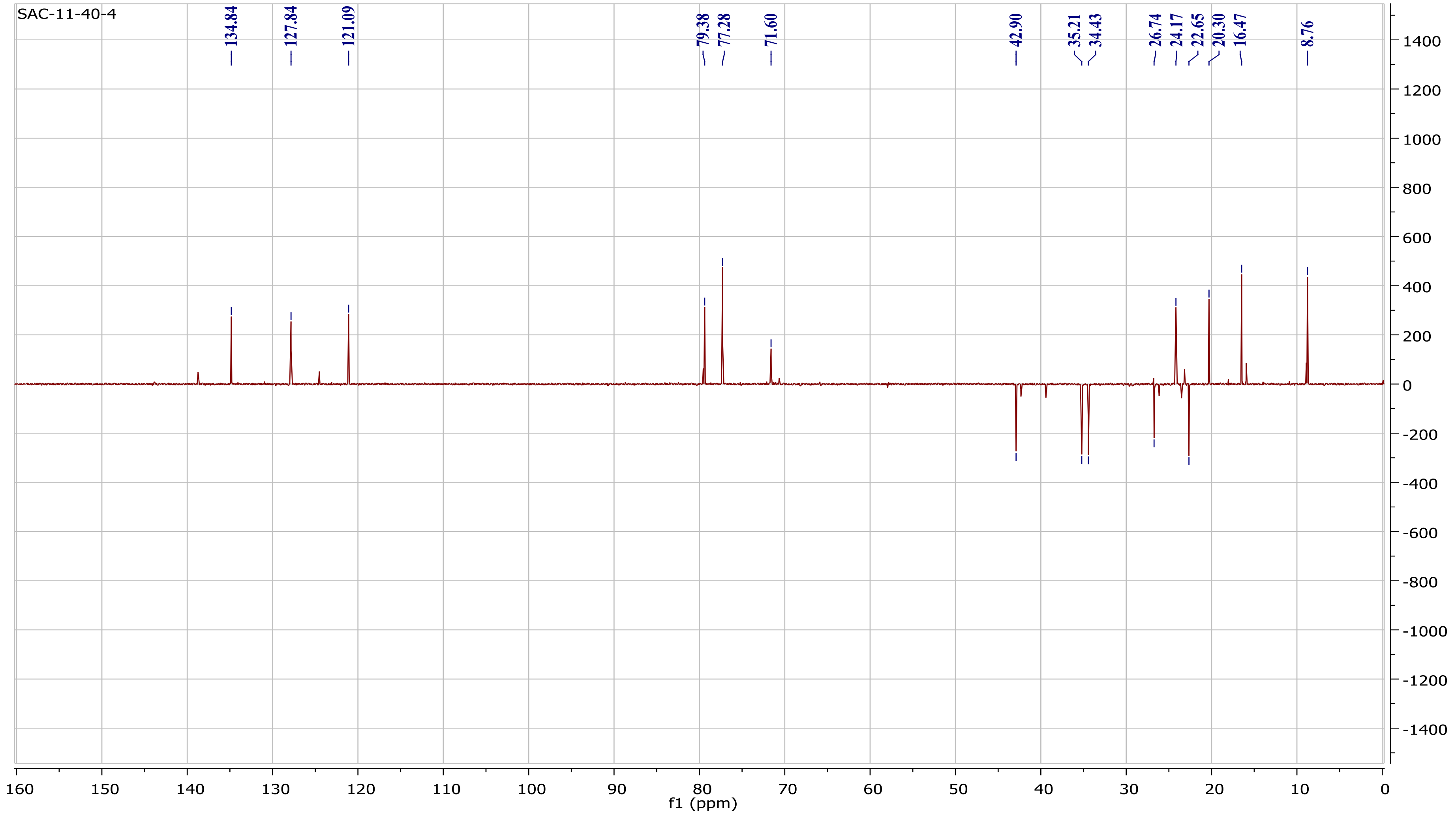
SAC-11-40-4
134.84
127.84
121.09
79.38
77.28
71.60
42.90
35.21
34.43
26.74
24.17
22.65
20.30
16.47
8.76
f1 (ppm)

S6: DEPT of **1**

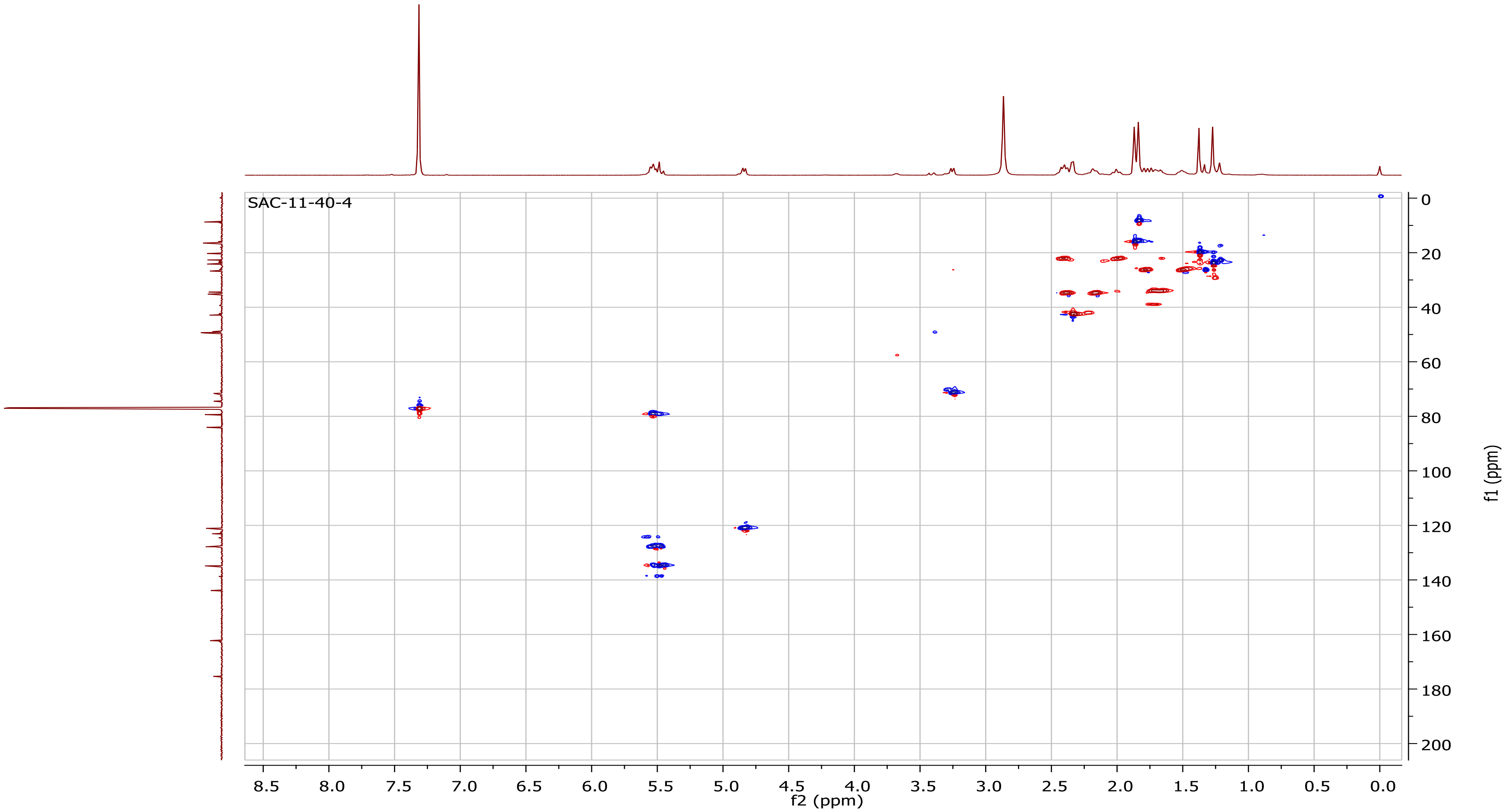
SAC-11-40-4
f2 (ppm)
f1 (ppm)
8.5
8.0
7.5
7.0
6.5
6.0
5.5
5.0
4.5
4.0
3.5
3.0
2.5
2.0
1.5
1.0
0.5
0.0
0
20
40
60
80
100
120
140
160
180
200

S7: HSQC of **1**

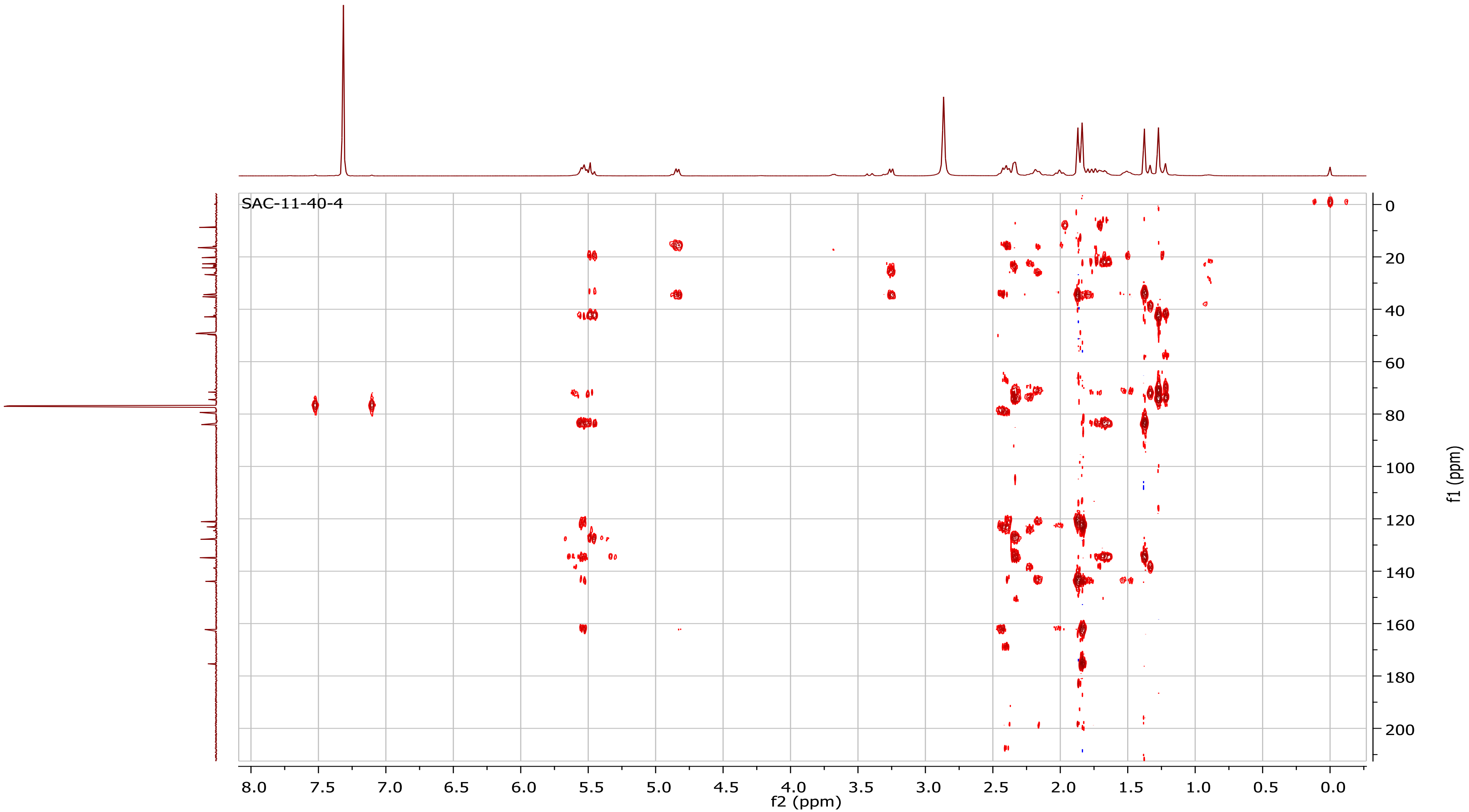

SAC-11-40-4
8.0 7.5 7.0 6.5 6.0 5.5 5.0 4.5 4.0 3.5 3.0 2.5 2.0 1.5 1.0 0.5 0.0
f2 (ppm)
0 20 40 60 80 100 120 140 160 180 200
f1 (ppm)

S8: HMBC of **1**

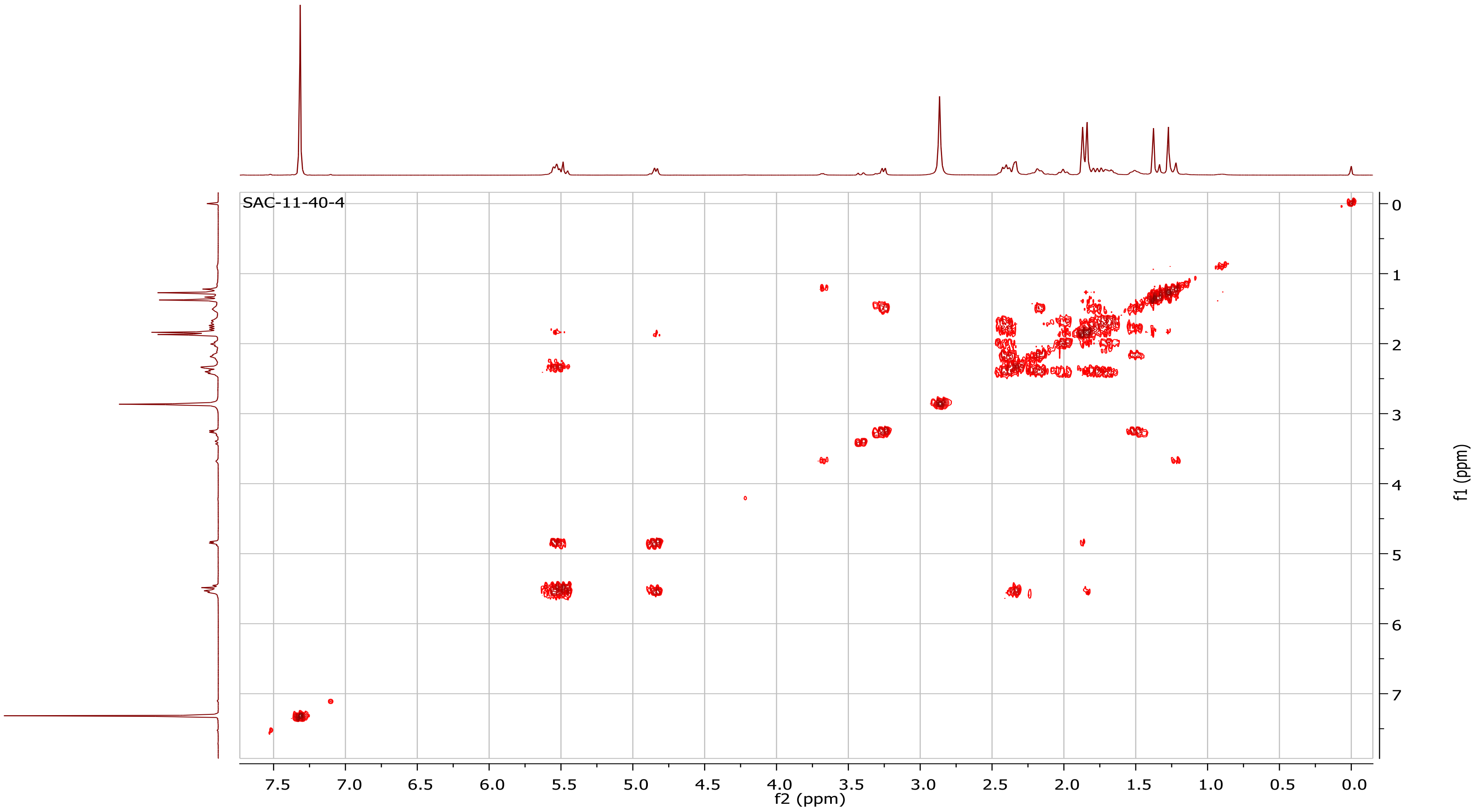

S9: ^{1}H ^{1}H COSY of 1

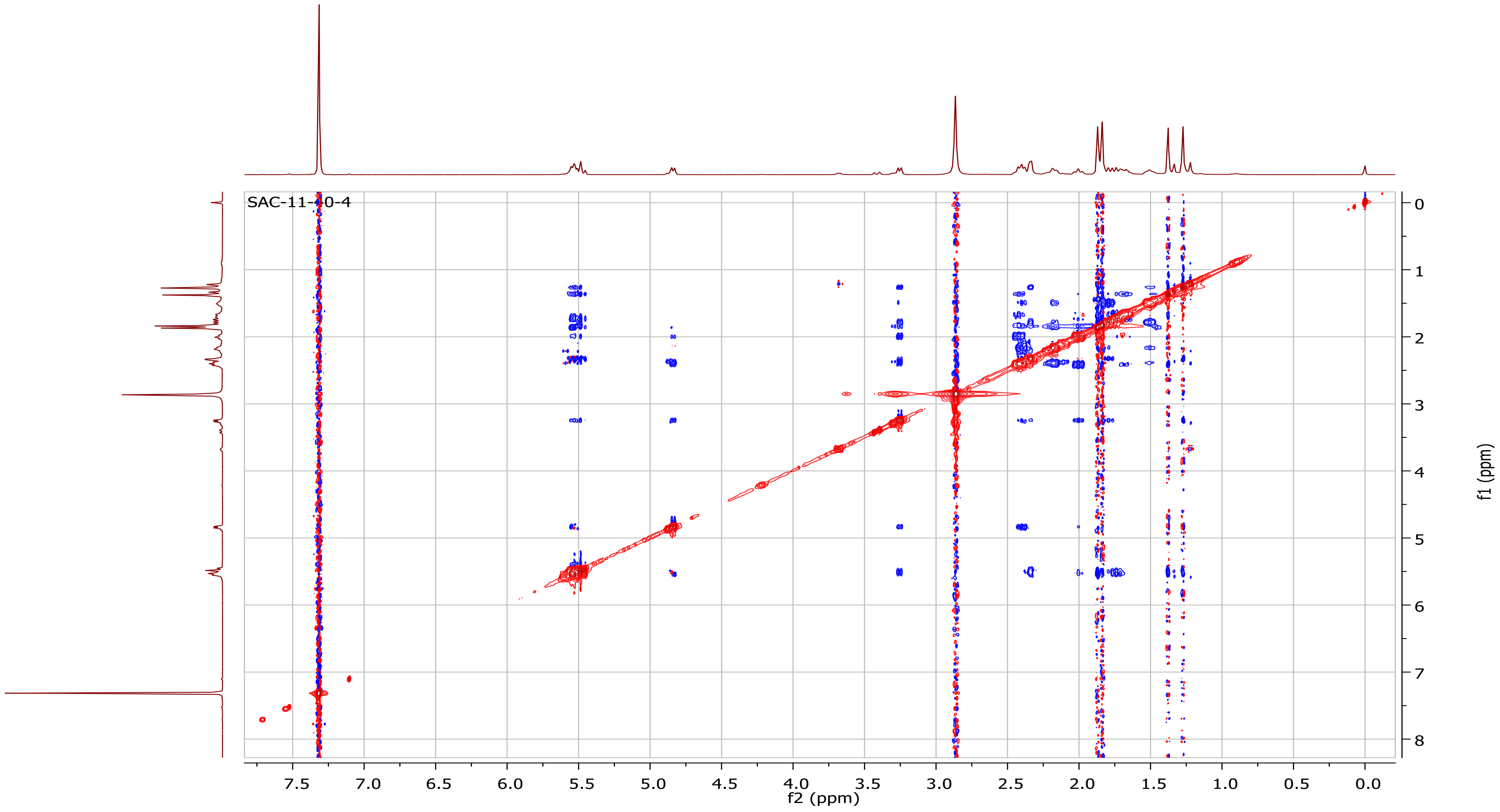

S10: NOESY of **1**

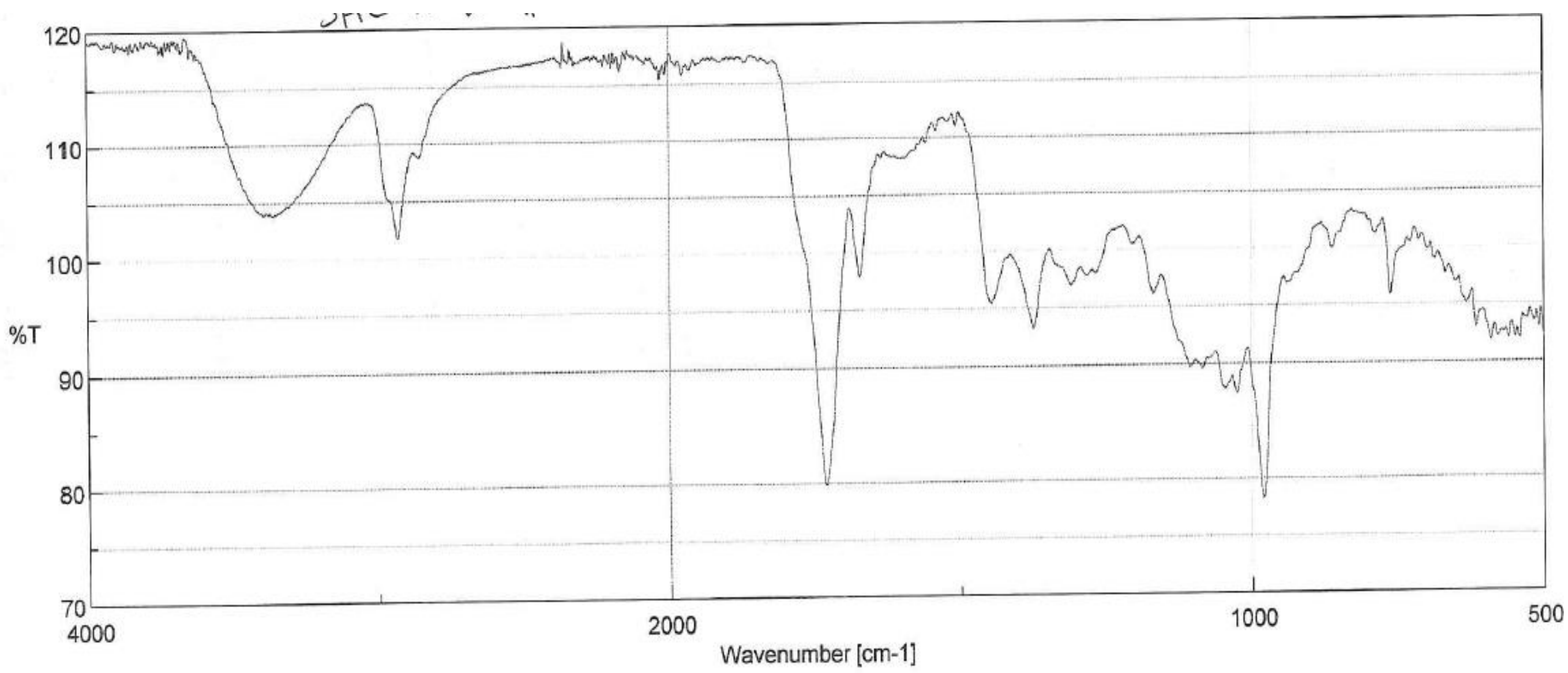

S11: FTIR of **2**

Inlet : Direct Ion Mode : CI+
Spectrum Type : Normal Ion [MF-Linear]
RT : 1.78 min Scan# : 66
BP : m/z 127 Int. : 304.82 (3196288)
Output m/z range : 35 to 500 Cut Level : 0.00 %

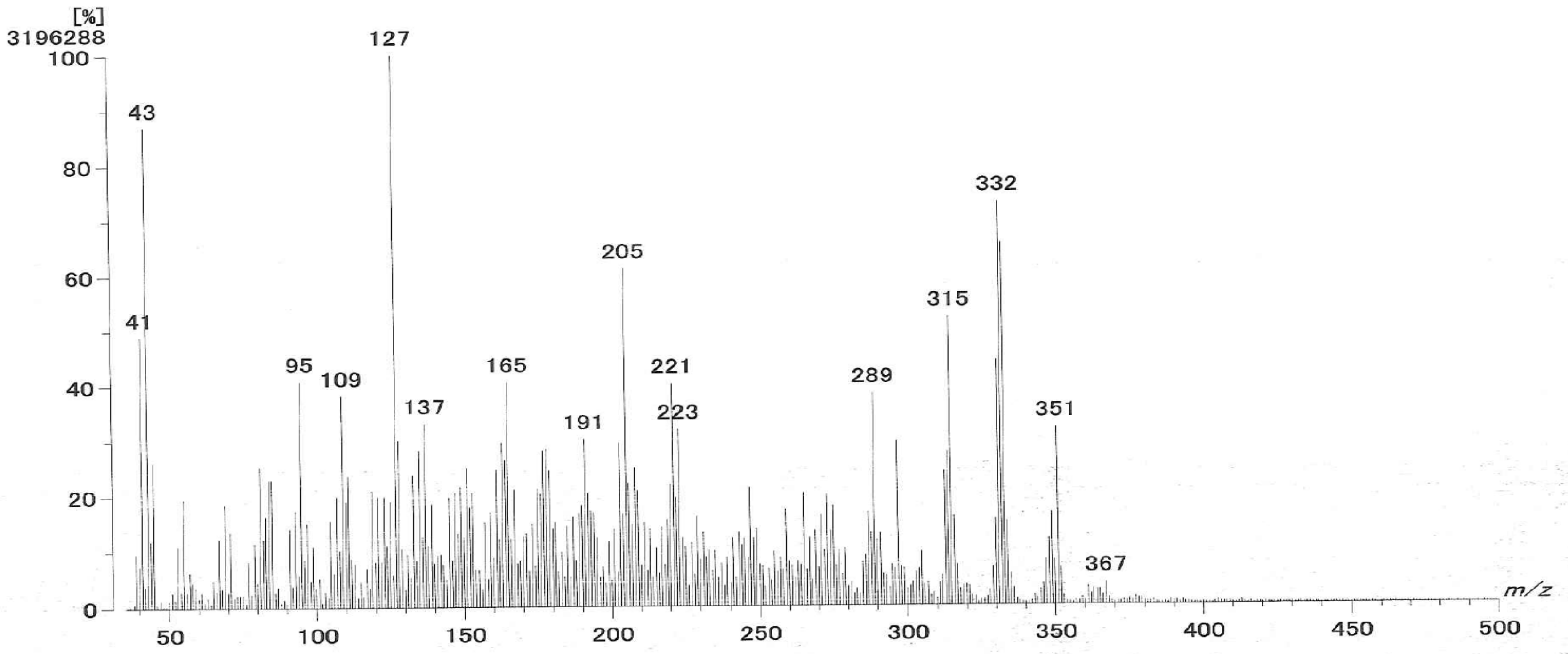

S12: LRCIMS of **2**

Note : MStation
Inlet : Direct Ion Mode : CI+
RT : 1.92 min Scan# : 49
Elements : C 150/0, H 250/0, O 50/0
Mass Tolerance : 5mmu
Unsaturation (U.S.) : 0.0 – 15.0

	Observed m/z	Int%	Err[ppm / mmu]	U.S.	Composition
1	367.2122	4.78	+0.4 / +0.1	5.5	C20 H31 O6

S13: HRCIMS of **2**

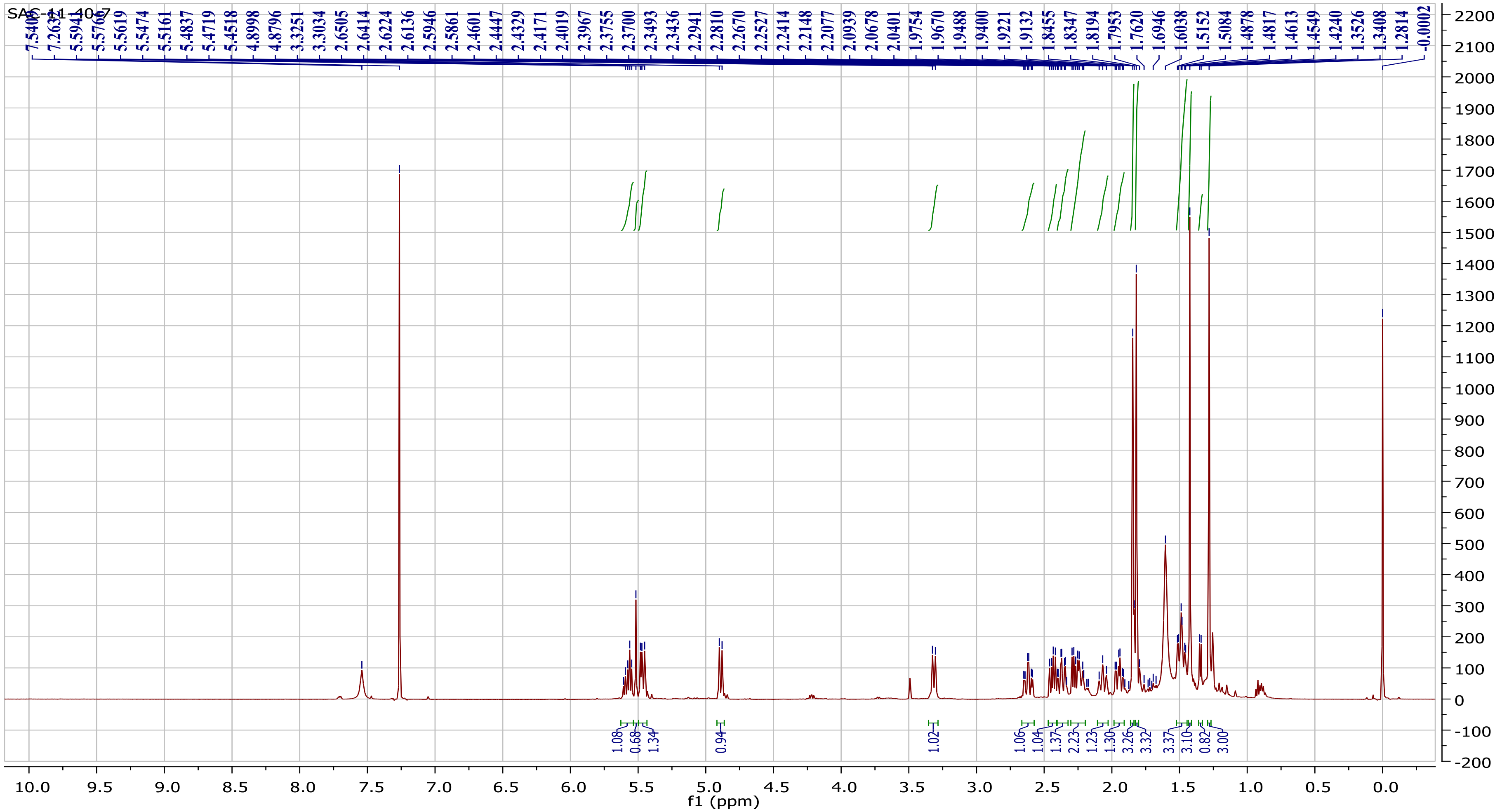

S14: ^{1}H NMR of **2**

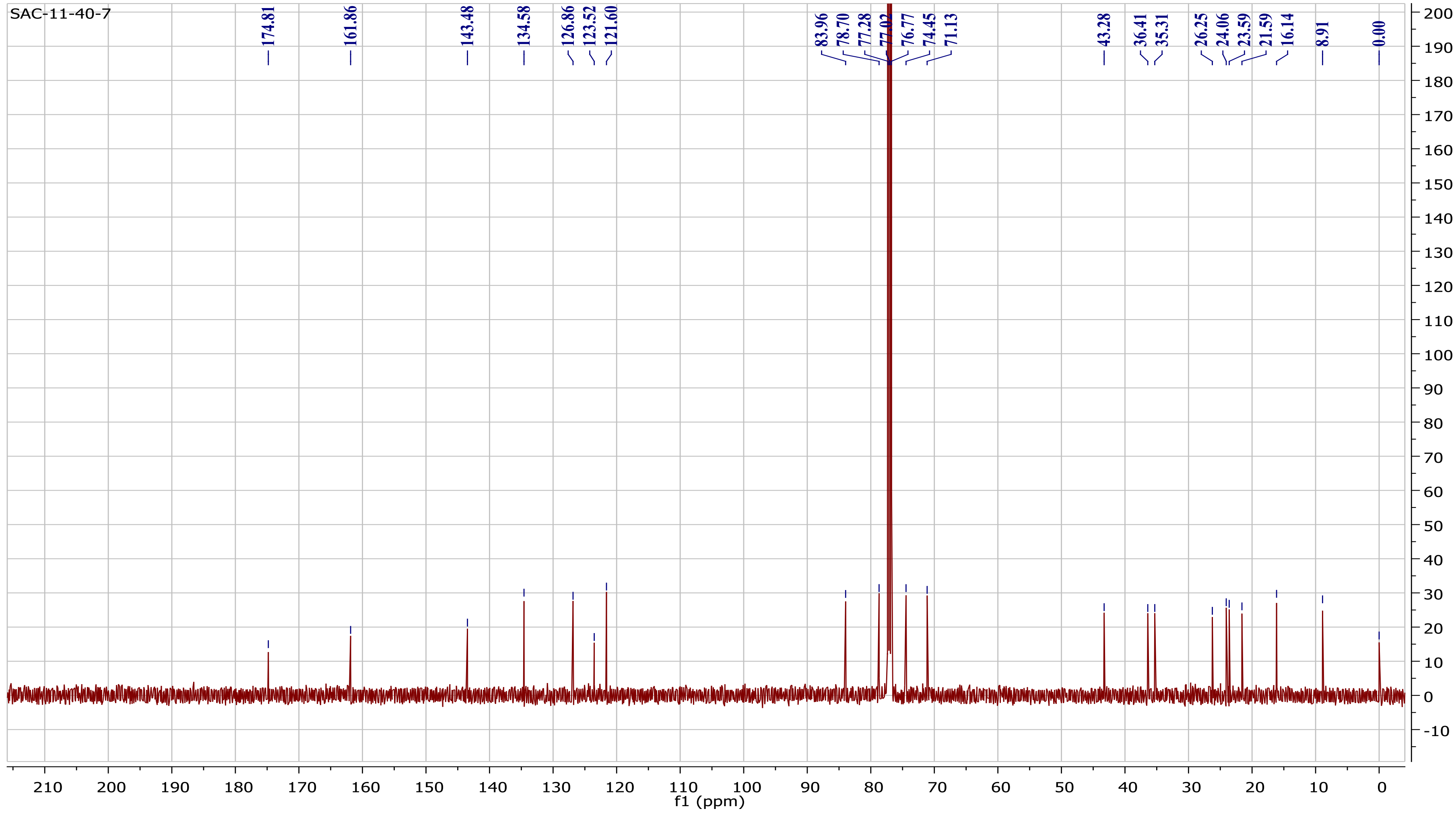

S15: ^{13}C NMR of **2**

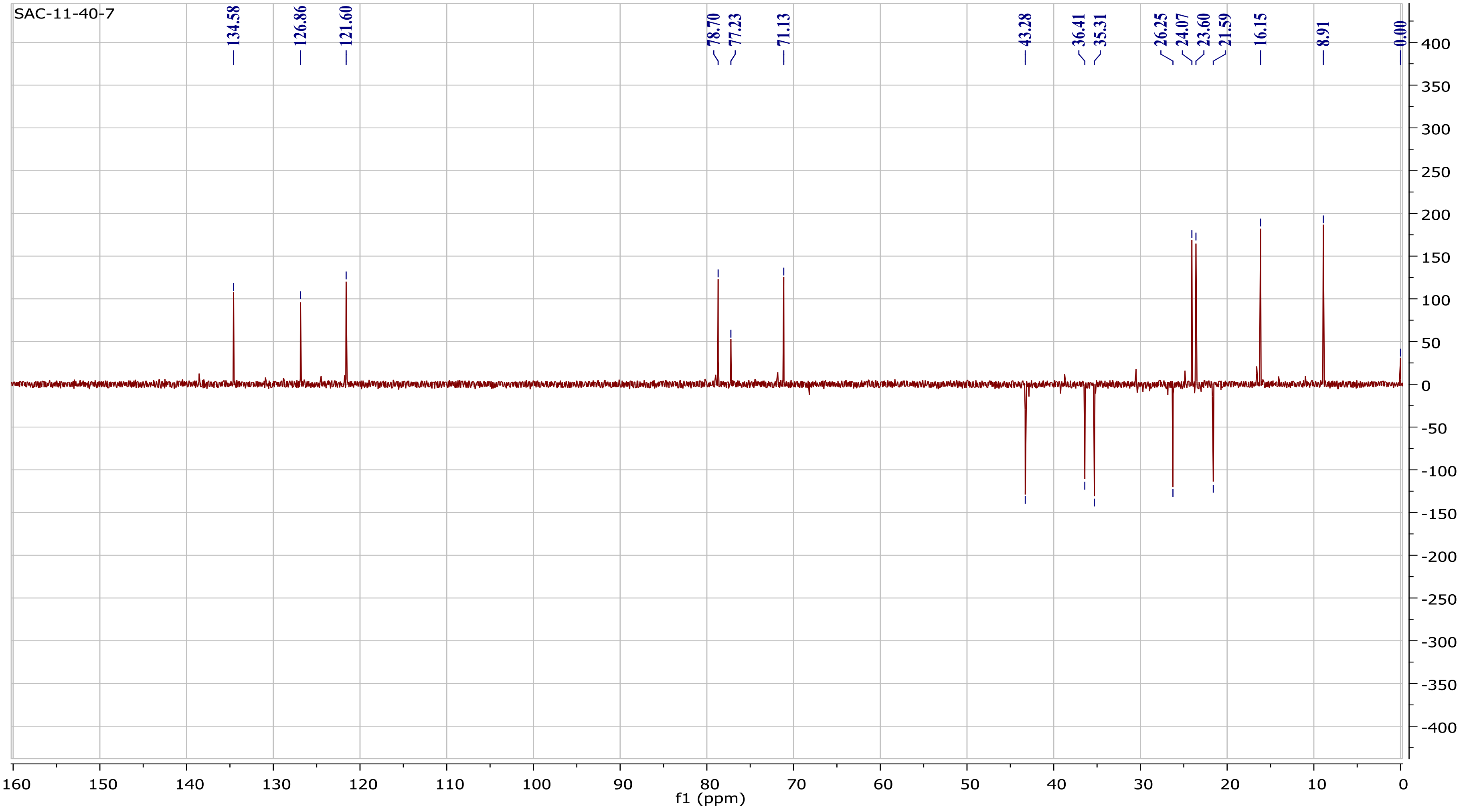
SAC-11-40-7
134.58
126.86
121.60
78.70
77.23
71.13
43.28
36.41
35.31
26.25
24.07
23.60
21.59
16.15
8.91
0.00
f1 (ppm)

S16: DEPT of **2**

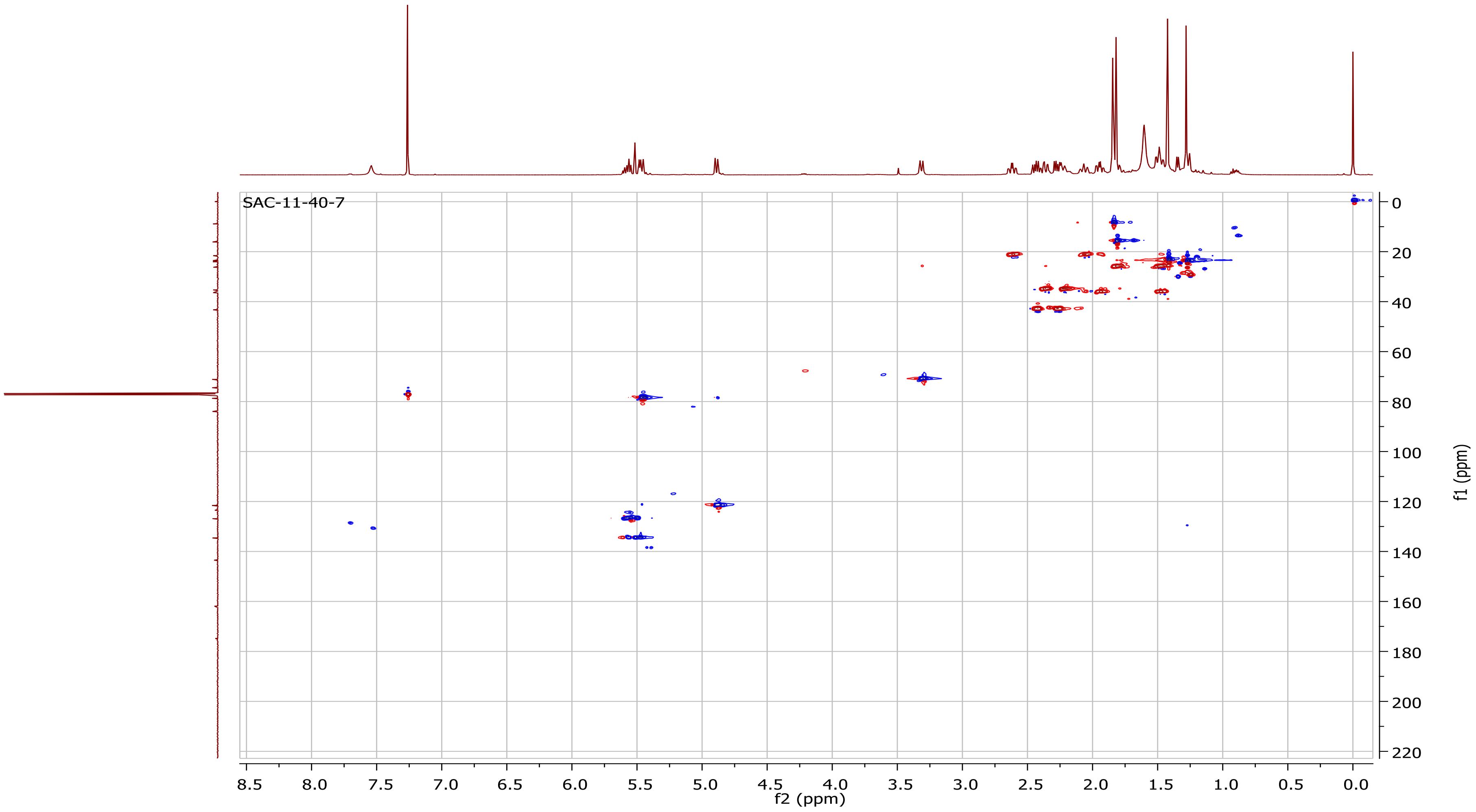

SAC-11-40-7
8.5
8.0
7.5
7.0
6.5
6.0
5.5
5.0
4.5
4.0
3.5
3.0
2.5
2.0
1.5
1.0
0.5
0.0
f2 (ppm)
0
20
40
60
80
100
120
140
160
180
200
220
f1 (ppm)

S17: HSQC of **2**

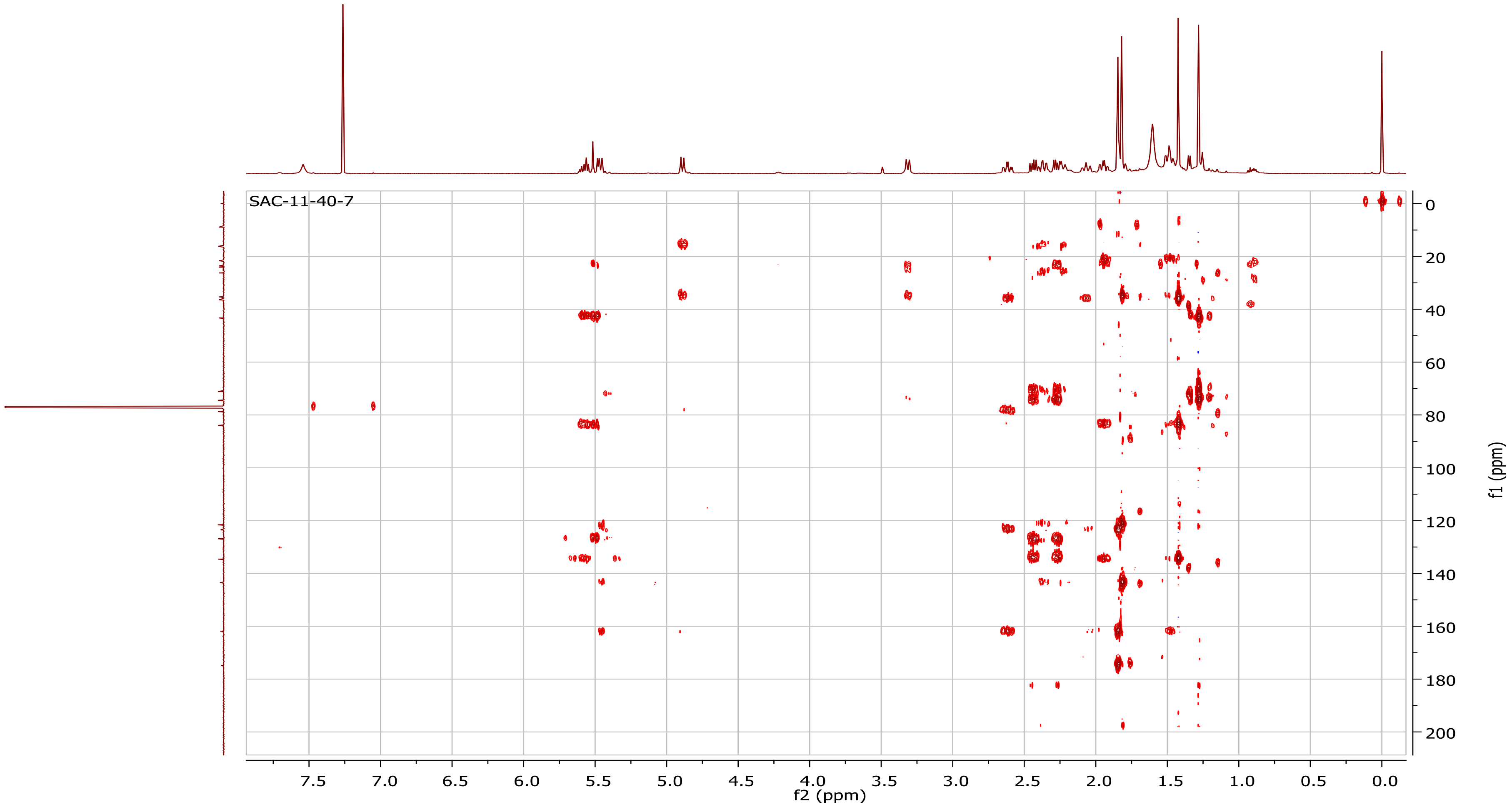

SAC-11-40-7
0
20
40
60
80
100
120
140
160
180
200
f1 (ppm)
7.5
7.0
6.5
6.0
5.5
5.0
4.5
4.0
3.5
3.0
2.5
2.0
1.5
1.0
0.5
0.0
f2 (ppm)

S18: HMBC of **2**

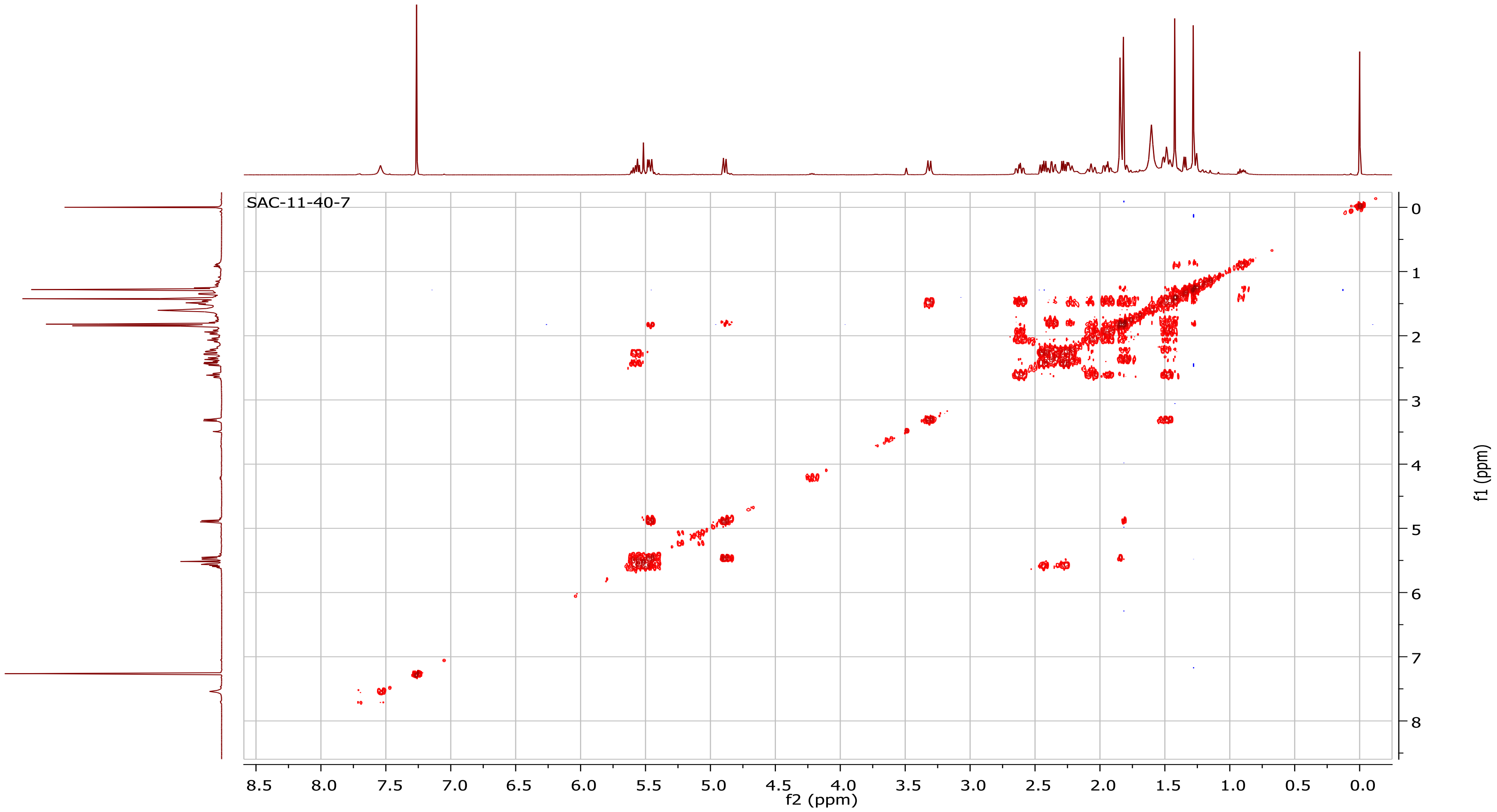

S19: ^{1}H ^{1}H COSY of **2**

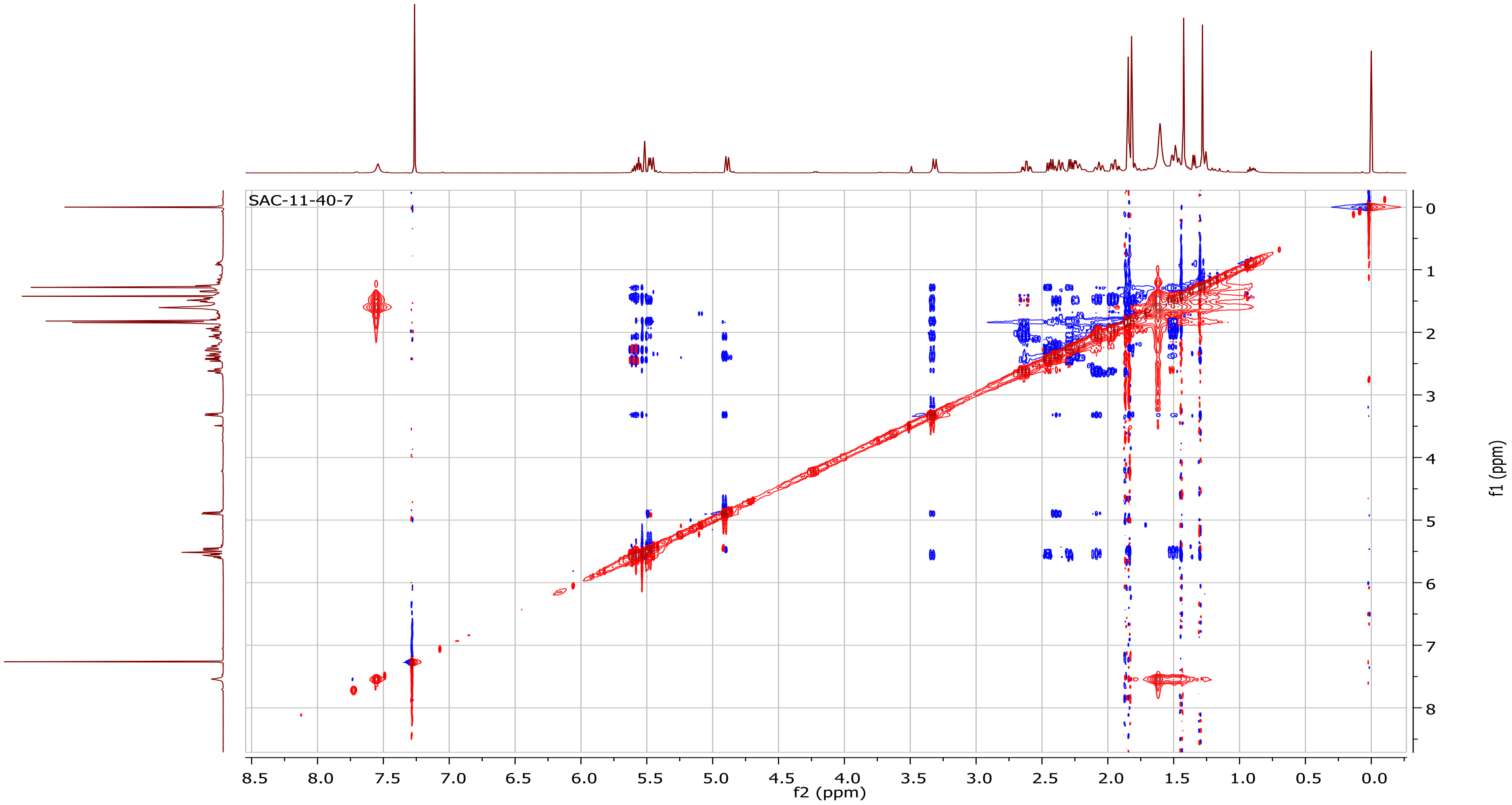

S20: NOESY of **2**

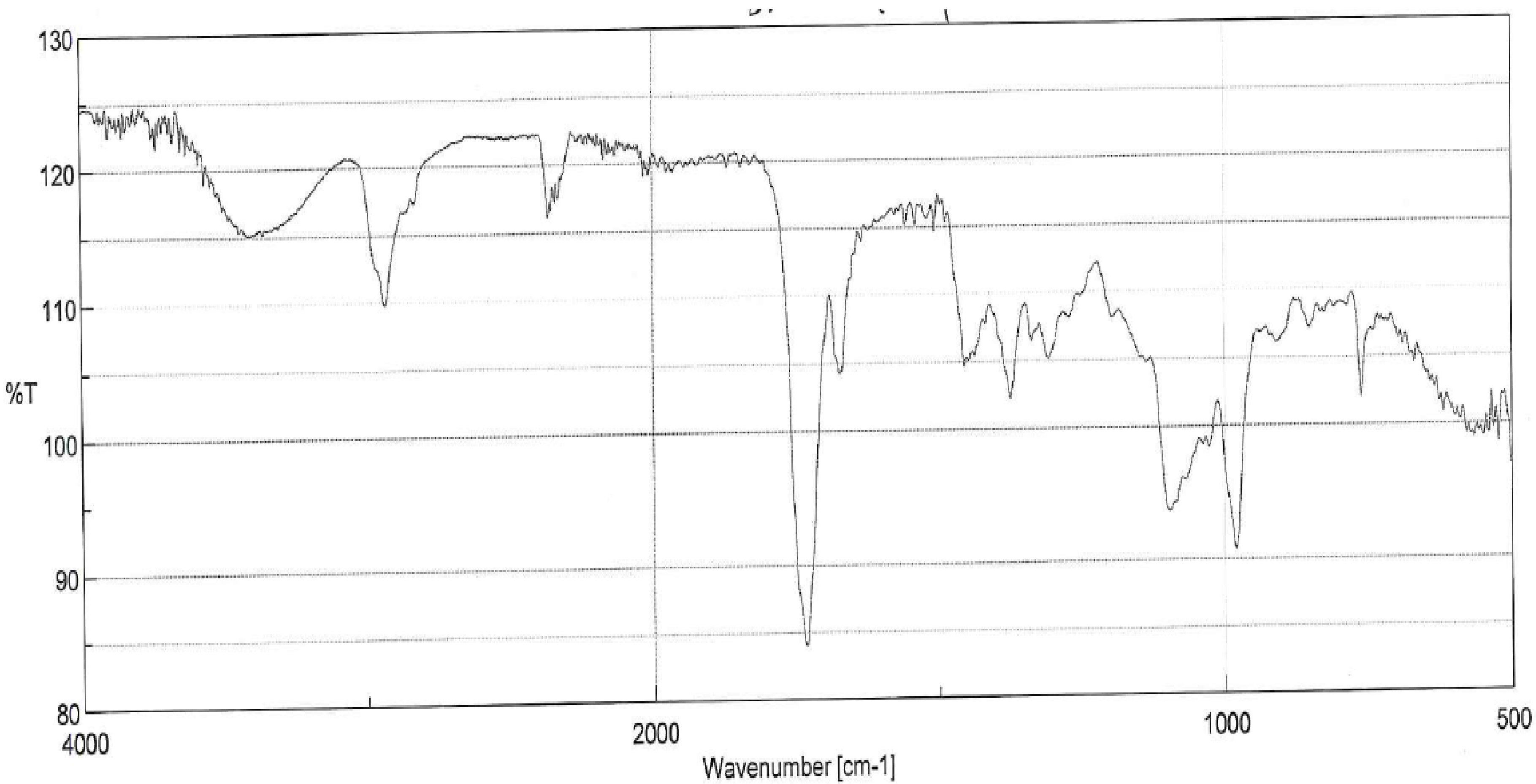

S21: LRCIMS of **3**

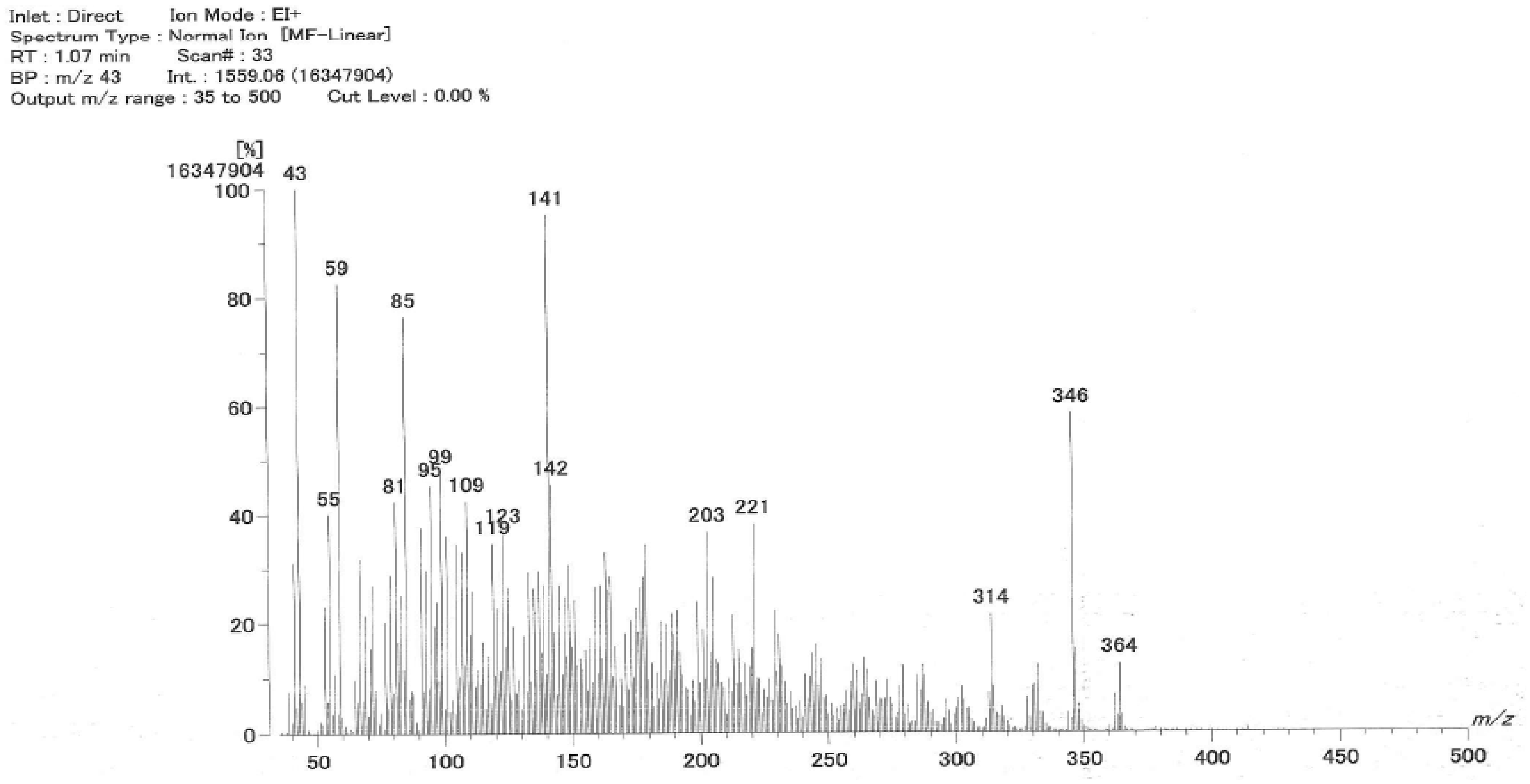

S22: LREIMS of **3**

Inlet : Direct   Ion Mode : EI+
RT : 1.48 min   Scan# : 38
Elements : C 150/0, H 250/0, O 50/0
Mass Tolerance : 5mmu
Unsaturation (U.S.) : 0.0 – 20.0

	Observed m/z	Int%	Err[ppm / mmu]	U.S.	Composition
1	364.2258	21.08	+2.3 / +0.8	6.0	C21 H32 O5

S23: HREIMS of **3**

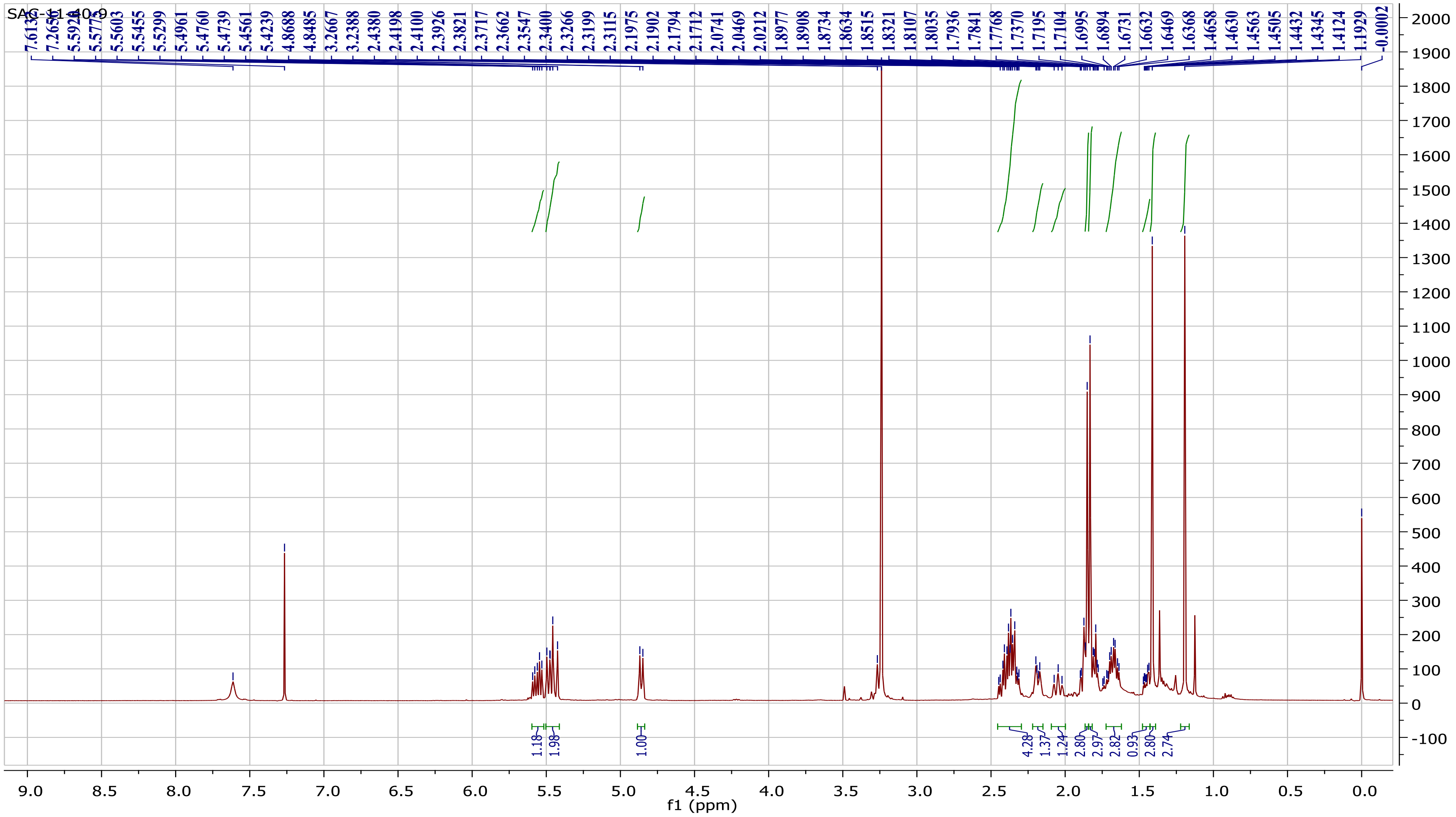

S24: ^{1}H NMR of **3**

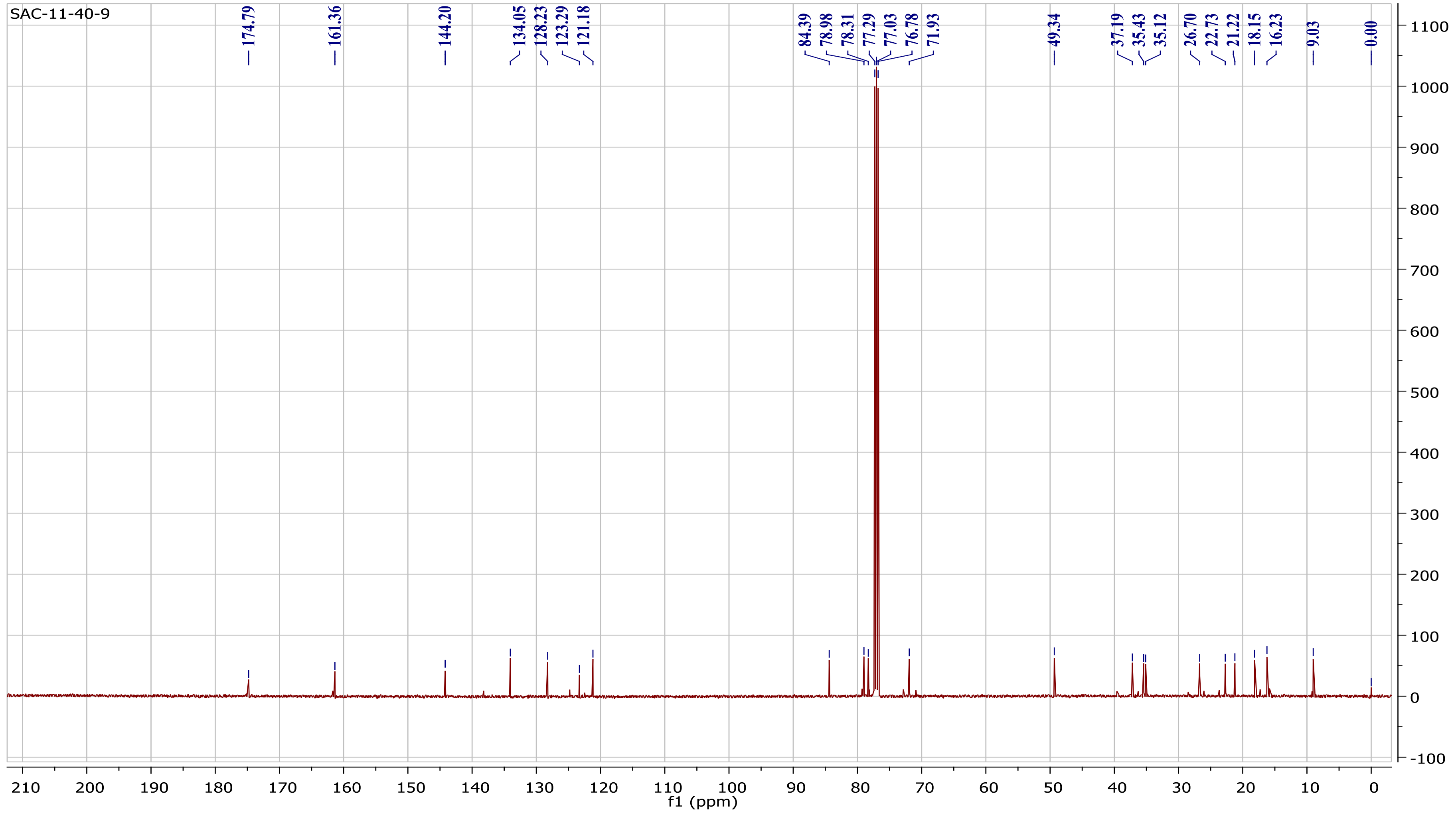

S25: ^{13}C NMR of **3**

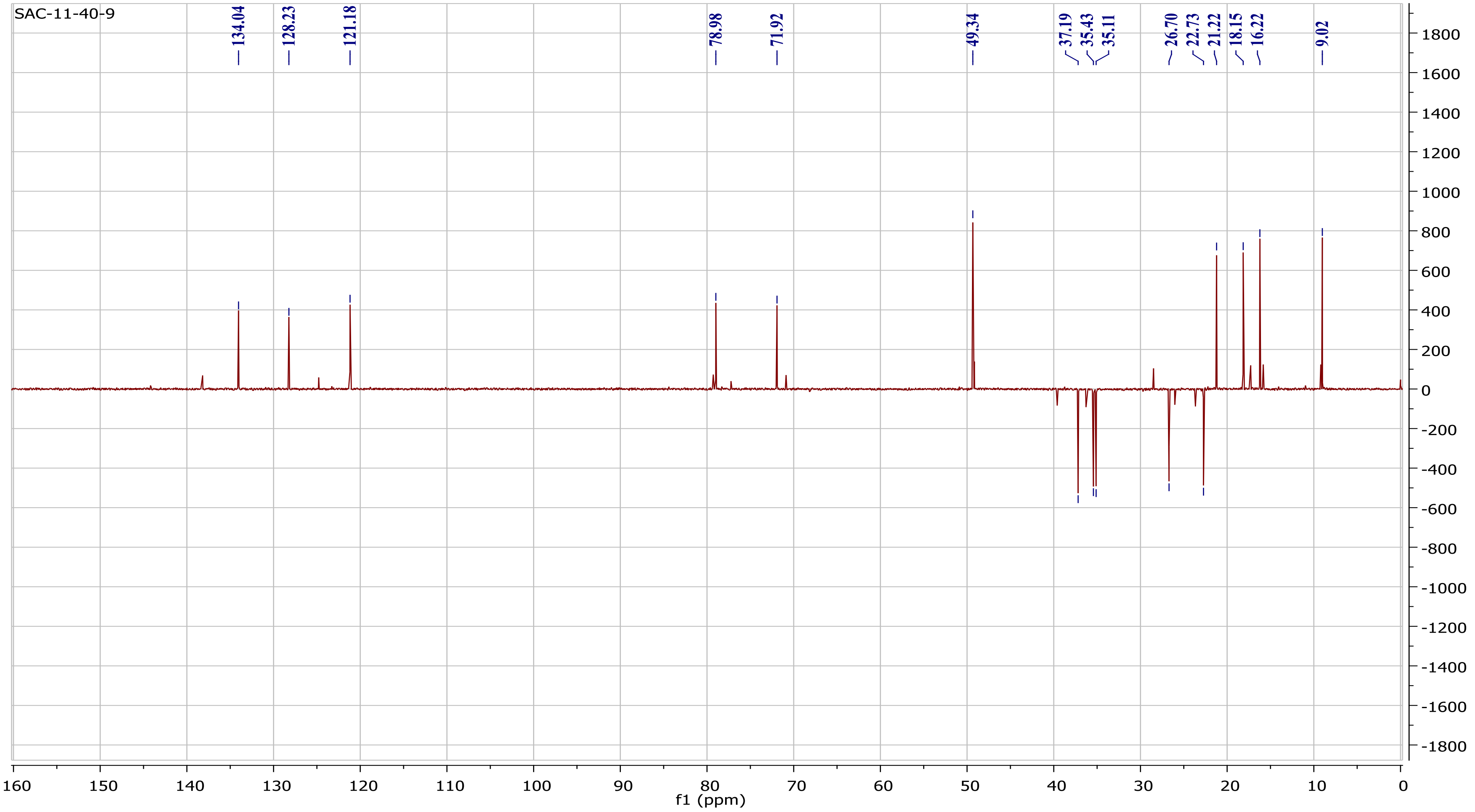

SAC-11-40-9
134.04
128.23
121.18
78.98
71.92
49.34
37.19
35.43
35.11
26.70
22.73
21.22
18.15
16.22
9.02
f1 (ppm)

S26: DEPT of **3**

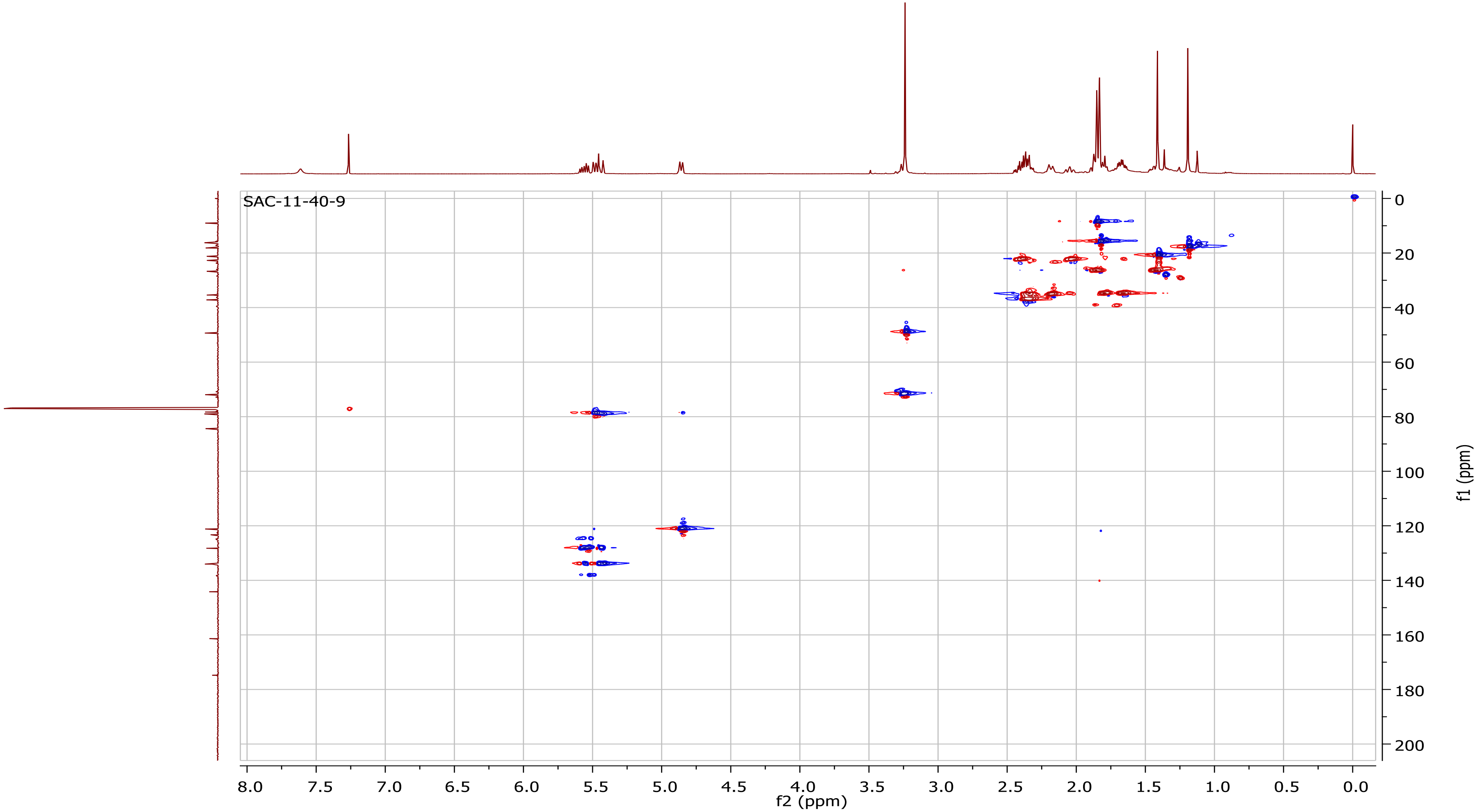
SAC-11-40-9
f2 (ppm)
f1 (ppm)
8.0
7.5
7.0
6.5
6.0
5.5
5.0
4.5
4.0
3.5
3.0
2.5
2.0
1.5
1.0
0.5
0.0
0
20
40
60
80
100
120
140
160
180
200

S27: HSQC of **3**

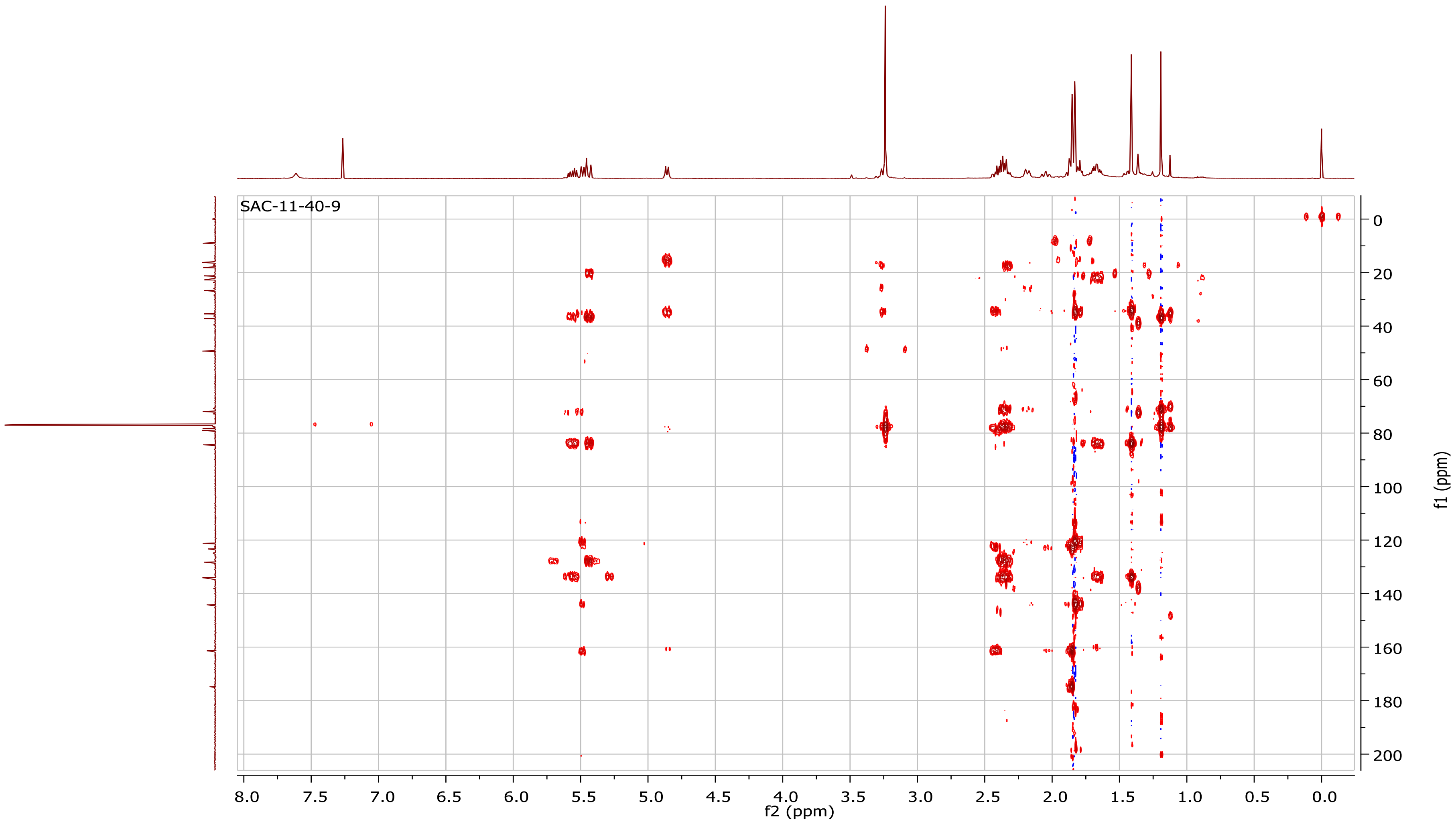
SAC-11-40-9
8.0
7.5
7.0
6.5
6.0
5.5
5.0
4.5
4.0
3.5
3.0
2.5
2.0
1.5
1.0
0.5
0.0
f2 (ppm)
0
20
40
60
80
100
120
140
160
180
200
f1 (ppm)

S28: HMBC of **3**

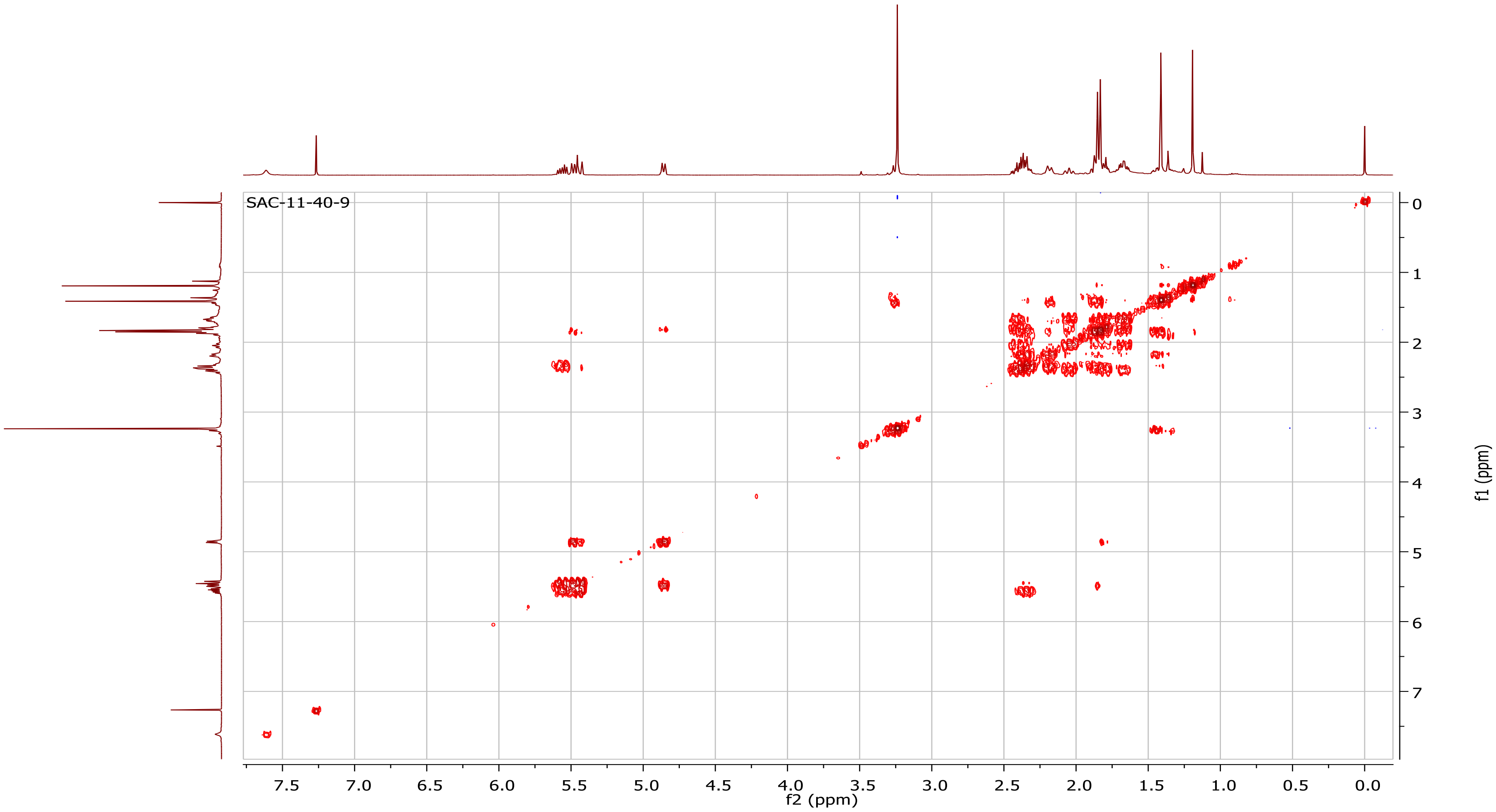

S29: ^{1}H ^{1}H COSY of **3**

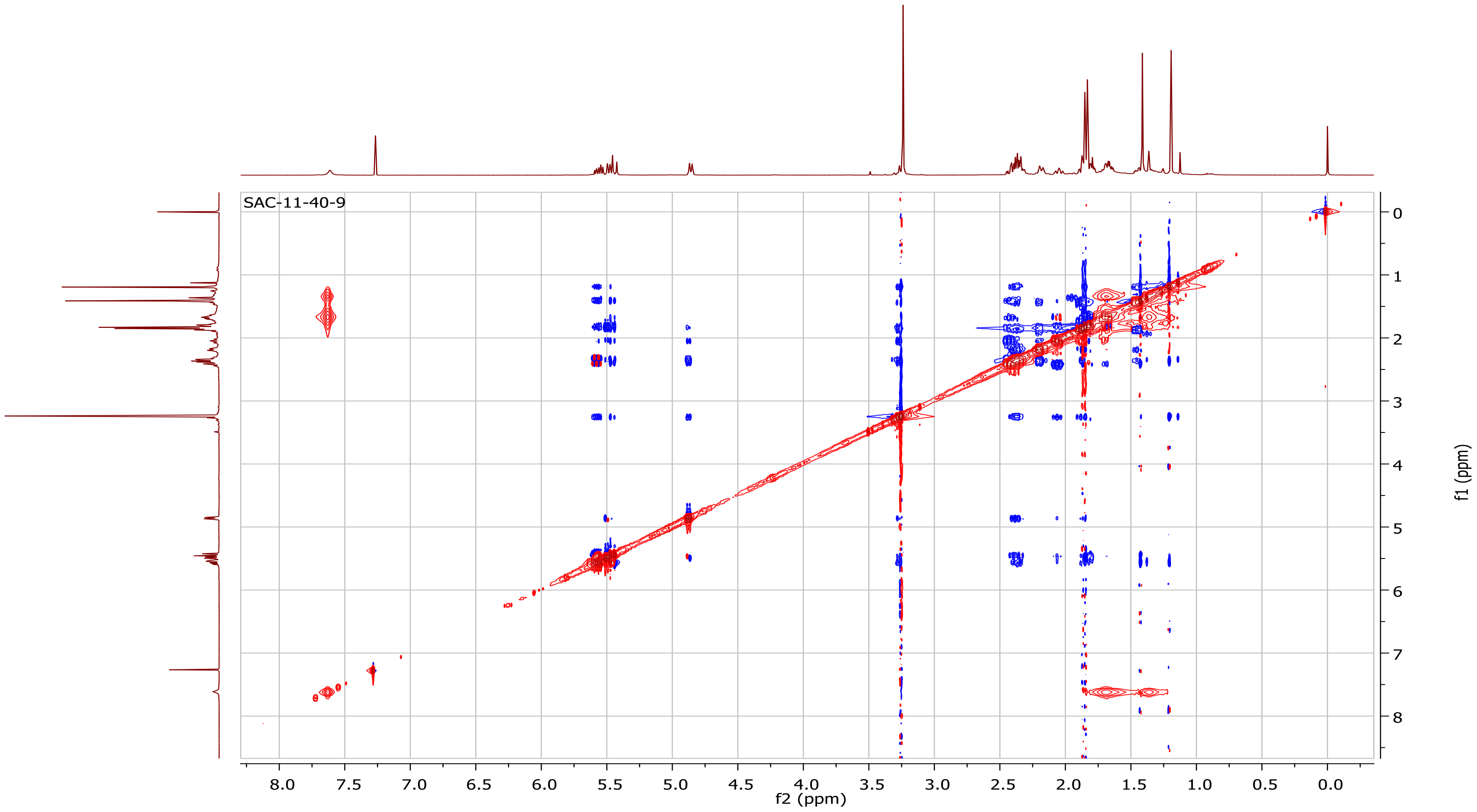

S30: NOESY of **3**

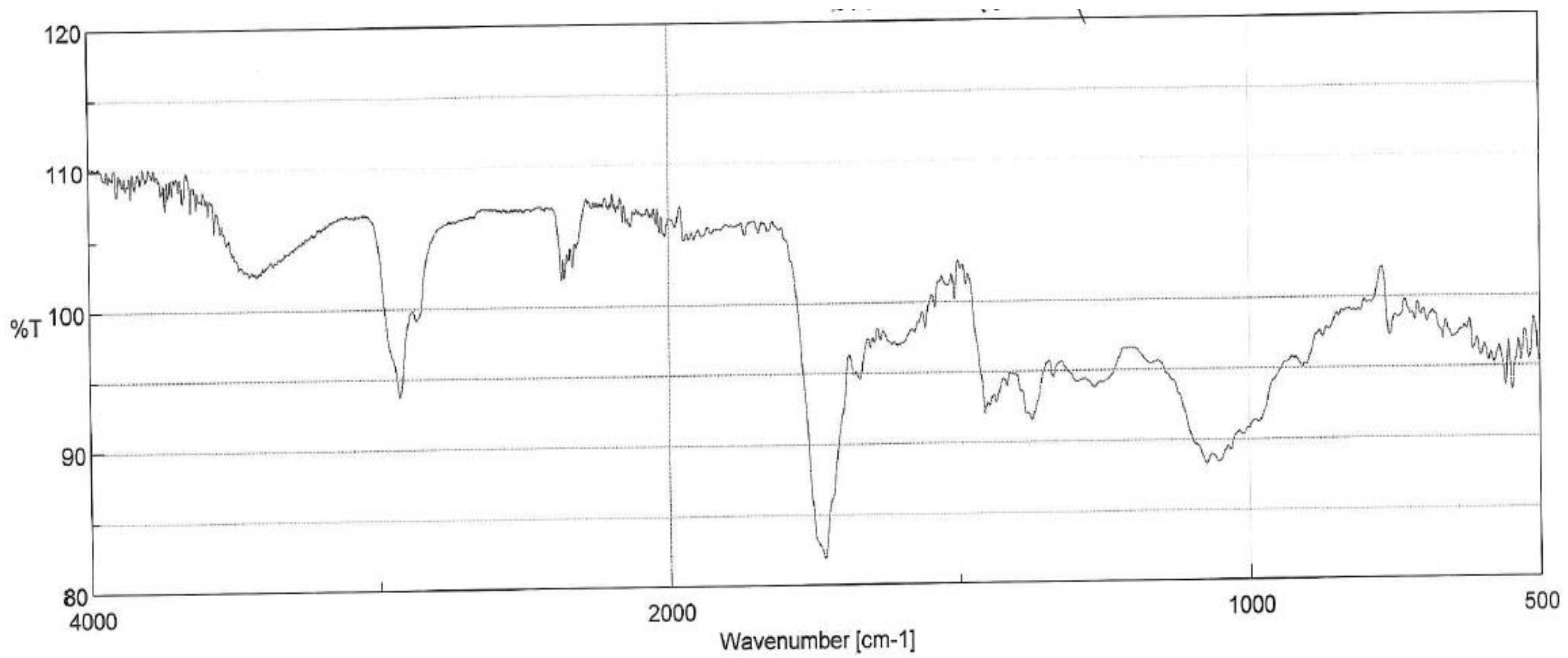

S31: FTIR of **4**

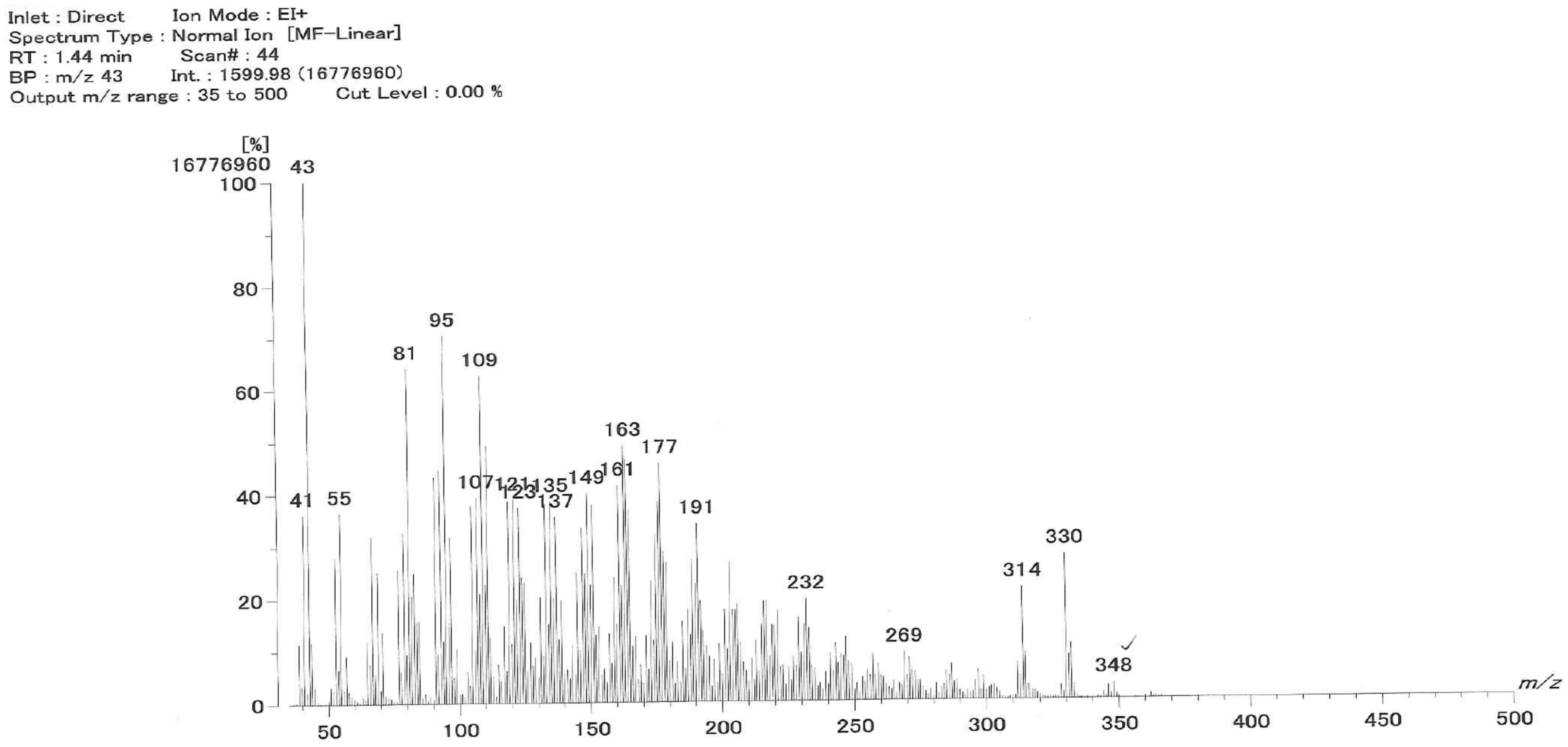

S32: LREIMS of **4**

Inlet : Direct Ion Mode : EI+
RT : 2.08 min Scan# : 53
Elements : C 150/0, H 250/0, O 50/0
Mass Tolerance : 5mmu
Unsaturation (U.S.) : 0.0 – 15.0

	Observed m/z	Int%	Err[ppm / mmu]	U.S.	Composition
1	348.1939	5.37	+0.6 / +0.2	7.0	C20 H28 O5

S33: HRCIMS of **4**

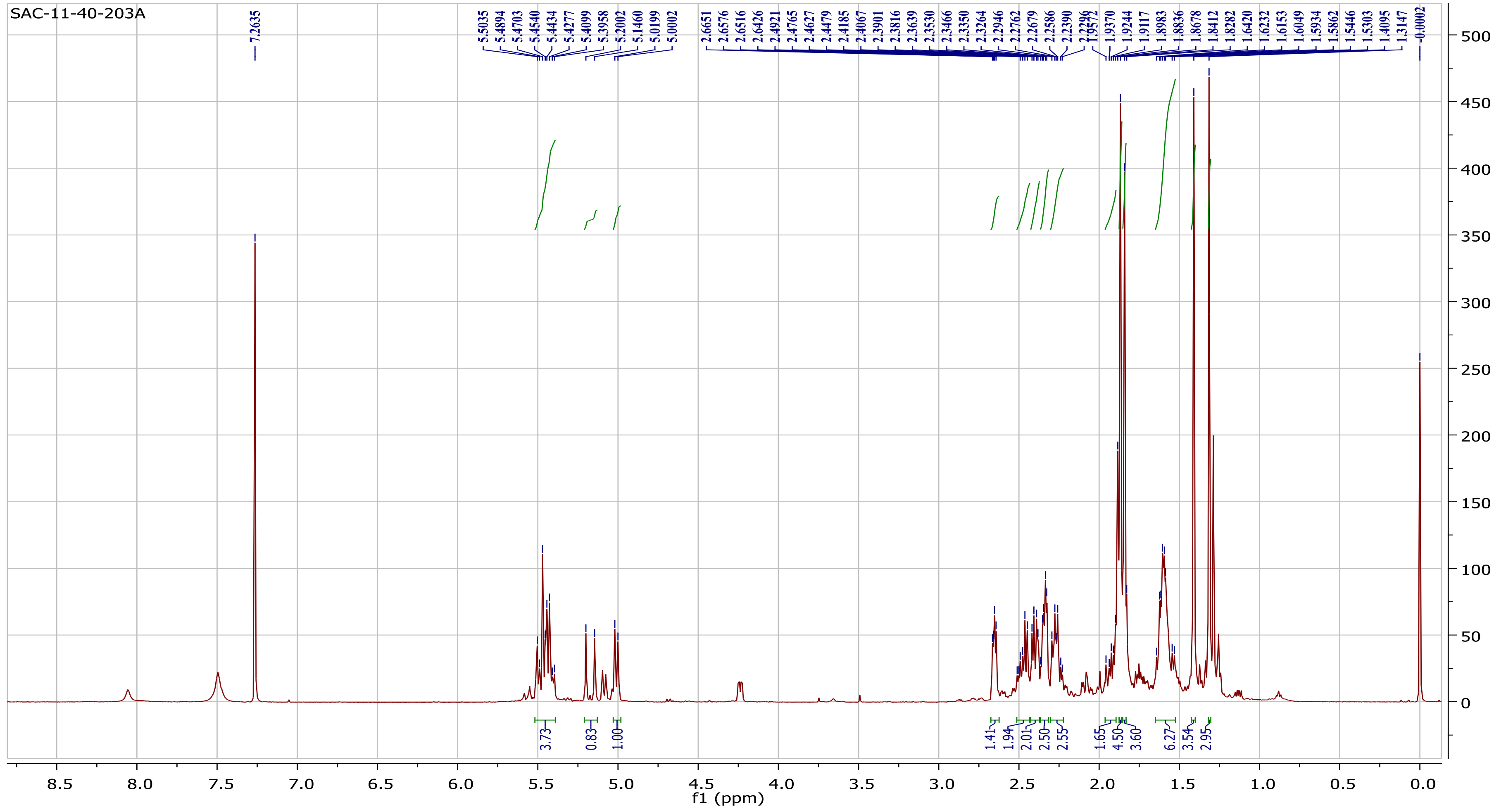

S34: ^{1}H NMR of **4**

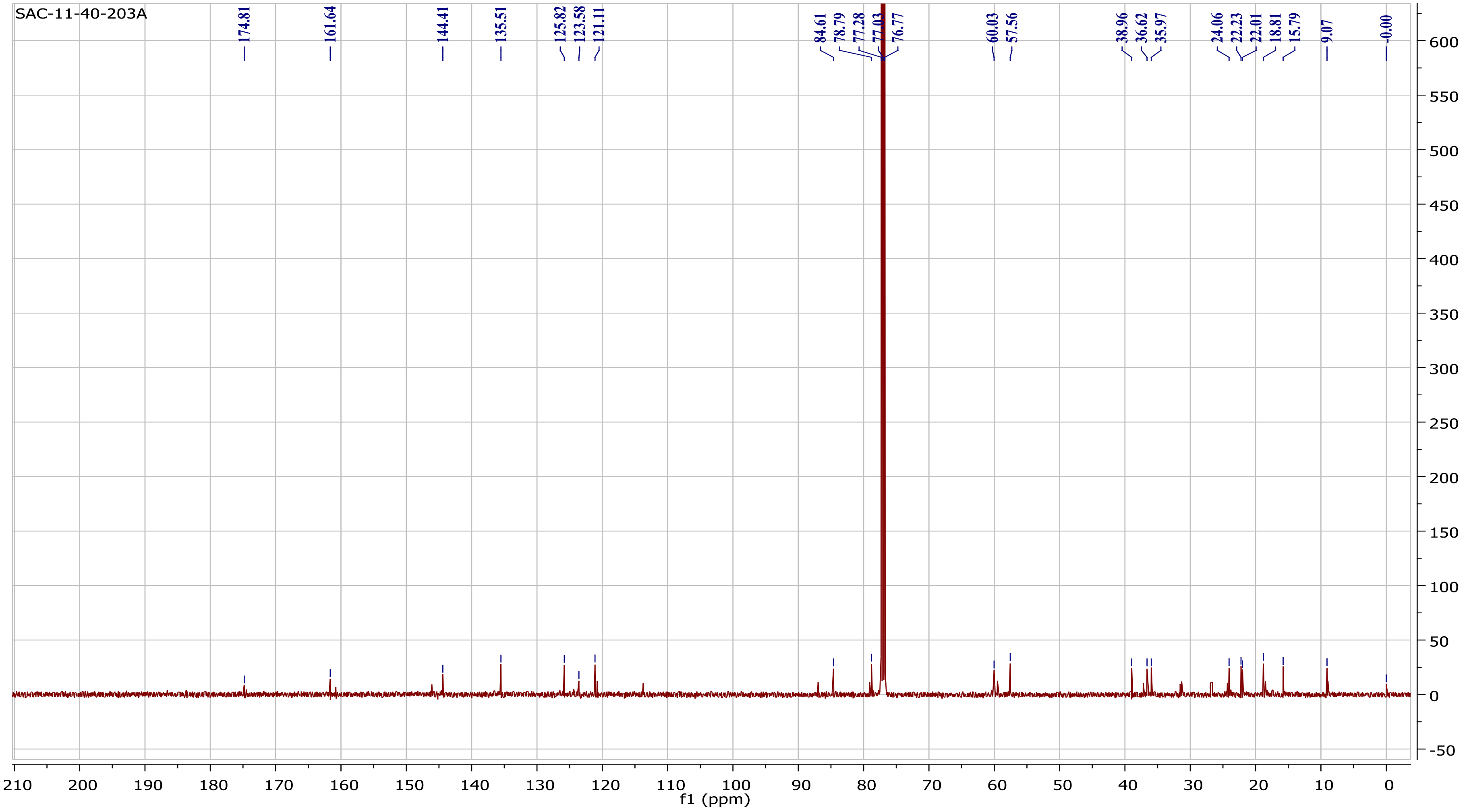

S35: ^{13}C NMR of **4**

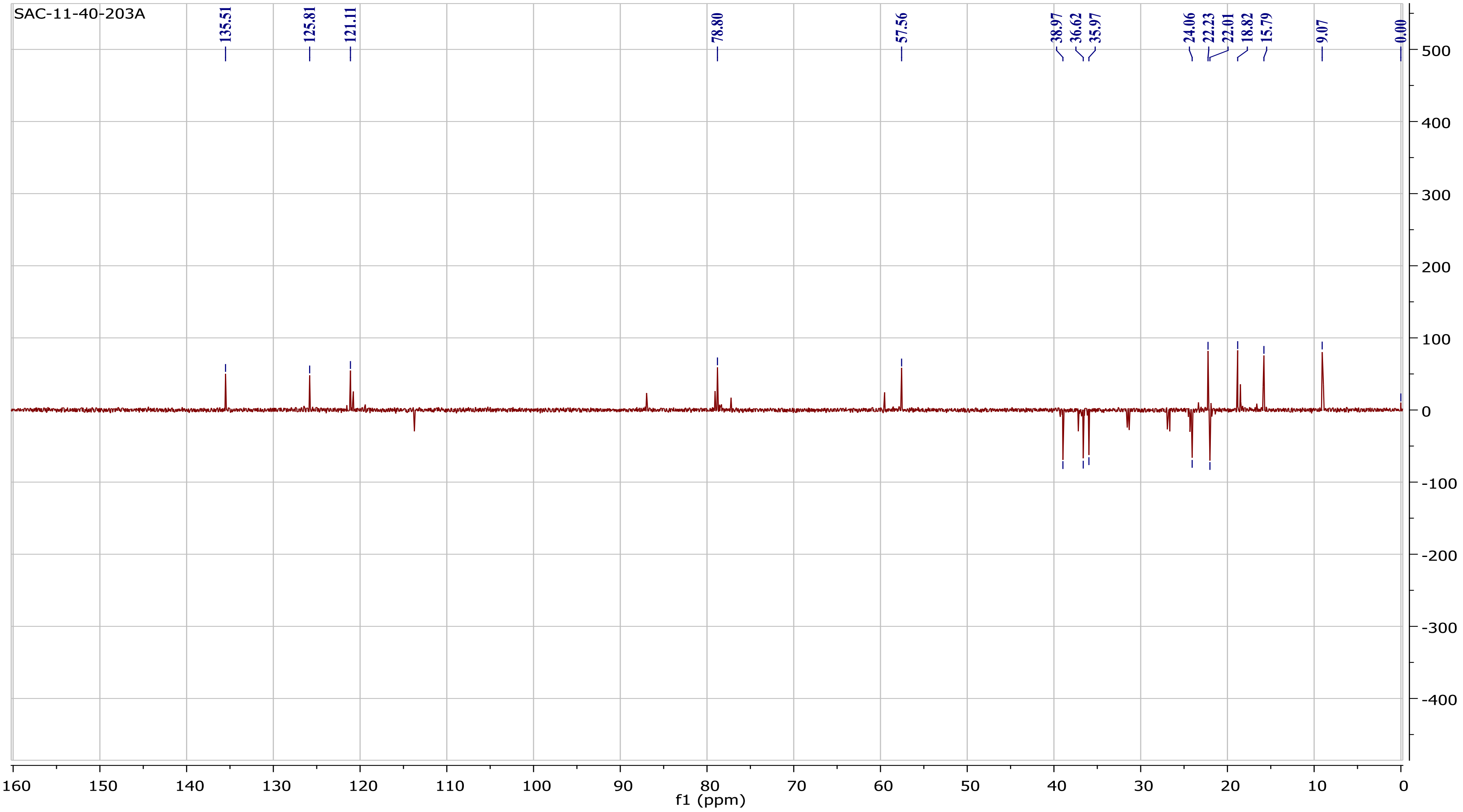
SAC-11-40-203A
135.51
125.81
121.11
78.80
57.56
38.97
36.62
35.97
24.06
22.23
22.01
18.82
15.79
9.07
0.00
500
400
300
200
100
0
-100
-200
-300
-400
160
150
140
130
120
110
100
90
80
70
60
50
40
30
20
10
0
f1 (ppm)

S36: DEPT of **4**

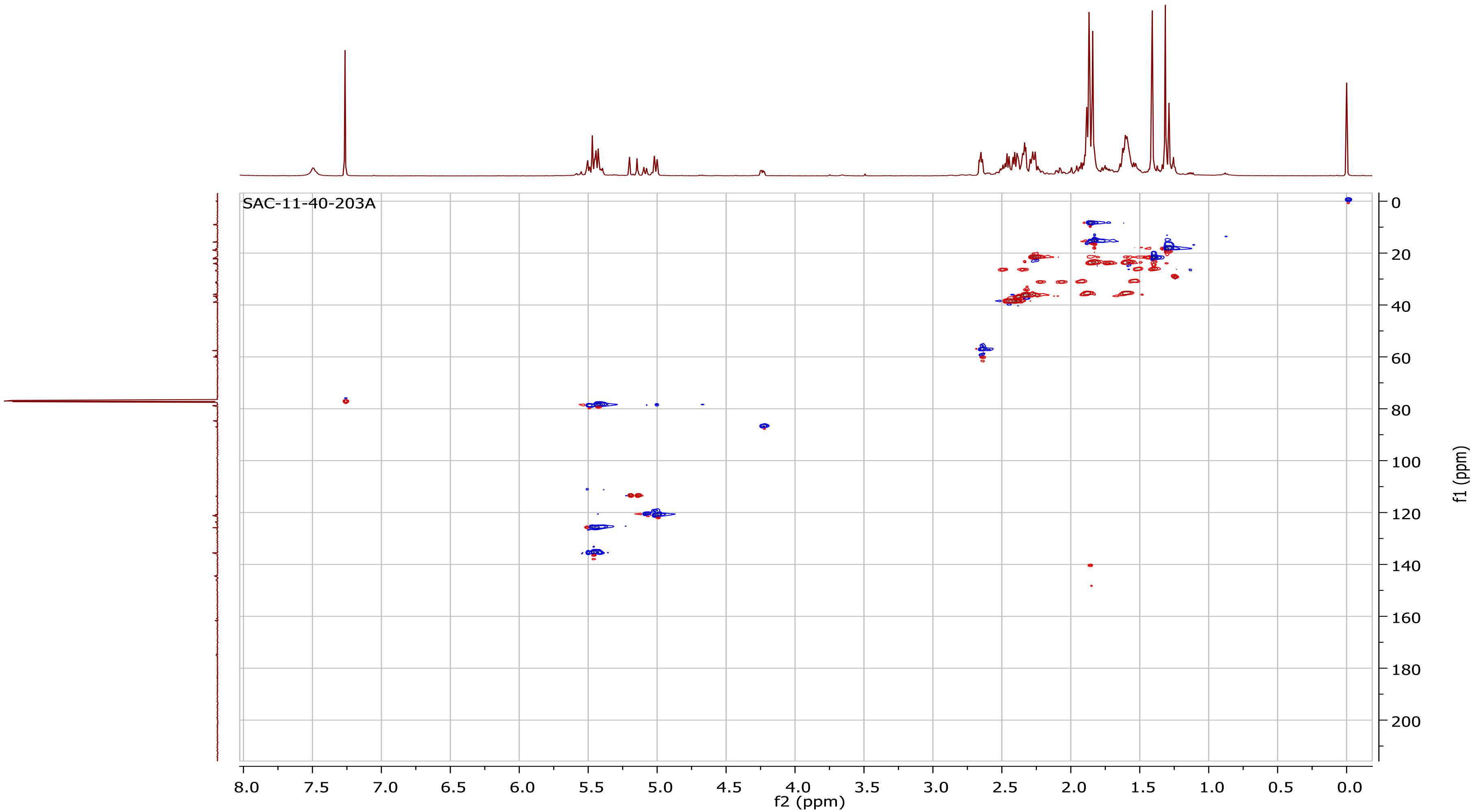

SAC-11-40-203A
8.0
7.5
7.0
6.5
6.0
5.5
5.0
4.5
4.0
3.5
3.0
2.5
2.0
1.5
1.0
0.5
0.0
f2 (ppm)
0
20
40
60
80
100
120
140
160
180
200
f1 (ppm)

S37: HSQC of **4**

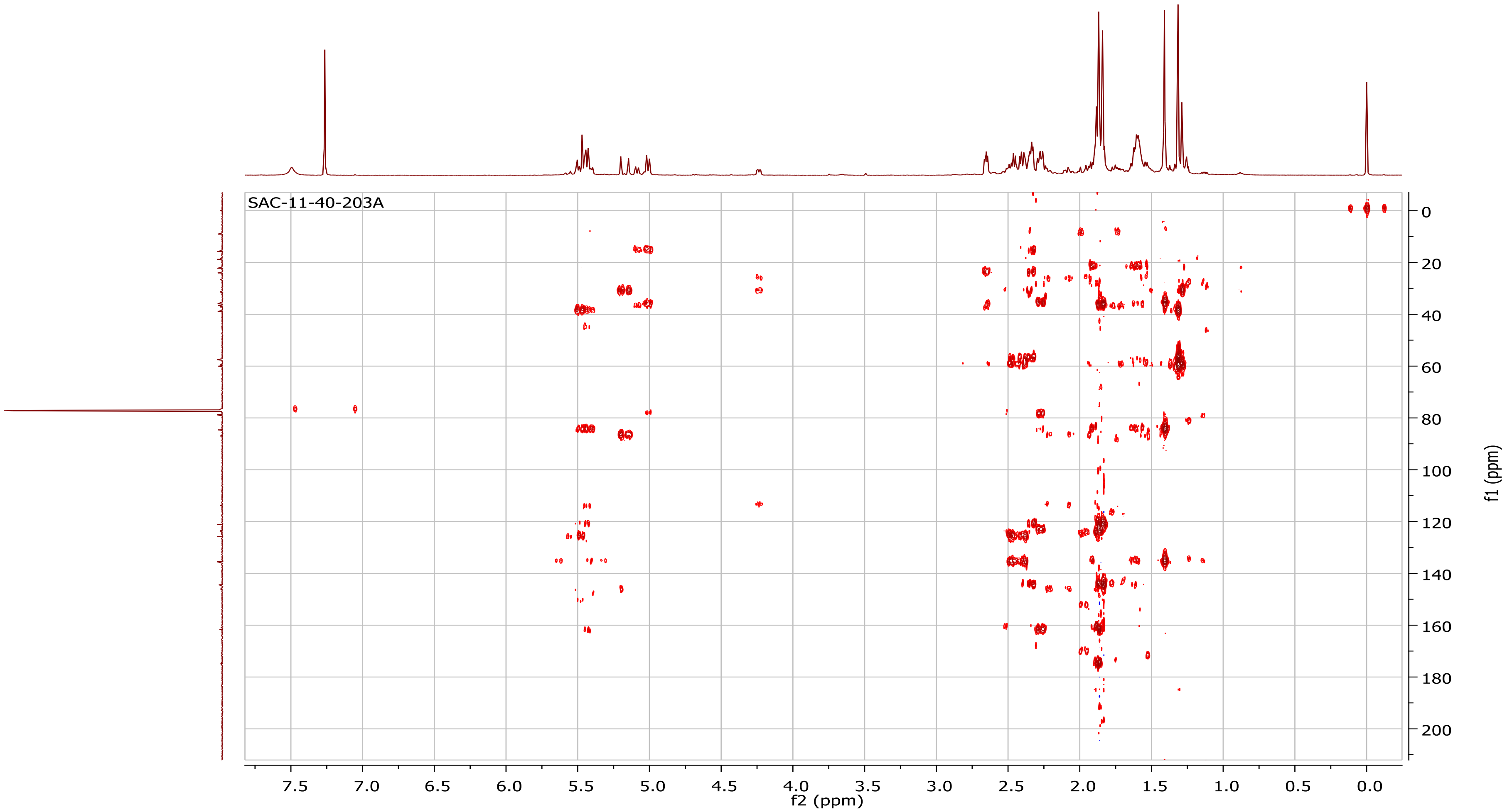

S38: HMBC of **4**

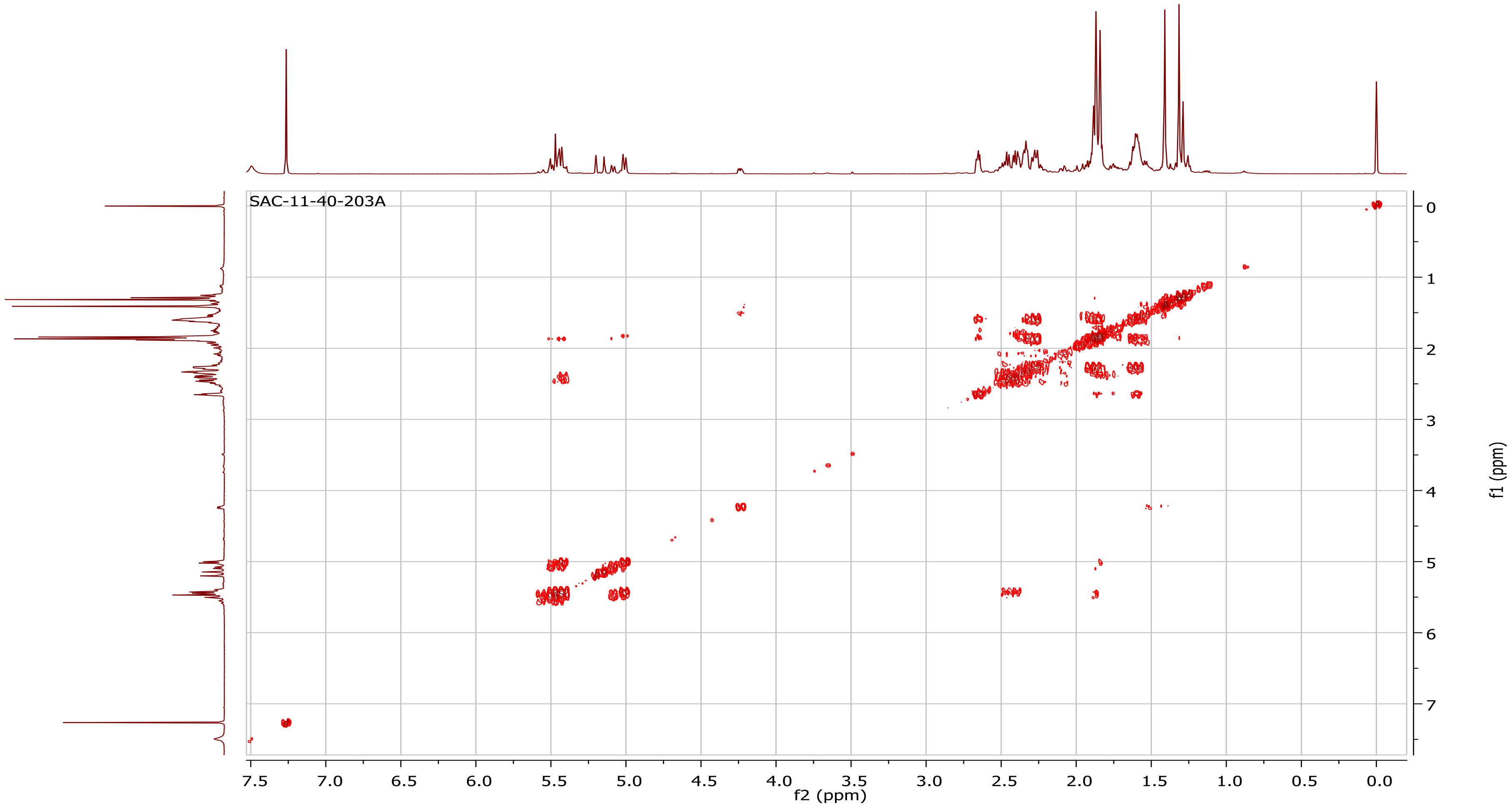

S39: ^{1}H ^{1}H COSY of **4**

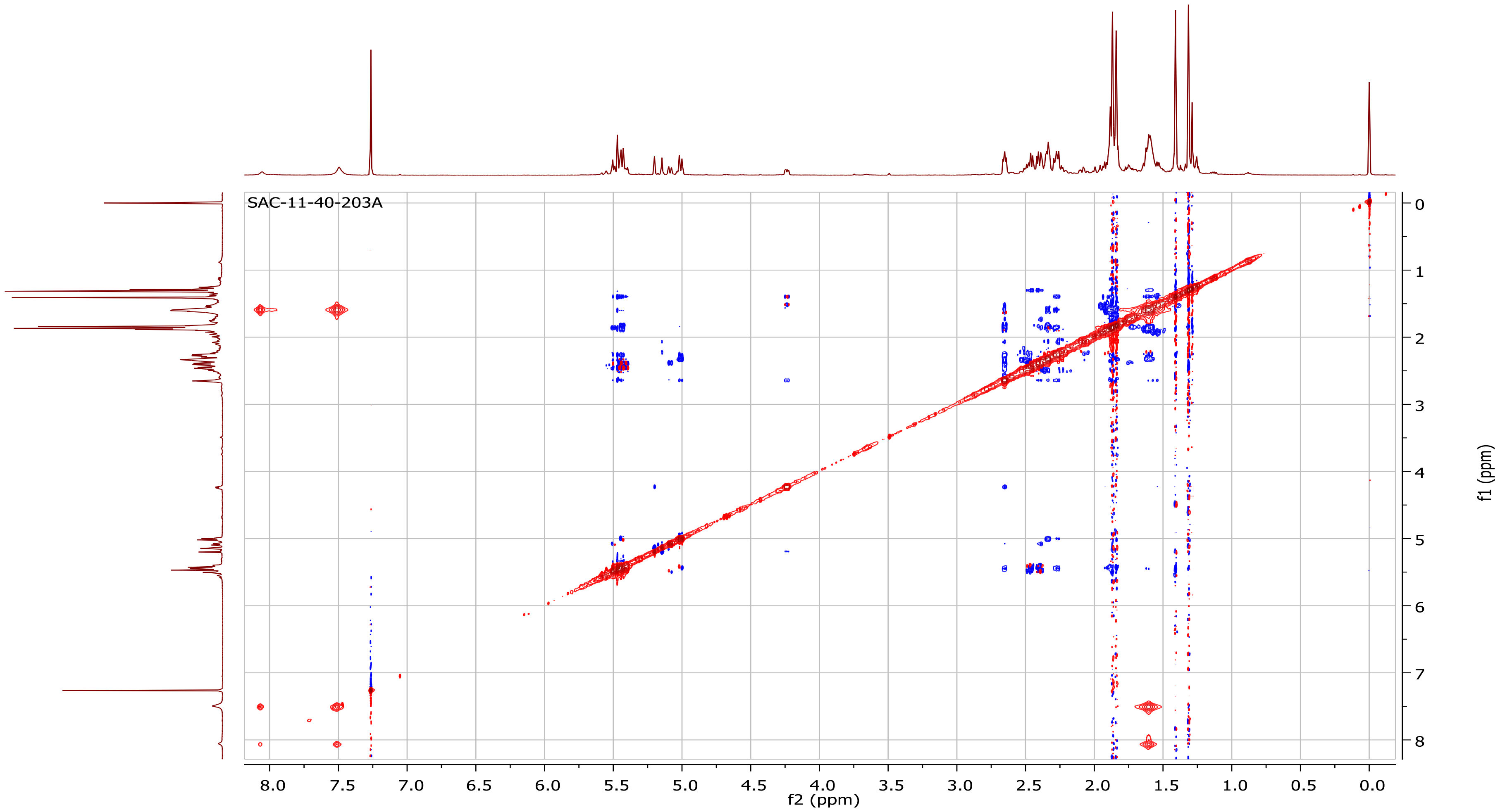

S40: NOESY of **4**

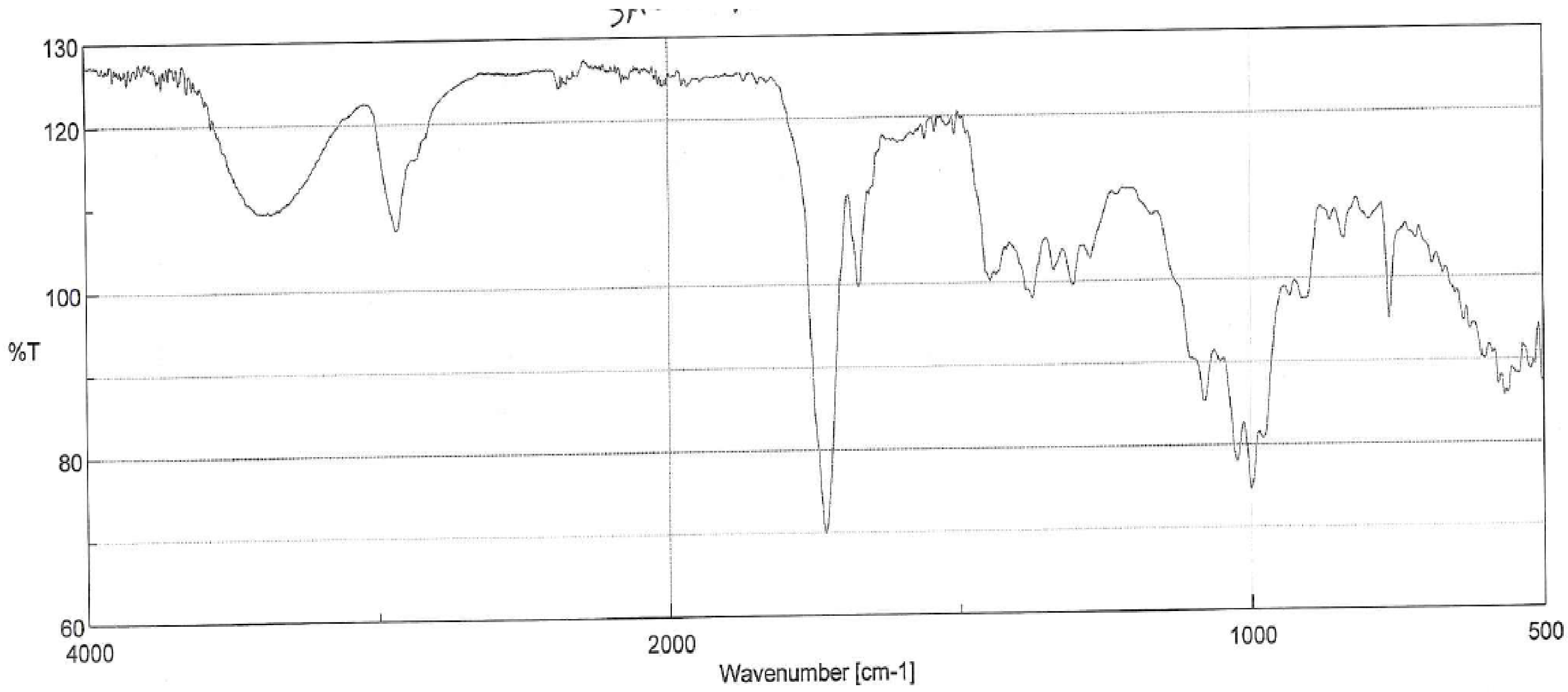

S41: FTIR of **5**

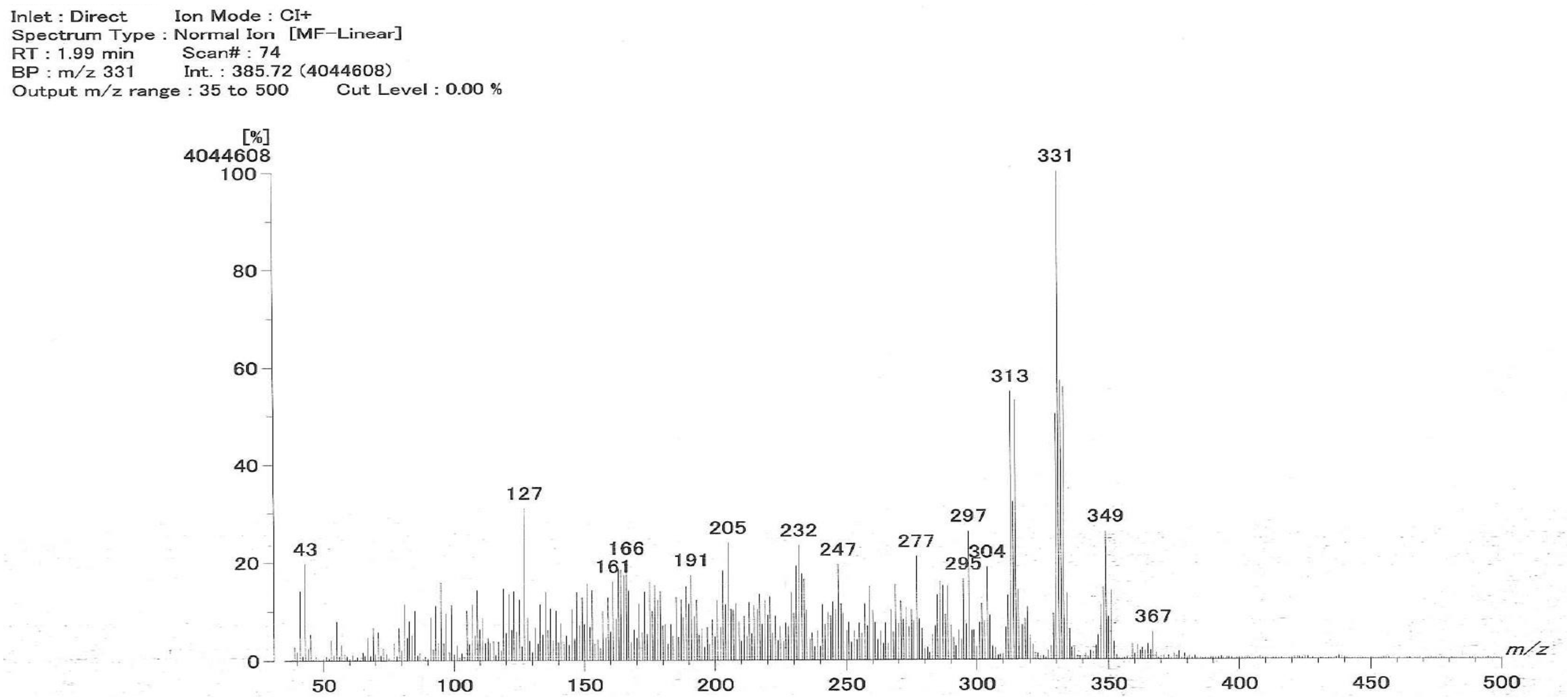

S42: LRCIMS of **5**

Inlet : Direct Ion Mode : CI+
RT : 1.64 min Scan# : 42
Elements : C 150/0, H 250/0, O 50/0
Mass Tolerance : 5mmu
Unsaturation (U.S.) : 0.0 - 15.0

	Observed m/z	Int%	Err[ppm / mmu]	U.S.	Composition
1	367.2130	3.63	+2.5 / +0.9	5.5	C20 H31 O6

S43: HRCIMS of **5**

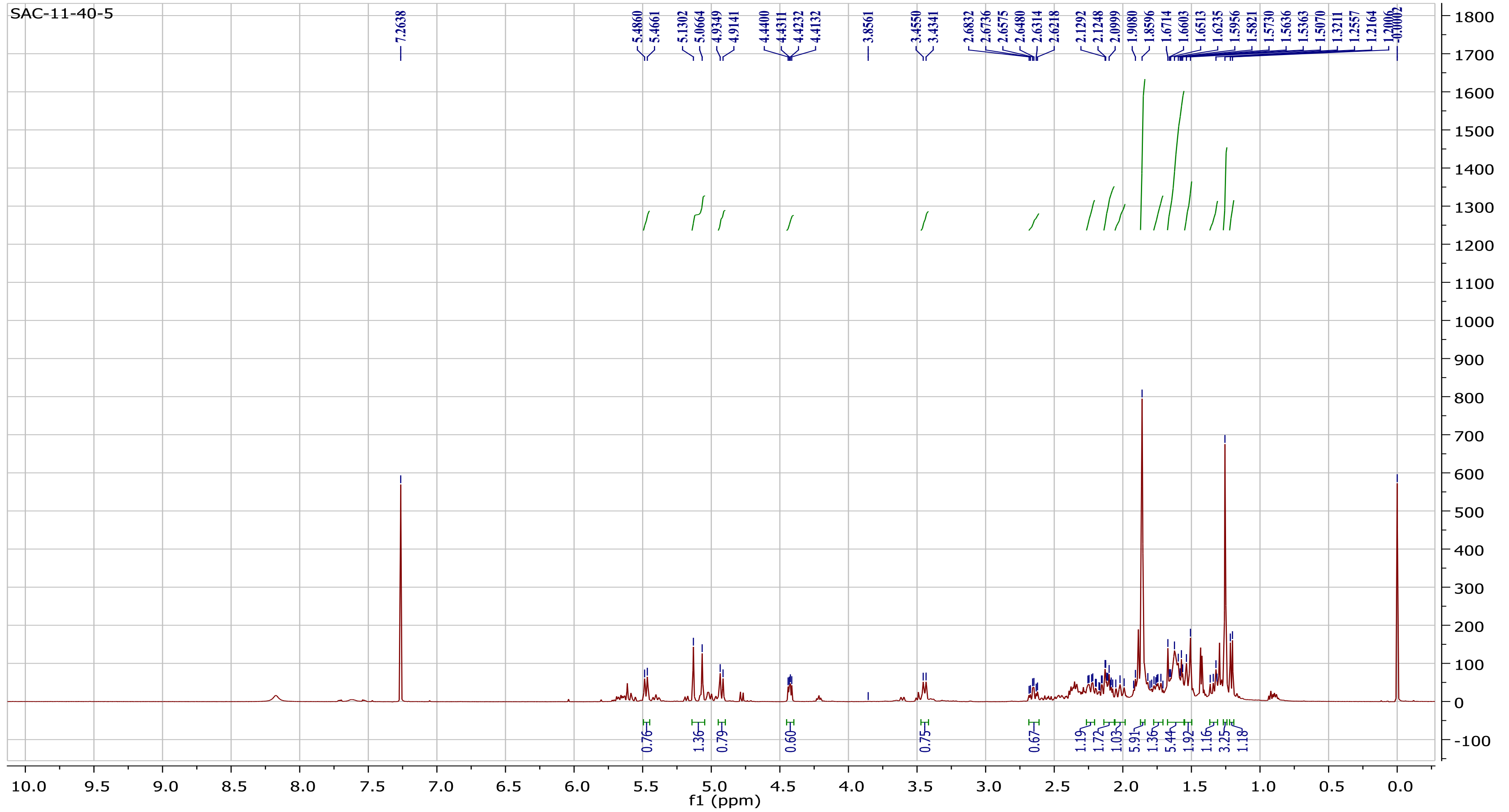

S44: ^{1}H NMR of **5**

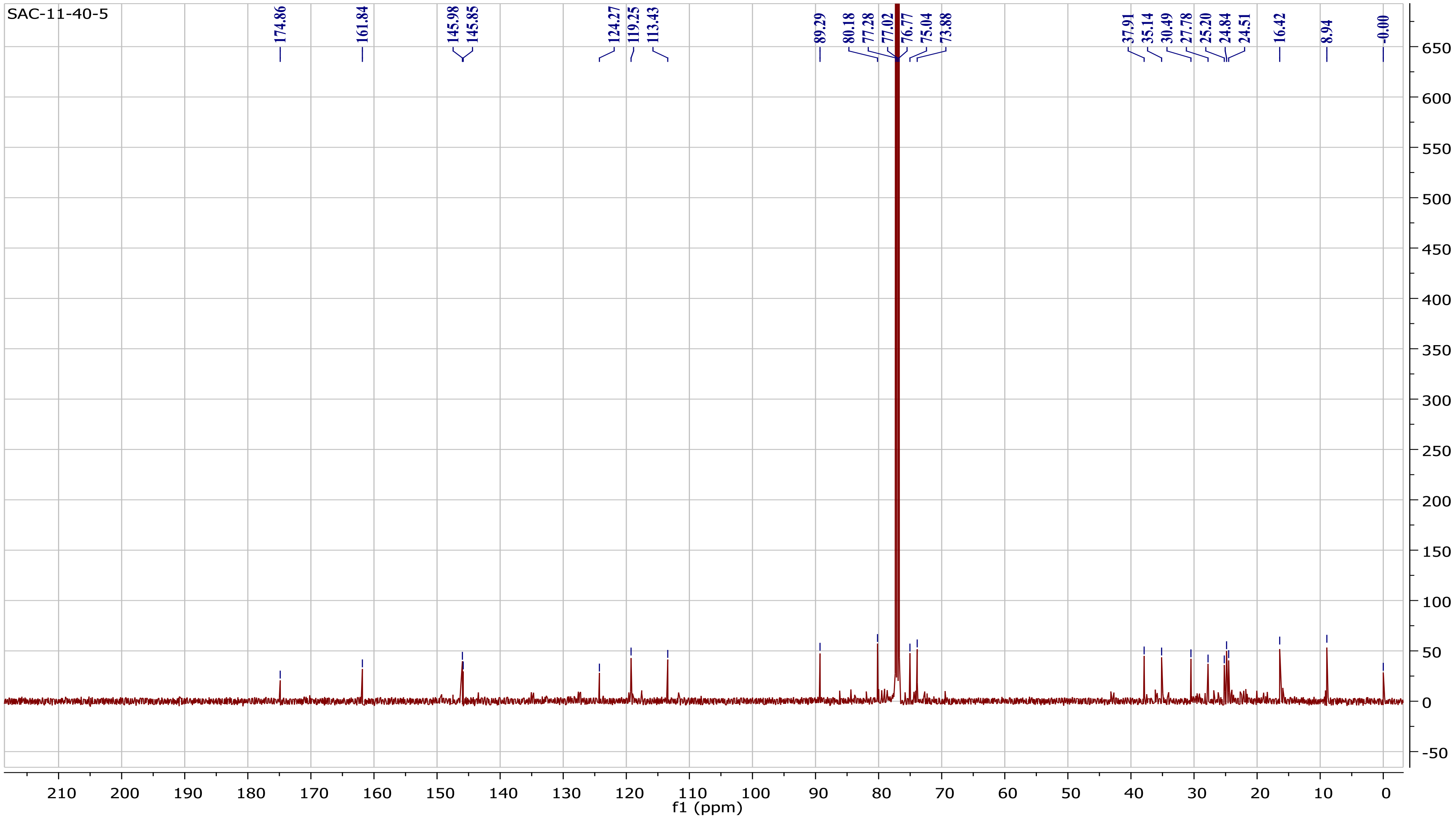

S45: ^{13}C NMR of **5**

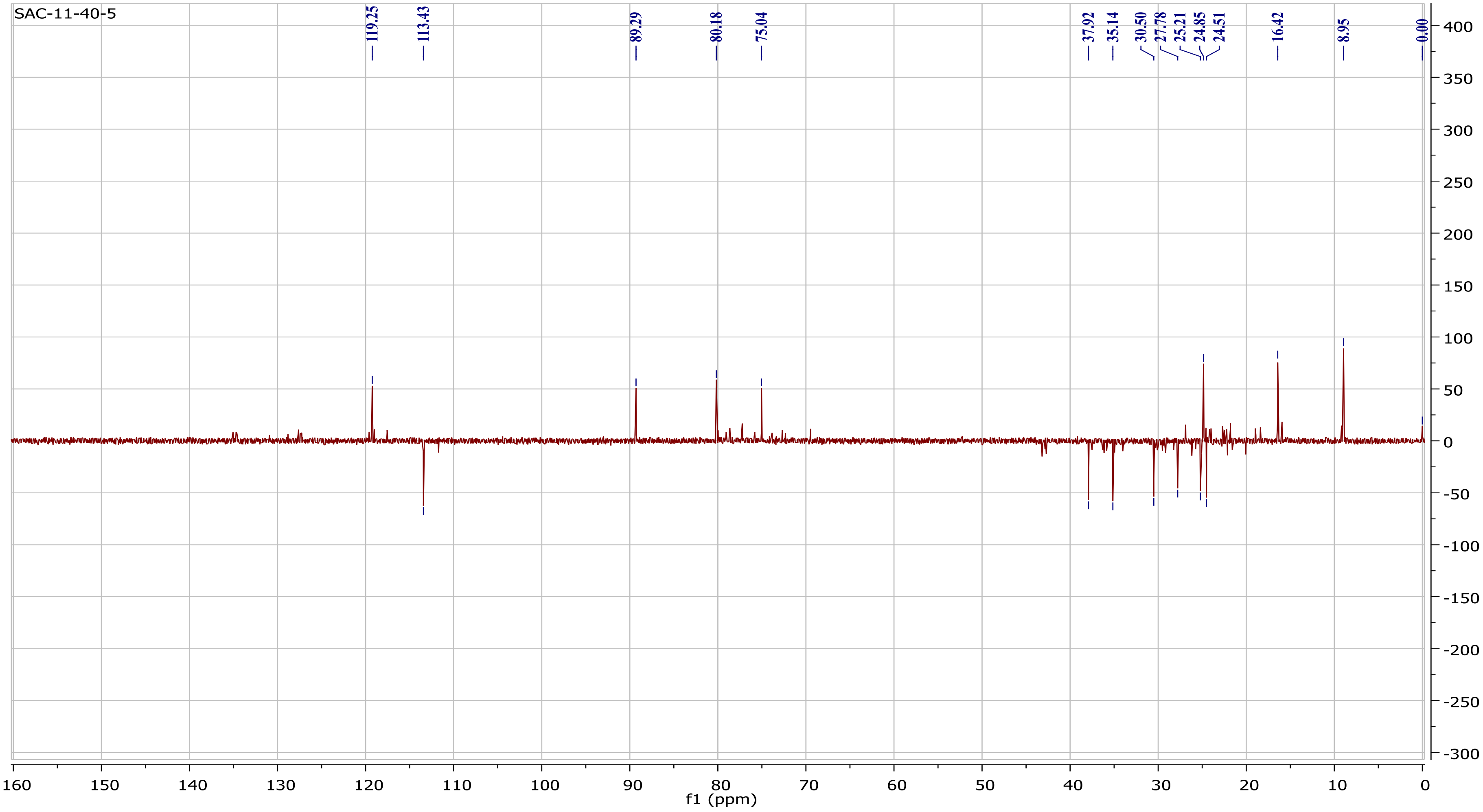
SAC-11-40-5
119.25
113.43
89.29
80.18
75.04
37.92
35.14
30.50
27.78
25.21
24.85
24.51
16.42
8.95
0.00
f1 (ppm)

S46: DEPT of **5**

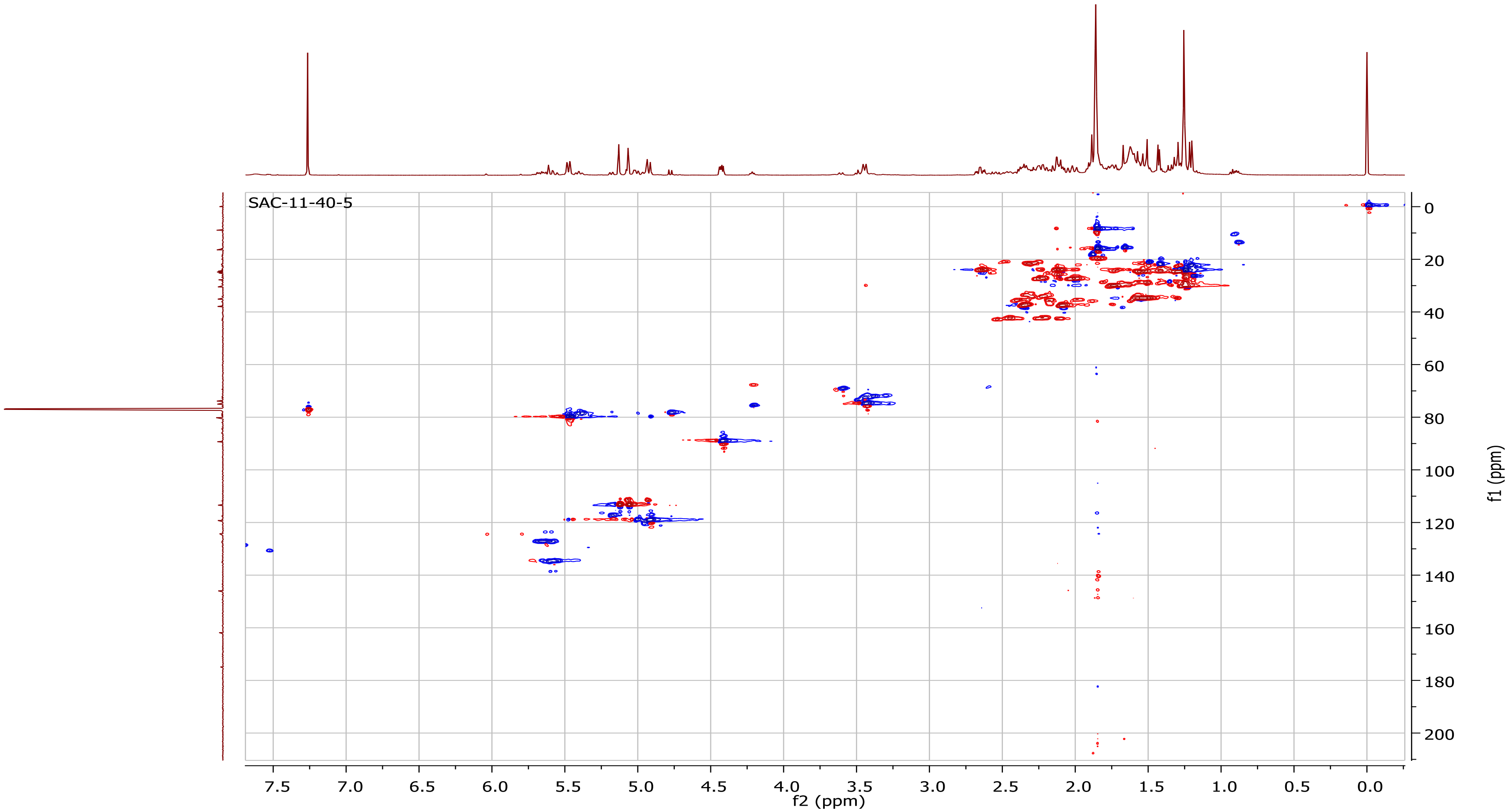

S47: HSQC of **5**

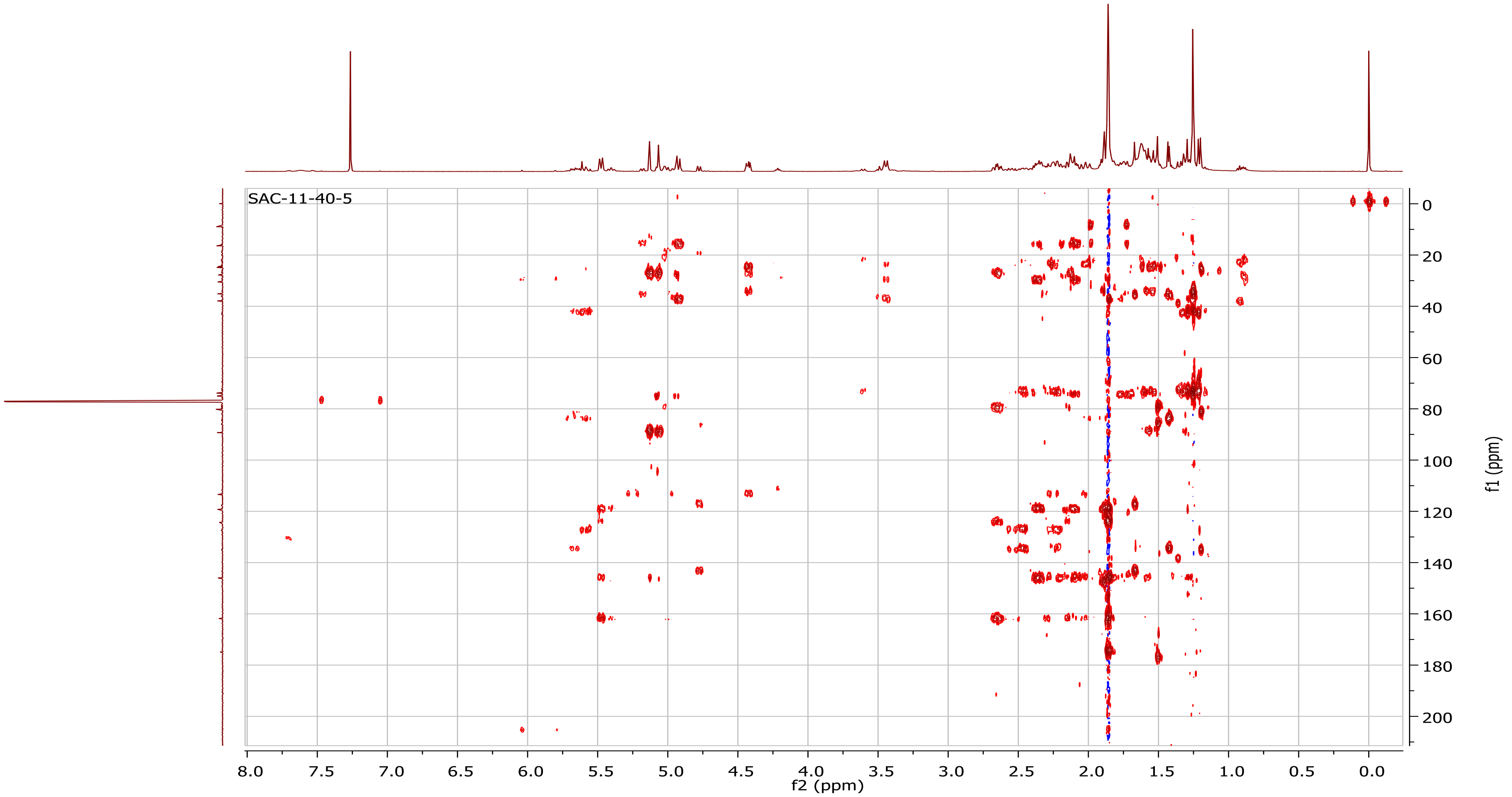

S48: HMBC of **5**

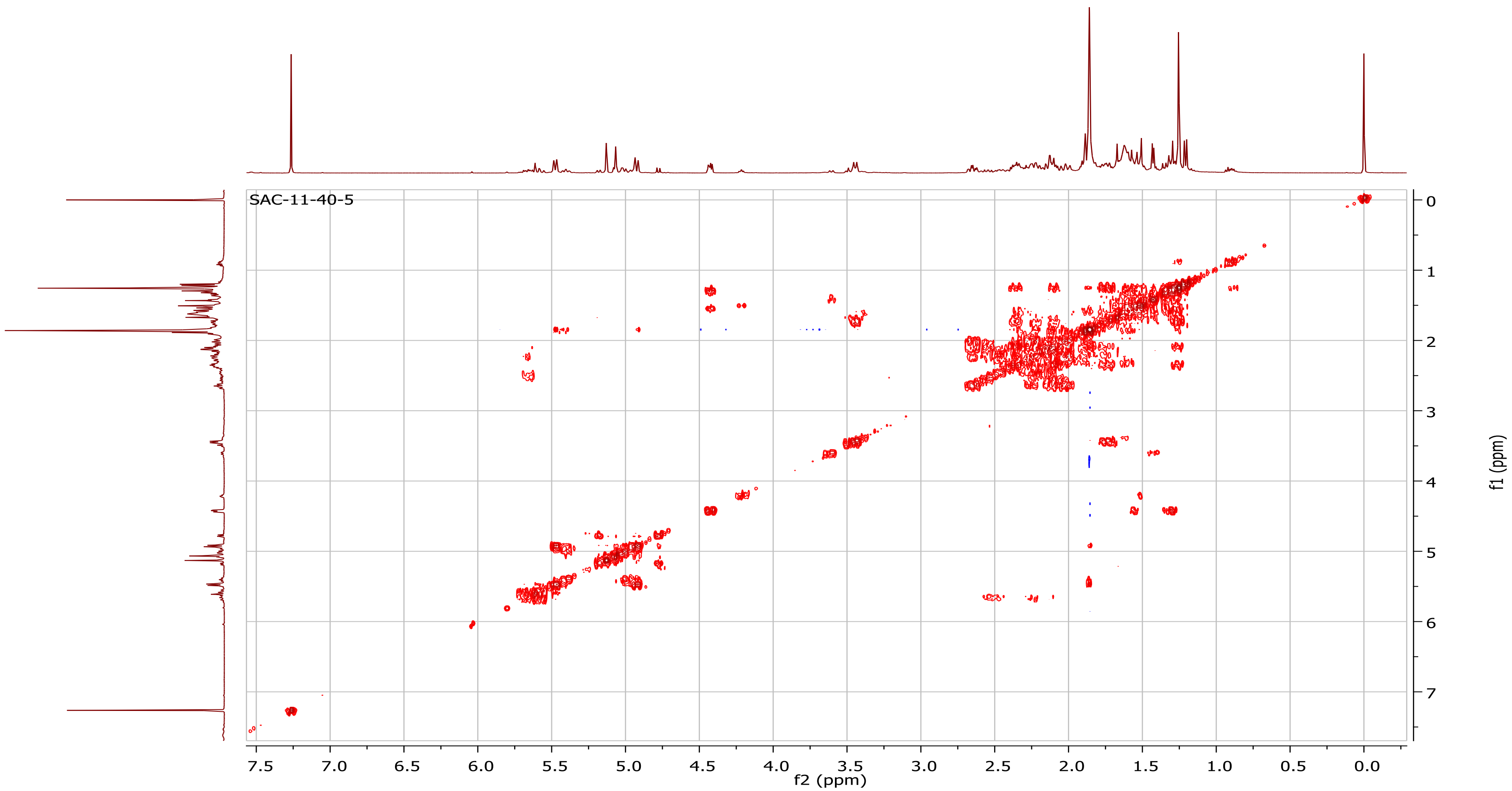

S49: ^{1}H ^{1}H COSY of **5**

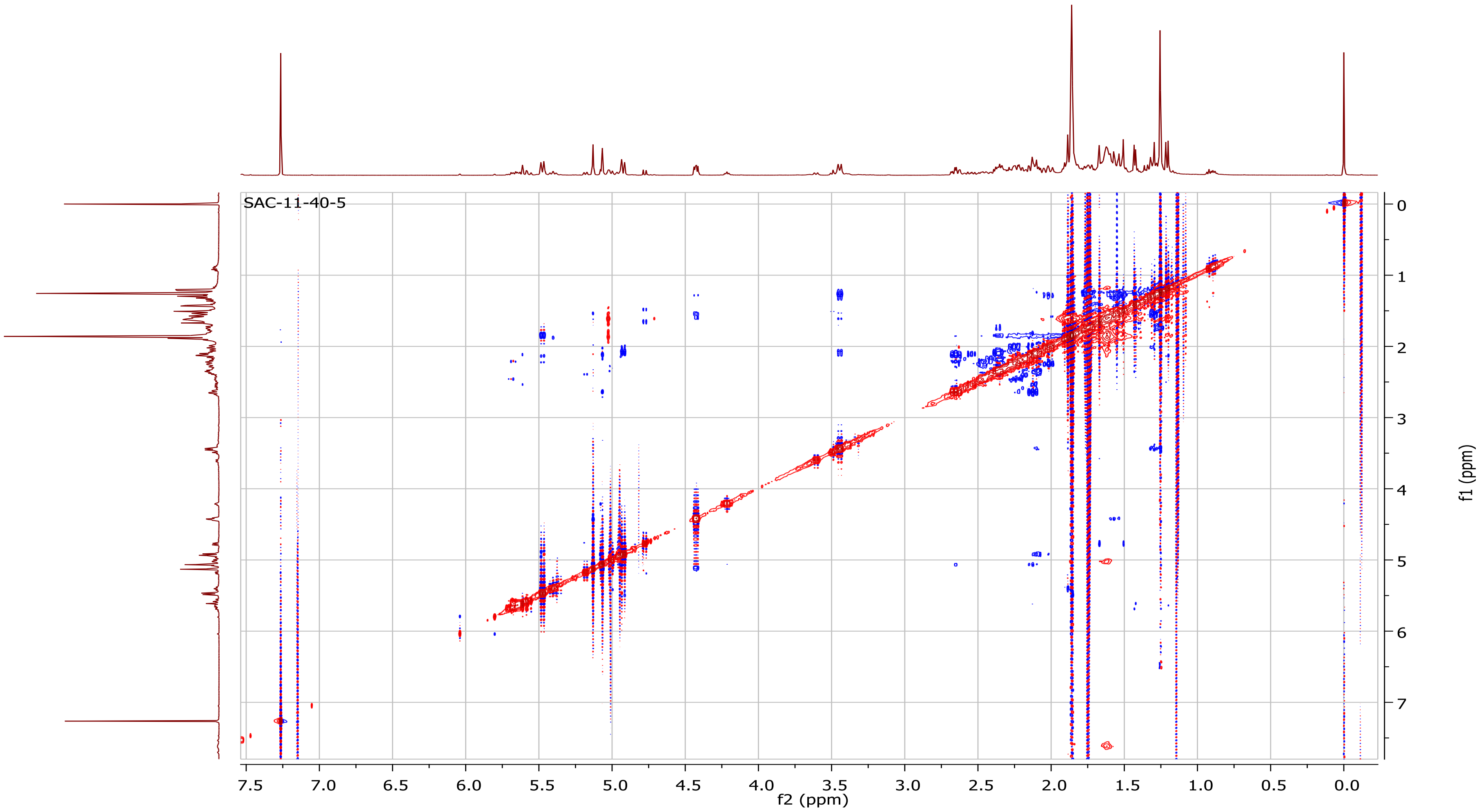

S50: NOESY of **5**
